# The Periplasmic Oxidoreductase DsbA Is Required for Virulence of the Phytopathogen *Dickeya solani*

**DOI:** 10.3390/ijms23020697

**Published:** 2022-01-09

**Authors:** Tomasz Przepiora, Donata Figaj, Aleksandra Bogucka, Jakub Fikowicz-Krosko, Robert Czajkowski, Nicole Hugouvieux-Cotte-Pattat, Joanna Skorko-Glonek

**Affiliations:** 1 Department of General and Medical Biochemistry, Faculty of Biology, University of Gdansk, Wita Stwosza 59, 80-308 Gdansk, Poland; tomasz.przepiora@ug.edu.pl (T.P.); donata.figaj@ug.edu.pl (D.F.); 2 Laboratory of Mass Spectrometry, Intercollegiate Faculty of Biotechnology, University of Gdansk and Medical University of Gdansk, Abrahama 58, 80-807 Gdansk, Poland; aleksandra.lewandowska@ug.edu.pl; 3 Laboratory of Biologically Active Compounds, Intercollegiate Faculty of Biotechnology, University of Gdansk and Medical University of Gdansk, Abrahama 58, 80-807 Gdansk, Poland; fikowicz.krosko@gmail.com (J.F.-K.); robert.czajkowski@ug.edu.pl (R.C.); 4 Microbiologie Adaptation et Pathogénie, Université Lyon, CNRS, INSA Lyon, Université Claude Bernard Lyon 1, Campus LyonTech-la Doua Bâtiment André Lwoff 10 rue Raphaël Dubois 69622, F69622 Villeurbanne, France; nicole.cotte-pattat@insa-lyon.fr

**Keywords:** plant pathogen, *Dickeya solani*, virulence factors, plant infection, oxidoreductase DsbA, disulfide bonds, proteomic analysis, secretome

## Abstract

In bacteria, the DsbA oxidoreductase is a crucial factor responsible for the introduction of disulfide bonds to extracytoplasmic proteins, which include important virulence factors. A lack of proper disulfide bonds frequently leads to instability and/or loss of protein function; therefore, improper disulfide bonding may lead to avirulent phenotypes. The importance of the DsbA function in phytopathogens has not been extensively studied yet. *Dickeya solani* is a bacterium from the Soft Rot *Pectobacteriaceae* family which is responsible for very high economic losses mainly in potato. In this work, we constructed a *D. solani dsbA* mutant and demonstrated that a lack of DsbA caused a loss of virulence. The mutant bacteria showed lower activities of secreted virulence determinants and were unable to develop disease symptoms in a potato plant. The SWATH-MS-based proteomic analysis revealed that the *dsbA* mutation led to multifaceted effects in the *D. solani* cells, including not only lower levels of secreted virulence factors, but also the induction of stress responses. Finally, the outer membrane barrier seemed to be disturbed by the mutation. Our results clearly demonstrate that the function played by the DsbA oxidoreductase is crucial for *D. solani* virulence, and a lack of DsbA significantly disturbs cellular physiology.

## 1. Introduction

The success of plant host infection by phytopathogenic bacteria depends on the coordinated action of numerous virulence factors and virulence determinants. In most cases, these factors are extracellular secreted proteins or proteins associated with the surface of a pathogen’s cell [1]. The ability to withstand unfavorable environmental conditions (both in and outside of the host) is an equally important factor. As an outer part of the bacterium, the bacterial cellular envelope is particularly exposed to fluctuations of the environment. Therefore, the ability of bacteria to survive various external stress factors is strictly linked with the correct functioning of the envelope as the cell/environment barrier. Since secreted or envelope proteins are particularly exposed to harsh environmental conditions, which may result in their rapid denaturation, their structure is frequently stabilized by disulfide (S-S) bonds formed between cysteine residues. Conversely, a lack of proper disulfide bonds in these proteins frequently results in unfolding, loss of function and subsequent protein degradation. In bacteria, oxidoreductases of the Dsb (Disulfide bond) protein family are responsible for introducing proper S-S bonds between cysteine residues [2,3,4], reviewed in [5].

In the model Gram-negative bacterium *Escherichia coli*, the cysteine residues of exported proteins are oxidized by the DsbA/DsbB system in the periplasm [6]. Proteins that contain more than two cysteine residues frequently need rearrangement of the S-S bonds, and the DsbC/DsbD isomerase system catalyzes this process. Additionally, DsbE and DsbG proteins act as specialized reducing enzymes [7].

The substrates of DsbA comprise a large variety of proteins whose functions are necessary to maintain cellular homeostasis as well as proteins associated with bacterial pathogenesis. The latter include diverse bacterial virulence factors, for example, toxins, secretion systems, adhesins and flagellar motility machinery. As a result, bacterial cells deprived of the functional *dsbA* gene exhibit a pleiotropic phenotype, frequently show reduced fitness in animal infection models and/or display attenuated virulence, reviewed in [5,8]. The vast majority of previous studies concerning Dsb proteins were performed in *E. coli*. Likewise, only a few animal/human bacterial pathogens (e.g., *Burkholderia pseudomallei*, uropathogenic *E. coli* or *Salmonella enterica* Typhimurium) were used to assess the role of Dsb proteins in the interaction between these bacteria and their hosts [8]. In contrast, knowledge about the involvement of these enzymes in the interaction between phytopathogenic bacteria and their hosts is still very limited.

According to the published genome sequence [9] and Uniprot [10] analysis, *Dickeya solani* generally encodes all components of the archetypal Dsb system from *E. coli* K12: DsbA/DsbB (oxidative pathway), DsbC/DsbD (isomerization pathway) as well as DsbG as a specialized reducing enzyme (a gene coding for DsbE seems to be absent in *D. solani*). The DsbA protein from *D. solani* (thiol:disulfide interchange protein of *D. solani* RNS 08.23.3.1.A; UniProtID: A0A2K8W006) shares 66.8% amino acid sequence identity and 82.2% similarity with *E. coli* strain K12 DsbA (UniProtID: P0AEG4) (EMBOSS Needle sequence alignment tool). The sizes of both DsbA homologs are similar as well (208 and 213 amino acids in *D. solani* and *E. coli*, respectively), and the composition of their catalytic sites is identical (CPHC). Active site cysteine residues are localized at positions 49 and 52 (*E. coli*) and 55 and 58 (*D. solani*).

This work aimed to assess, in detail and at the molecular level, the role of the protein DsbA in the environmental fitness and virulence of a member of the Soft Rot *Pectobacteriaceae* (SRP) group, *D. solani*, during its interaction with its natural host plant, potato. The SRP bacteria cause significant losses in various crops and ornamentals worldwide and therefore were included among the top ten most important plant pathogens [11]. In Europe, the SRPs cause economic losses mainly in potato, estimated to be approximately 46 M Euro annually [12]. Among the many SRP species, *D. solani* has recently been recognized as one of the most virulent plant pathogens. *D. solani* causes the rotting of tubers in the field and under storage conditions and the blackleg disease of stems of field-growing potato plants. Compared to other *Dickeya* species, *D. solani* causes more severe symptoms in potato plants, has a wider optimal temperature range for growth and disease development and can develop blackleg disease from lower inoculum levels [13]. Furthermore, over the past 20 years, *D. solani* became the most abundant *Dickeya* species isolated from infected potato plants in Europe [14,15,16,17,18,19,20].

The SRP group of plant pathogenic bacteria, including *D. solani*, adopted an invasion strategy based on the massive production of extracellular virulence factors whose role is to degrade components of the plant cell wall (plant-cell-wall-degrading enzymes; PCWDEs). The essential PCWDEs of SRPs are pectin-degrading enzymes (pectinases), cellulases and proteases, which are directly responsible for decomposing plant tissues. Pectinases are a group of enzymes indispensable for bacterial virulence, comprising pectate lyases, endopolygalacturonases, pectin lyases and pectin esterases. Among them, pectate lyases are considered the most important, and they cause the rapid degradation of pectins and polygalacturonic acid, reviewed in [21]. Some literature reports suggest the necessity of DsbA for the activity of PCWDEs. For example, in *Dickeya dadantii* 3937 (formerly *Erwinia chrysanthemi* 3937), DsbA is required for the stability and secretion of several pectate lyases and of the cellulase CelZ [22]. Studies performed on *Pectobacterium atrosepticum* (formerly *Erwinia carotovora* subsp. *atroseptica*) demonstrated that DsbA deficiency impairs the production of almost all secreted virulence factors. As a result, the secretome of the *P. atrosepticum dsbA* mutant lacked the major secreted proteins. Likewise, the total amount of secreted proteins was lower than in the wild-type (wt) strain [23].

The plant host infection caused by the SRPs can be divided into three main steps: (I) the adhesion of bacterial cells to the plant surface and their entry into plant tissues, (II) colonization of the apoplast and finally (III) degradation of the plant cell wall components, resulting in the development of visible disease symptoms. Therefore, to successfully colonize and cause disease symptoms in the host, bacteria need to coordinate the expression and activity of many virulence factors. In the first stage of interaction (adhesion to host cell and entry into plant tissue), the motility and chemotaxis of the pathogen play a crucial role. These determinants are essential for bacteria when searching for favorable sites to adhere to and enter the plant through. For this reason, non-motile *D. dadantii* mutants encounter difficulties in penetrating the plant tissue and in establishing a systemic infection [24]. There are two major types of bacterial movement, swimming and swarming; both types depend on functional flagella and the sensing of chemical signals (attractants or repellents). Swimming cells perceive chemical signals via methyl-accepting chemotaxis proteins and trigger appropriate chemotactic responses to the signals [25]. Swarming is a movement of tight groups of bound bacterial cells on semisolid surfaces [26] and is associated with a differentiation process of bacterial cells [27,28]. During the colonization of the apoplast, bacteria must adjust to rather harsh environmental conditions, including plant cell defense responses and the low availability of iron ions, which are crucial cofactors of many enzymes. For this purpose, bacteria produce additional virulence determinants, including systems related to iron acquisition (siderophores and iron storage proteins) and the reactive oxygen species scavenger indigoidine [29,30].

In this work, we examined the importance of the oxidoreductase DsbA for the growth, virulence and activity of essential virulence determinants of the plant pathogenic *D. solani* type strain, IPO2222. We also undertook a global investigation into the role of DsbA in maintaining cellular proteostasis and protein secretion. We found that the *dsbA* mutants showed no growth defects under stressful conditions but were unable to infect plants and to produce disease symptoms. Proteomic analysis revealed a global impact of the *dsbA* inactivation on the *D. solani* proteome, involving the envelope and both cytoplasmic and secreted proteins. In addition, the *dsbA* mutant was deprived of most secreted virulence factors, including PCWDEs, siderophores and flagellar proteins. To our knowledge, this is the first such extensive work focused on the function of DsbA in a bacterial phytopathogen of the genus *Dickeya*.

## 2. Results

### 2.1. Inactivation of the dsbA Gene Does Not Cause an Increased Sensitivity to Stressful Conditions

To investigate the potential contribution of the DsbA protein in *D. solani* fitness and virulence, the *dsbA* gene of the *D. solani* IPO2222 chromosome was inactivated by marker exchange mutagenesis to produce the *D. solani dsbA::cm^R^* mutant (TP02) (Figure S1). To ensure that the potential differences between the mutant and parental strains were solely due to the presence/absence of the DsbA function, we constructed a complemented strain (TP03), where the wt *dsbA* gene was returned into its native location in the *D. solani* chromosome.

To evaluate the phenotypic effects of the *dsbA* mutation, the growth curves of the *D. solani dsbA* mutant, complemented and parental *D. solani* IPO2222 strains were determined. The *dsbA* mutant did not show any growth defects compared to the control strains when grown under physiological conditions (Figure S2A). Next, the phenotype of *D. solani dsbA* was evaluated under stressful conditions: heat shock, osmotic/saline, oxidative and acid stresses. We found that the inactivation of DsbA did not enhance the negative effects of either stressor (Figures S2B, S3–S6).

**Figure 1:**
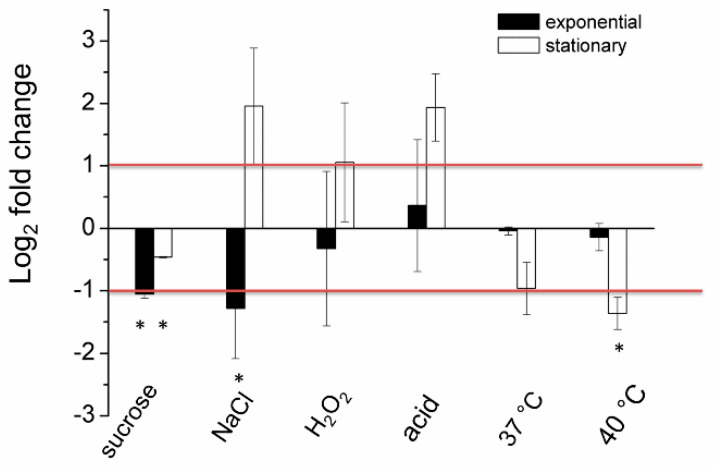
The relative log2 fold change of expression levels of the *dsbA* gene in the *D. solani* cells under stressful conditions analyzed via qPCR. The data correspond to the means ± standard deviation (SD) of three biological replicates with three technical repeats. The red horizontal lines denote a relative two-fold increase/decrease in the *dsbA* expression level. * indicates statistically significant (95% Confidence Interval) fold change in expression level according to the REST 2009 software.

To confirm that DsbA does not play a significant role in the protection of the *D. solani* cells against stress consequences, we examined the expression of the *dsbA* gene under selected stressful conditions. As shown in Figure 1, treatment with stressors generally did not lead to a significant induction of the *dsbA* transcription. However, we observed *dsbA* repression under several growth conditions, particularly in the exponentially growing cells in the presence of osmotica (both ionic and non-ionic) or in stationary-phase growing cells at 40 °C. Only three culture conditions, treatment of stationary-phase cells with sodium chloride (NaCl), hydrogen peroxide (H_2_O_2_) or acid pH, seemed to increase the *dsbA* mRNA content, but the differences were not statistically significant. Hence, DsbA does not seem to be a significant factor in the *D. solani* stress response.

### 2.2. The D. solani dsbA Mutant Is Impaired in Motility

**Figure 2:**
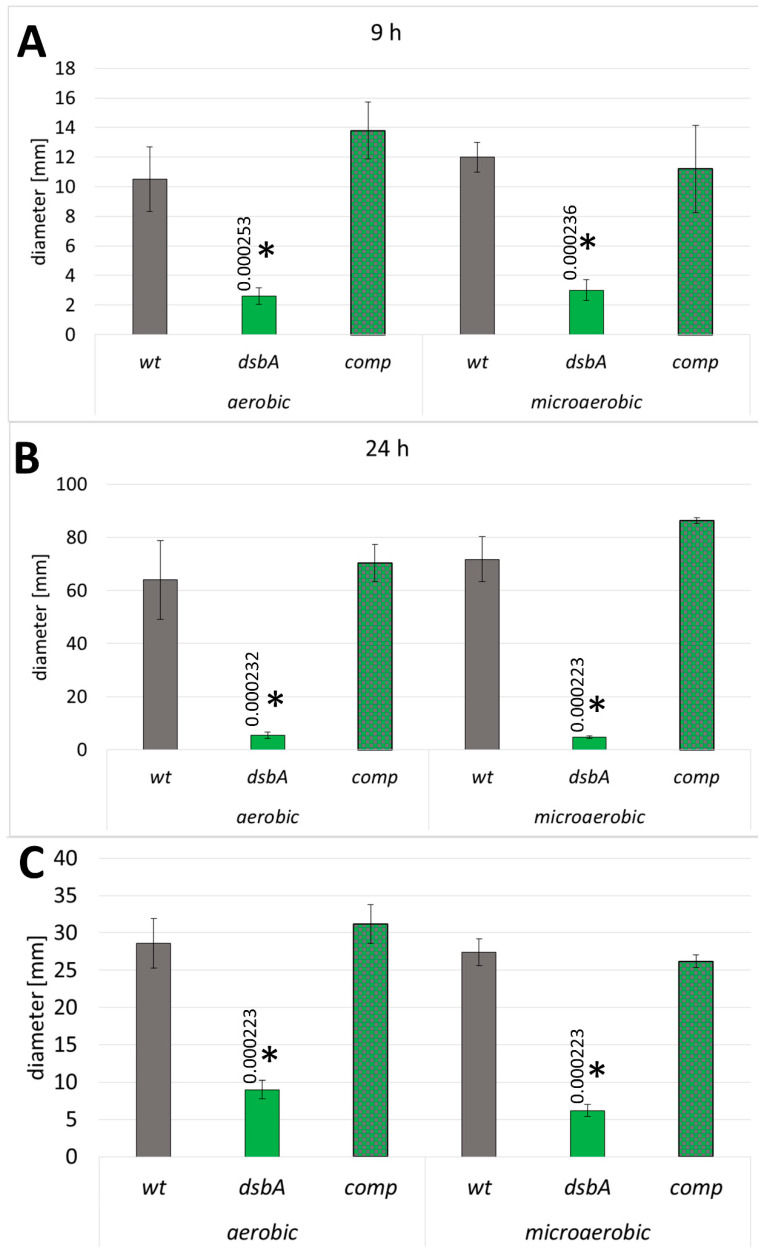
Effect of the *dsbA* knockout on the motility of *D. solani*. Mean values of swarming zones after 9 and 24 h of incubation (**A**,**B**) and swimming zones (**C**) are shown; wt—wild-type strain; *dsbA*—mutant strain TP02; comp—complemented strain TP03. The data represent values for five biological replicates. The statistical analysis of differences was determined by two-way analysis of variance (ANOVA) test followed by a post hoc Tukey test. The asterisks indicate statistical significance (*p* < 0.001); the numbers next to asterisks show the actual *p*-values.

We found that the *dsbA* mutant exhibited a dramatic reduction in swarming and swimming motility both under microaerobic and aerobic conditions. On swarming plates (0.5% agar), the migration of the mutant cells was hardly detectable when the wild-type bacteria migrated efficiently and formed large swarming growth zones after 24 h (Figure 2A,B and Figure S7A). Non-swarming *dsbA* cells grew as a confined colony in the center of the plate. Under prolonged incubation, the colony diameter of non-swarming cells may increase owing to the contribution of sliding motility [31], as observed on plates incubated for 24 h. The *dsbA* mutant failed to exhibit swimming motility in 0.3% agar (Figure 2C and Figure S7B). The expression of DsbA in the complemented strain restored both types of motilities (Figure 2 and Figure S7).

### 2.3. The dsbA Mutant Shows Impaired Activity of Secreted Virulence Factors

**Figure 3:**
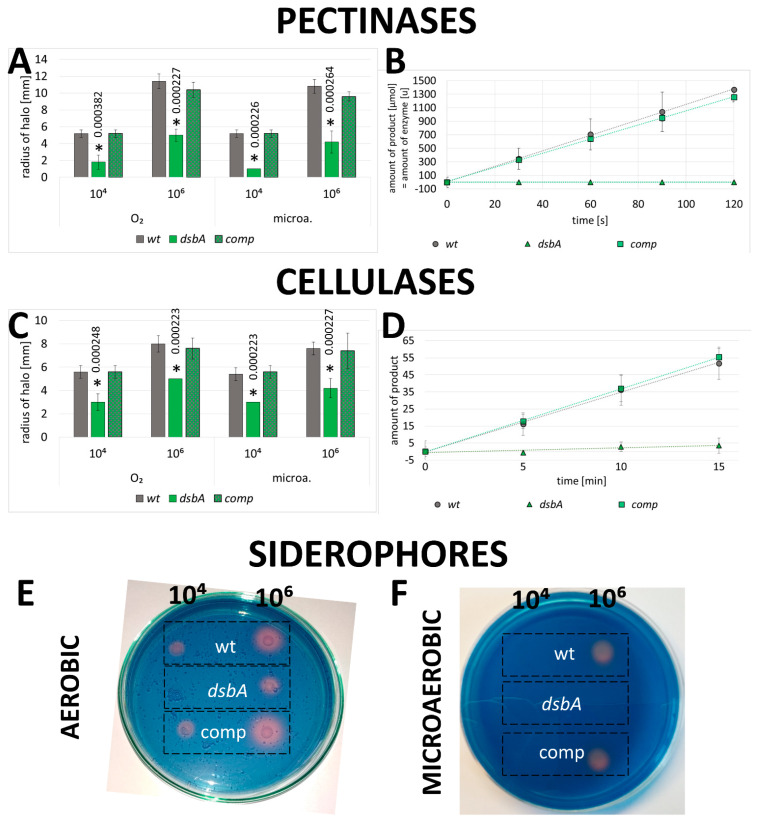
Impact of the *dsbA* knockout on the secreted virulence factors of *D. solani*: (**A**,**B**) pectinolytic activity, (**C**,**D**) cellulolytic activity and (**E**,**F**) siderophore secretion. Panels **A** and **C** show the mean values of the halo radii determined by plate assays. The statistical analysis of differences was determined by two-way analysis of variance (ANOVA) test followed by a post hoc Tukey test. The asterisks indicate statistical significance (*p* < 0.001); the numbers next to asterisks show the actual *p*-values. **B** and **D**—spectrophotometric determination of the enzymes’ activity in culture supernatants. Error bars represent SD from five experimental repetitions. Representative examples of Chromo-Azurol stained plates used to detect siderophore production are shown in **E**, **F**. In every assay, five independent replicates of each strain were analyzed. Abbreviations: wt—wild-type strain; *dsbA*—mutant strain TP02; comp—complemented strain TP03.

As a part of the phenotypic characterization of the *D. solani dsbA* mutants, we measured the activity of secreted PCWDEs (pectinases, cellulases and proteases) using plate assays. We found that bacteria lacking the functional *dsbA* gene were severely defective in the secretion of active pectinases into the growth medium. As demonstrated in Figure 3A and Figure S8A, the pectinolytic activity of the *dsbA* mutant was very low in plate assays. In contrast, the wt and complemented strains secreted comparable amounts of active enzymes. To further quantify the effect of DsbA on the secreted pectate lyase production, we used a quantitative spectrophotometric assay to measure pectinolytic activity in culture supernatants. While no pectate lyase activity was detected in the *D. solani dsbA* secretome, polygalacturonate was efficiently degraded in the presence of the control bacteria supernatants (Figure 3B).

Similarly, the *dsbA* knockout significantly affected the activity of secreted cellulases. The hydrolysis of the cellulase substrate was markedly less efficient in the *dsbA* mutant secretome than in the control bacteria secretomes (Figure 3C,D and Figure S8B).

In contrast, the proteolytic activity was not impaired by the *dsbA* knockout, as judged from the casein degradation zone diameters observed in aerobic or anaerobic growth conditions (Figure S9).

*D. solani* produces water-soluble siderophores that strongly bind ferric iron under iron-limiting conditions. We observed that siderophore secretion was significantly reduced in the *dsbA* mutant when compared to the control bacteria (Figure 3E,F).

### 2.4. DsbA Is Required to Establish the Infection in Potato Plants and to Cause Disease Symptoms

**Figure 4:**
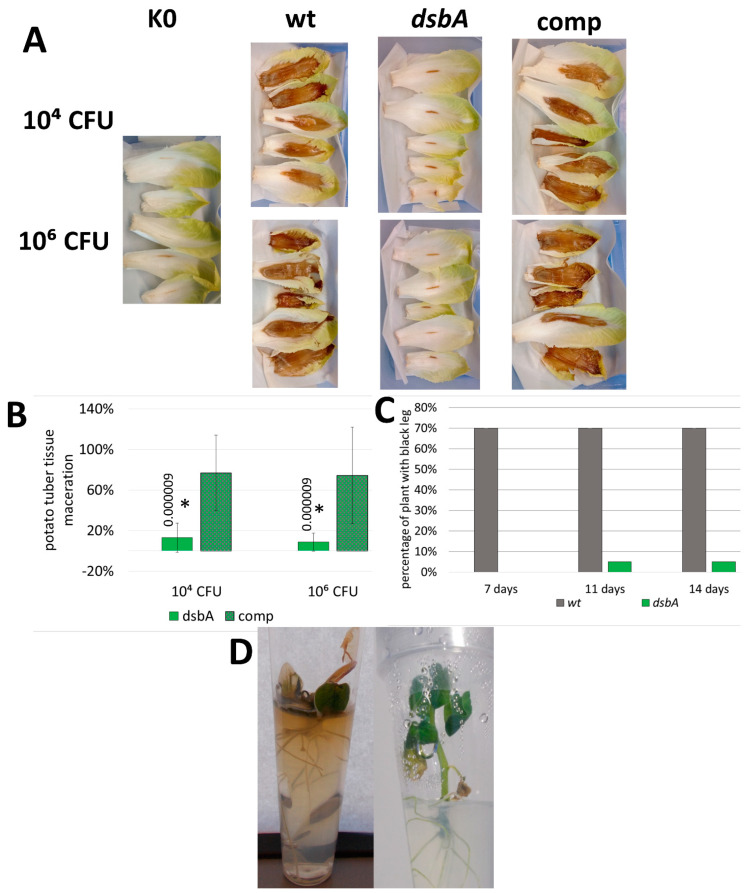
Influence of the *dsbA* mutation on the *D. solani* virulence. For the virulence tests, maceration of plant tissues was evaluated on isolated plant parts (**A**,**B**) and in planta infection assays were performed on whole plants (**C**,**D**). (**A**) Representative examples of chicory leaf infections. Five independent biological replicates of each strain and bacterial inoculum were analyzed. (**B**) Relative values of potato tuber maceration caused by the mutant TP02 (*dsbA*) and the complemented strain TP03 (comp) expressed as percentages of maceration caused by the wt *D. solani* cells. The weight of tissue macerated by wild-type bacteria was set at 100%. Per set of strains, nine individual tubers were inoculated in two independent experiments (*n* = 18). Statistical analysis of differences was determined by two-way analysis of variance (ANOVA) test followed by a post hoc Dunnett test. The result was considered significant at *p* < 0.05; the numbers next to asterisks show the actual *p*-values. (**C**) Percentage of the potato plants showing black leg symptoms in the pot assay. Five independently grown cultures of each bacterial strain were used in the assay. Each culture was used to infect 4 potato plants, giving 20 plants per strain (*n* = 20). Inoculated plants were visually inspected 7, 11 and 14 days post infection for wilting, typical blackleg symptoms and plant death. (**D**) Representative examples of the potato plantlets infected with *D. solani* wt (left) and *D. solani dsbA* (right).

To determine the effect of the *dsbA* mutation on *D. solani* virulence, we conducted various infectivity tests using potato (*Solanum tuberosum*) and chicory (*Cichorium intybus*) as host plants. In each test, a decreased ability of the *dsbA* mutant to infect plant tissues and develop disease symptoms was observed. First, the *D. solani dsbA* mutant did not cause the maceration of chicory leaves, contrary to the control strains. The efficiencies of tissue maceration caused by the wt and complemented strains were very similar, confirming that the avirulent phenotype of the *dsbA* mutant was indeed a result of a lack of the DsbA function (Figure 4A). Secondly, the *dsbA* mutant was inefficient in the maceration of whole potato tubers (Figure 4B and Figure S10). Using a potato tuber slice assay, the maceration level was dependent on the bacteria density used for inoculation. Tuber tissue rotting occurred when at least 10^4^ CFU of bacteria were used for inoculation. With that number of bacteria, the efficiency of rotting was clearly higher in the case of the control bacteria, while at 10^6^ CFU, a moderate maceration of tuber tissues was also visible in the case of the *dsbA* mutant (Figure S11). Good oxygen availability in the tuber slice assay can at least partially suppress the effects of the *dsbA* knockout. In the whole tuber assay, the oxygen access is much more limited. Whole tubers infected with the *dsbA* mutant showed minor rotting symptoms, and the weight of macerated tissue was approximately five-fold lower than that produced by the wt *D. solani* strain. Disease symptoms caused by the complemented strain were similar to those produced by the wt *D. solani* strain (Figure 4B). These data prove that the phenotype of the *D. solani dsbA* mutant directly results from the *dsbA* inactivation. Under microaerobic conditions, the presence of a functional *dsbA* gene is necessary for potato tuber tissue maceration.

Then, the ability of the *D. solani dsbA* mutant to infect the whole potato plants and develop disease symptoms was examined. In planta experiments were performed using two models: potted potato plants (cv. Vineta) and in vitro grown potato plantlets (cv. Kondor). The latter approach allowed for extended studies under strictly controlled and sterile conditions. It turned out that the *D. solani dsbA* mutant was unable to efficiently develop disease symptoms. After inoculation with the *dsbA* mutant, only one pot-grown potato plant (out of twenty) showed black leg symptoms (Figure 4C), while none of the in vitro plantlets were symptomatic (Figure 4D). In contrast, the majority of potato plants infected with the wt *D. solani* quickly developed disease symptoms (Figure 4C,D). In plants inoculated with sterile Ringer buffer, no disease symptoms were observed during the entire course of the experiments.

The use of culture tubes-grown plants allowed us to check in detail whether the mutants could penetrate the host, disseminate and colonize the plant tissues. We observed that the mutant cells were present in the upper parts of the plantlets (1 cm above the surface of the culture medium) (Figure S12). Two days post inoculation (dpi)*,* the numbers of *dsbA* or wt cells in the plantlet stems were comparable. At the later stages of infection (9th or 13th dpi), the CFU counts were higher for the control strain than for the *dsbA* mutant (although the changes were not statistically significant).

### 2.5. The Lack of a Functional dsbA Gene Causes Pronounced Changes in the Overall D. solani Cell Proteome

**Figure 5:**
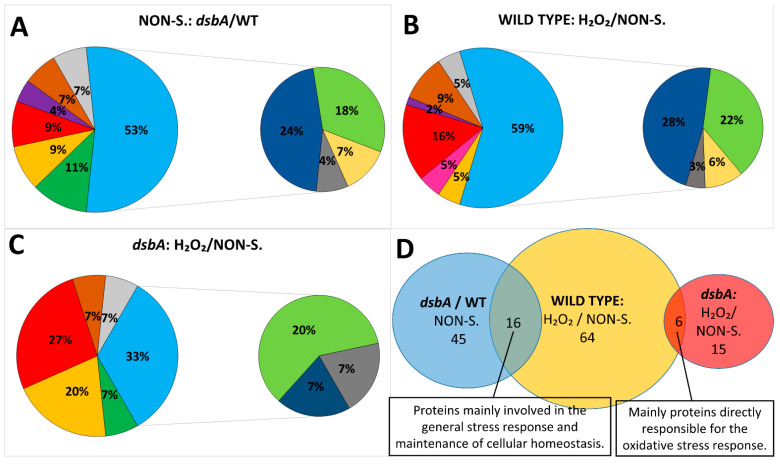
Graphic representations of proteomic data. (**A**–**C**) Functional categories and percentages of proteins whose levels were changed in the *D. solani* proteome due to (**A**) *dsbA* mutation, (**B**) oxidative stress or (**C**) in the *D. solani dsbA* mutant under oxidative stress. Areas in big circles: green: motility and chemotaxis; yellow: transport; red: stress response and protein quality control; pink: chaperones; purple: virulence and attachment; brown: lipoproteins, membrane and cell wall biogenesis; gray: others, unidentified; blue: metabolism. Areas in small circles: dark blue: general metabolism; green: protein synthesis; yellow: nucleotide and cofactors synthesis and homeostasis; gray: polyamine biosynthesis. (**D**) Diagram of unique and shared differentially expressed proteins identified in the proteome samples. Blue: proteins differentially expressed in the *dsbA* mutant compared to the parental wt *D. solani* cells, both grown under non-stressful conditions (NON-S.); yellow: proteins differentially expressed in the wt cells in the presence of the oxidant compared to the bacteria grown under non-stressful conditions; red: proteins differentially expressed in the *dsbA* mutant cells in the presence of the oxidant compared to cells grown under non-stressful conditions.

To gain more insights into the characteristics of the *D. solani dsbA* cells, the proteomes of the mutant and wt bacteria were compared by the means of Sequential Window Acquisition of All Theoretical Mass Spectra (SWATH-MS) analysis. This analysis identified a total of 607 proteins for which at least two peptides per protein were quantified (Table S1). Inactivation of the *dsbA* gene caused strong changes in the *D. solani* proteome. For further analysis, we narrowed the number of differentially expressed proteins by applying the following cut-off criteria: *p* < 0.05, as well as fold changes below 0.5 or above 2. As a result, we selected 45 proteins with significantly altered abundance. These proteins were grouped based on their functions: motility and chemotaxis, transport, stress response and protein quality control, virulence and attachment, primary metabolism, transcription and translation, membrane and cell wall biogenesis and finally, other or unknown functions (Table 1, Figure 5A).

First, we focused on proteins that could be related to the *D. solani* pathogenicity. Almost all of the mass spectra (MS) identified proteins linked to motility and chemotaxis, except CheV, showed decreased levels in the *D. solani dsbA* proteome compared to that of the wild-type bacteria. The protein showing the most reduced content (app. 100-fold) in the *dsbA* mutant was the flagellin FliC. The second protein involved in flagella structure, the flagellar hook protein FlgE, was also less abundant. Of the proteins involved in chemotaxis, two proteins showed significantly reduced levels, including CheA, a signal transduction kinase which participates in the transmission of signals from the chemoreceptors to the flagellar motors [32].

A lack of DsbA function led to the induction of proteins related to protein quality control (PQC) and to general stress responses. Among the periplasmic PQC components, only the DegP protease was markedly upregulated. The proteomic analysis suggested an increased abundance of some other periplasmic chaperones/folding helpers (SurA, FkpA); albeit their fold changes were lower than 2. Several cytoplasmic stress-related proteins were also upregulated, such as the glutathione synthetase GshB, the transcriptional regulator RcsB, the protein RecA required for the SOS response and probably an aldo/keto reductase (A0A2K8VWF4) (the fold changes were not statistically significant in the latter case).

The level of some proteins specifically involved in transport was either increased or decreased in the *dsbA* mutant. SitA, a metal transport protein, and PstS, a phosphate-binding protein, were present in larger quantities, while the concentrations of GltI, a glutamate/aspartate binding protein, and OppA, a periplasmic oligopeptide binding protein, were reduced in comparison to the control cells.

Enzymes related to primary metabolism constituted a large group of proteins with altered levels in the *dsbA* mutant. The ribulose-phosphate 3-epimerase RPE, an enzyme of the pentose phosphate pathway, was highly induced (app. 10-fold). Other abundant proteins included components of sugar metabolic pathways (SuhB and phosphoglucomutase), polyamine biosynthesis pathways (SpeA and SpeD), membrane and cell wall biogenesis (BamA, MurC and DcrB), amino-acid- and protein-synthesis-related pathways (HisD, TrpS, YxeP and elongation factors EF-Tu) and electron transfer (Cytochrome bo(3) ubiquinol oxidase, NAD(P)H-flavin reductase, NADH dehydrogenase and NAD-dependent malic enzyme). Some metabolism-related proteins were less abundant in the *dsbA* mutant cells, such as L-asparaginase (catalyzing the transformation of L-asparagine into L-aspartic acid and ammonia), a putative periplasmic phosphatase (A0A2K8W5K9), 2-isopropylmalate synthase, TypA (50S ribosomal subunit assembly factor) and BipA (a member of the ribosome-binding GTPase superfamily).

### 2.6. Exposure to Oxidative Stress Causes More Significant Changes in the Proteome of the wt D. solani Cells Than in That of the dsbA Mutant

As the *dsbA* mutation is related to disturbances in cellular redox balance, we also compared proteomes of bacteria treated with oxidative stress. Treatment with the oxidant H_2_O_2_ caused pronounced effects on the cellular proteome of *D. solani* IPO2222. The levels of 64 proteins were significantly altered: 51 proteins were upregulated, while 13 were down-regulated in comparison to protein levels found in bacterial cells grown under non-stressful conditions (Table 2, Figure 5B,C).

First of all, we observed a significant increase in the abundance of proteins directly engaged in cellular defense against reactive oxygen species (ROS) or in general stress response. These include ROS-neutralizing proteins (catalase KatG, superoxide dismutase, organic hydroperoxide resistance protein OhrB and alkyl hydroperoxide reductase), proteins providing reducing power (glutathione reductase and thioredoxin), proteins protecting DNA (Dps and universal stress protein E—UspE), protein quality control protease ClpP and envelope stress-induced periplasmic chaperone Spy. Additionally, an elevated level of enzymes participating in polyamine biosynthesis (SpeA, SpeE) can be considered as part of the stress response. Furthermore, a group of enzymes involved in membrane and cell wall biogenesis was upregulated. These include the outer membrane protein assembly factor BamD, the osmotically inducible lipoprotein E, L,D-transpeptidase, the UDP-N-acetylmuramate-L-alanine ligase and the UDP-N-acetylmuramoyl-tripeptide-D-alanyl-D-alanine ligase. The AccA protein, which provides the malonyl-CoA substrate for the biosynthesis of fatty acids [33], was also more abundant in H_2_O_2_-stressed cells.

The oxidative stress also caused changes in the cellular content of many primary metabolic enzymes. The ribulose-phosphate 3-epimerase RPE was the most strongly upregulated protein. Since this enzyme is particularly sensitive to oxidative damage [34], an increased synthesis is most probably necessary to maintain proper function of the pentose phosphate pathway.

We also observed that several pathways which are related to protein synthesis were upregulated. In particular, we detected increased levels of some proteins engaged in the translation process (TrpS, YfiA, EF-Tu and peptide chain release factor 3) and enzymes involved in the de novo amino acid biosynthesis (HisB, HisD, TrpD and sulfite reductase (NADPH) flavoprotein) and metabolism (*S*-adenosylmethionine synthase MetK). In contrast, the abundance of certain ribosomal proteins (L24, S10 and S20) decreased. Moreover, the levels of two proteins related to the transcription process were altered. The content of CspE, whose activity is important for antitermination and the decrease in transcription pausing in *E. coli* [35], was reduced, while the Exoribonuclease 2, involved in mRNA degradation [36], was upregulated, suggesting a downregulation of the transcription process in H_2_O_2_-stressed cells.

Interestingly, the *dsbA* mutant cells treated with H_2_O_2_ showed significantly less changes in their proteome compared to the wt cells. In fact, only 15 proteins showed altered abundance, and the changes were mainly related to proteins typically induced under oxidative stress conditions (KatG, superoxide dismutase, alkyl hydroperoxide reductase and Dps). Other elevated proteins, which were also found in the stressed wt cells, were SpeE and lipase (A0A2K8VYG6). Interestingly, we detected some proteins whose content was increased in the *dsbA* mutant but not in the parental wt *D. solani*. These include the protein export chaperone SecB, an outer membrane receptor involved in siderophore uptake (A0A2K8W4X3), RplL, the bifunctional polymyxin resistance protein ArnA and the cell division protein ZapB.

### 2.7. The Lack of the Functional dsbA Gene Affects the Secretion of Virulence Factors

As the function of the DsbA oxidase is particularly important to stabilize the structure of secreted proteins, we also compared the protein content of the extracellular compartment (secretome) of the wt and mutant strains. Our MS analysis identified 573 proteins which were present in the growth medium of the *D. solani* culture (Table S2). The secretome profile of the *dsbA* mutant was very different from that of the wt *D. solani* strain. First of all, we observed that the content of secreted virulence factors was drastically reduced (Table 3). This effect was particularly pronounced in the case of the PCWDEs: pectate lyases (PelA, PelC_1, PelC_2, PelE, PelL_1 and PelL_2), pectin lyase (Pnl), pectin esterase A (PemA), glucuronoxylanase (XynC), the protease PrtA and a neutral metalloproteinase. Another group of underrepresented proteins in the *dsbA* mutant secretome includes flagellum components: the flagellin FliC, the filament cap protein FliD, the hook-filament junction (FlgL and FlgK), the hook FlgE, the distal rod FlgG and the proximal rod (FlgF, FliE, FlgB, and FlgC).

**Figure 6:**
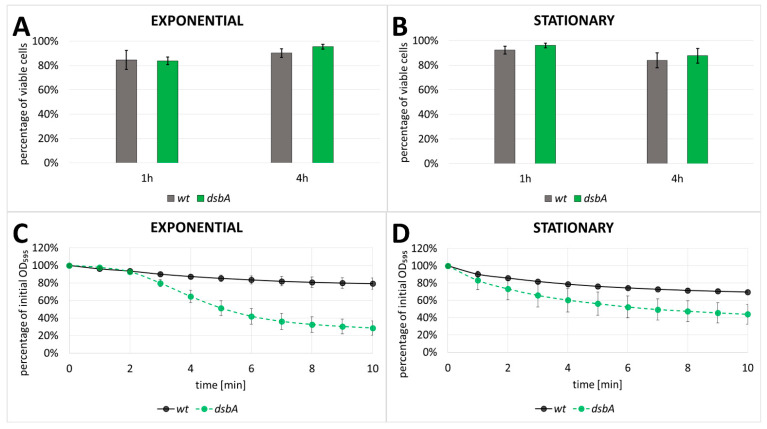
Analysis of membrane permeability using LIVE/DEAD fluorescence microscope assay (**A**,**B**) and sensitivity to SDS (**C**,**D**). The assays were conducted in exponential (**A**,**C**) and stationary (**B**,**D**) growth phases (wt—wild-type strain; *dsbA*—mutant strain, TP02). In LIVE/DEAD assay, data obtained from three independent biological replicates with at least three technical replicates per sample were analyzed. In the SDS sensitivity assay, the error bars represent SD from five independent biological replicates in two independent experiments (*n* = 10).

Since the levels of many cellular proteins were increased in the secretome fraction of the *dsbA* mutant, we deduced that the membrane integrity might have been weakened in the mutant cells, rendering them more prone to lysis. To verify this assumption, we tested the permeability of the membranes against compounds that have restricted access to intact cells. These include propidium iodide (PI) and sodium dodecyl sulfate (SDS). PI is a membrane-impermeable DNA-intercalating fluorescent dye which is regarded as the inner membrane permeability indicator [37]. SDS is an ionic detergent which disrupts the phospholipid bilayer, but due to the presence of lipopolysaccharide (LPS), Gram-negative bacteria are resistant to low concentrations of SDS [38]; therefore, SDS may serve as an indicator for outer membrane disturbance. We found that PI stained comparable numbers of the wt and mutant *D. solani* cells (Figure 6A,B), suggesting that the inner membrane integrity was not affected by the *dsbA* mutation. However, we observed a pronounced negative effect of SDS on the *dsbA* mutant. The *dsbA* cells were sensitive to the presence of 1% SDS, which induces frequent cell lysis (Figure 6C,D). In this condition, the wt cell lysis was only observed at a low frequency. Taken together, these results indicate that a lack of DsbA led to outer membrane alterations, while the inner membrane seemed to be unaffected.

## 3. Discussion

The DsbA homologs are oxidoreductases present in most bacterial species where they are responsible for introducing disulfide bonds in extracytoplasmic proteins. The majority of the *dsbA* mutants exhibit pleiotropic phenotypes due to the incorrect folding of numerous periplasmic and secreted proteins. As a result, the mutants show reduced fitness and attenuated virulence in the animal infection models due to improper folding/stability of several crucial virulence factors [8].

In this study, we explored the role of DsbA in the fitness and pathogenicity of a potato pathogen, *D. solani*. First of all, we found that the inactivation of *dsbA* in *D. solani* IPO2222 strongly reduced the bacterial virulence. Using both a natural host plant, potato, and a model host plant, chicory, we demonstrated that the *dsbA* mutant exhibited a markedly lower ability to macerate plant tissues than the wild-type strain IPO2222. Likewise, the *dsbA* mutant could not develop disease symptoms in whole potato plants grown in potting compost under phytochamber conditions. The observed lack of virulence can be due to several reasons: (i) the inability of the bacterial cells to penetrate the host, (ii) problems with enduring stressful conditions during host colonization, (iii) defects in growth and multiplication inside the host and finally, (iv) disturbances in the production and secretion of virulence factors during infection.

At the initial stages of infection, the capability to actively move towards a plant host, driven by the perception of appropriate chemical signals and the subsequent penetration of bacteria into the apoplast, are of particular importance [29]. Therefore, both motility and chemotaxis are major virulence determinants of SRP bacteria, including *D. solani*. As reported for the *dsbA* mutants of other bacterial pathogens (*E. coli, P. syringae* pv. Tomato, *P. fluorescens* and *P. atrosepticum*) [23,39,40,41], the *D. solani dsbA* mutant exhibited reduced swimming and swarming motility. Both types of movement require the presence of functional flagella, and as shown in *E. coli*, DsbA is required to introduce proper disulfide bonds in the P-ring protein (FlgI) of the flagella motor [39]. *Dickeya* sp. also contains an FlgI homolog with very well conserved pairs of cysteines (according to UniProtKB), also expected to require DsbA for stabilizing disulfide bonding. Therefore, the non-motile phenotype exhibited by the *D. solani dsbA* mutant could be due to the non-functional flagella motor component FlgI. Although our proteomic MS analysis did not detect the protein FlgI, the content of the other flagella components was strongly reduced in the *D. solani dsbA* mutant proteome and secretome. For efficient motility, bacteria need to perceive appropriate chemotactic signals. Thus, aberrances in chemotactic response negatively affect cell motility [42,43,44,45]. According to our proteome analysis, several proteins involved in chemotaxis were less abundant in the *dsbA* mutant. Hence, the substantial reduction in *dsbA* mutant cell motility resulted from both the deficiency of the flagella motor and decreased chemotactic response. Similar observations were reported in the case of *P. atrosepticum*, where 9 proteins related to flagella synthesis were present at a reduced level in the *dsbA* mutant secretome, and 25 genes encoding components of flagella and chemotactic response were downregulated in the *dsbA* mutant in comparison with the wild-type strain [23]. As flagellar gene expression occurs sequentially, according to the order of flagella assembly, blocking at the flagellar basal body level leads to the repression of late genes (e.g., *fliC* encoding flagellin). It was noticed that the *P. atrosepticum dsbA* mutation also causes partial repression of the early and middle flagella synthesis genes [23]. Our proteomic data indicate that the levels of all detected flagella proteins decreased in the *D. solani dsbA* mutant. One reason for this may be the envelope stress generated by the *dsbA* inactivation. In *S. enterica* serovar Typhimurium, the general envelope stress turns off the production of flagella [46]. Therefore, in the *D. solani dsbA* mutant, the synthesis of flagellar proteins may also be repressed by the envelope stress caused by the DsbA lack of function-dependent redox imbalance. This assumption agrees with our finding that the *D. solani dsbA* mutant contained an elevated level of the protein RcsB (app. 2-fold increase). RcsB is a transcriptional regulator that responds to envelope stress, and it is known to negatively regulate motility by directly repressing the operon *flhDC* encoding the master regulator of flagellar gene transcription [47,48,49].

Once inside the plant, the *D. solani* cells are challenged with several hostile factors, such as osmotic, oxidative and acidic stresses. We observed that the inactivation of *dsbA* in *D. solani* did not impair bacterial growth under neither physiological nor stressful conditions. This result was in agreement with a lack of induction of the *dsbA* expression in most stress-exposed *D. solani* cultures. Hence, it seems that the stress response in *D. solani* does not require elevated levels of the protein DsbA. The lack of growth defects due to the *dsbA* mutation in *D. solani* is not exceptional, as the *dsbA* mutants of several other bacterial species grow similarly to their parental strains under many tested conditions [50,51]. This phenomenon can be explained by a functional redundancy or overlapping activity among the extracytoplasmic folding factors [52,53]. The effects of the *dsbA* mutation become visible in the presence of additional mutations affecting protein quality control. For example, double inactivations of *dsbA* and *degP* (*htrA*) [54,55] or *dsbA* and *surA* [56] result in an increased sensitivity to several stresses. The genes *degP* and *surA* encode essential components of the protein quality-control system. DegP is a periplasmic protease/chaperone from the HtrA serine proteases family, responsible for removing aberrant proteins from the cellular envelope [57]. SurA serves as a general periplasmic chaperone for outer membrane proteins. Indeed, we found that the proteomes of the *D. solani dsbA* mutant contained increased concentrations of the HtrA homologs DegP (app. 3-fold increase ) and DegQ (app. 1.5-fold increase). A deficiency of DsbA also led to increased levels of various periplasmic chaperones and folding helpers: FkpA (1.6-fold increase), outer membrane protein assembly factor BamA (2.5-fold increase) and BamD (1.9-fold increase) (Table 1 and Table S1). The *p*-values of all of the above-mentioned proteins were significant.

During the later stages of plant infection, bacteria proliferate to reach a high density favorable to start the production of secreted virulence factors, such as PCWDEs, to rapidly destroy plant tissues and acquire nutrients reviewed in [29]. Several virulence factors of pathogenic bacteria require S-S bonds for their activity and stability. We found that activities of the crucial extracellular PCWDEs, pectinases and cellulases were strongly reduced in the *D. solani dsbA* mutant compared to the wt strain. According to this finding, the secretomes of the *dsbA* mutant contained negligible amounts of several pectate and pectin lyases and a low amount of pectinesterase A. Each of these enzymes contain at least two cysteine residues which are engaged in S-S bonding [58,59,60,61]. The lack of DsbA function can also affect the secretion system involved in export of PCWDEs. Some components of the *D. dadantii* Type 2 Secretion System (T2SS) have disulfide bonds which are essential for their correct folding and functioning [62]. Furthermore, genes encoding pectate lyases are under the negative control of the RcsB transcriptional regulator [63]. Therefore, an extremely low amount of secreted pectate lyases may also result from a decreased expression of their genes due to the elevated RcsB level observed in the *dsbA* mutant. The cellulase CelZ, which is responsible for the major endoglucanase activity in *D. dadantii* [64] and presumably also in *D. solani*, and a putative cellulase (A0A2K8VTS7) (Uniprot) were also strongly reduced in abundance. CelZ is known to contain S-S bonds [65], and the other enzyme also contains two cysteine residues. Interestingly, the secreted proteolytic activity was similar in the *dsbA* mutant and the parental *D. solani* strain. In the *P. atrosepticum dsbA* mutant, the enzymatic activities of all secreted enzymes were dramatically reduced: pectinase and cellulase activities were negligible and the protease activity was reduced to one-third that of the wild-type strain [23]. In the *D. solani* secretome, two proteases, PrtA and a neutral metalloproteinase, were approximately 2–3-fold less abundant (Table 1). Nevertheless, it is possible that the decreased amount of these two proteases was masked by the redundancy of proteases in the secretome. Although only a few proteases are known to participate in *D. solani* virulence, most of them can degrade milk proteins. As a result, the global proteolytic activity in the secretomes of both strains, *dsbA* and wt, was similar in the plate assays.

An interesting phenotype of the *D. solani dsbA* mutant, not previously observed in other bacteria, was a deficiency of secreted siderophores. *Dickeya* species secrete two types of siderophores: chrysobactin and achromobactin, which are necessary to acquire iron from an iron-poor environment in a plant host. Mutations that disable any of the siderophore-mediated iron transport prevent bacteria from spreading throughout the plant [66]. Hence, the secretion of siderophores is an important virulence determinant. Unfortunately, at the moment, we cannot explain the mechanism that leads to the impairment of the iron-chelating ability of the *dsbA* mutant, as none of the proteins directly involved in the siderophore synthesis or transport were detected in our proteome analysis. Nevertheless, we found that one of the proteins involved in iron uptake (A0A6N0B1L8, NCBI protein AYQ47458.1; Table 1), the periplasmic-binding protein SitA, was induced approximately by a two-fold factor in the *D. solani dsbA* mutant. SitA is homologous to YfeA of *Yersinia* or SitA of *Rhizobium meliloti* (according to the Paperblast database) [67,68]. YfeA, a component of the siderophore-independent, Yfe ABC permease, can bind manganese and iron ions. The *yfe* mutants show growth defects only under conditions of limited free iron availability, suggesting that iron acquisition is the main function of the Yfe system [67]. Our MS analysis indicates that in the *D. solani dsbA* mutant cells, the Yfe iron acquisition system is induced. The increased content of the Yfe system may compensate, at least partially, for a siderophore deficiency, and it may allow the mutant cells to colonize the host plant (albeit without developing disease symptoms).

The detailed analysis of the proteomes and secretomes of *D. solani dsbA* mutant and the parental strain enabled us to highlight the physiological effects of the DsbA deficiency. First of all, we noticed that in the *dsbA* mutant, several stress-response-related proteins were upregulated. This phenomenon concerned both envelope and cytoplasmic proteins. The first group can be attributed to the extracytoplasmic stress induced by the presence of misfolded proteins lacking proper disulfide bonds. Defects in the envelope protein folding and membrane alterations lead to the induction of the two major and partially overlapping envelope stress response systems: the Cpx two-component signal transduction pathway and the extracytoplasmic sigma factor σ^E^ (rpoE) dependent pathway. The induction of these systems leads to the stimulation of the synthesis of several extracytoplasmic protein quality control components, including chaperones and folding catalysts (e.g., Skp, spy, PpiA and PpiD), and the protease DegP [69,70,71]. Furthermore, the synthesis of components of the machineries involved in the transport and assembly of LPS and outer membrane proteins is also upregulated in an σ^E^ -dependent manner [72]. Indeed, our proteomic analysis revealed that the *dsbA* mutant contained elevated levels of Skp, spy, PpiA, SurA and BamA. Moreover, an increased abundance of the protein CpxP was detected in the *dsbA* mutant secretome. CpxP is a negative regulator associated with the sensor protein CpxA, but it also serves as a chaperone whose function is to present the unfolded proteins to the DegP protease. A high level of CpxP is a hallmark of Cpx pathway induction [73]. As mentioned previously, we observed an increased content of the transcriptional regulatory protein RcsB, a component of the Rcs system which senses damage or defects within the envelope and regulates the transcriptome to counteract stress. In particular, this system detects outer membrane damage, LPS synthesis defects, peptidoglycan perturbation and the mislocalization of lipoproteins [74]. The induction of the Rcs system has been reported in the *S. enterica dsbA* mutant [75]. In the *D. solani dsbA* mutant, we also found an increased content of some members of pathways maintaining envelope homeostasis. They include enzymes involved in: (I) the generation of energy and reducing power (components of the central carbon metabolism), (II) the synthesis of precursors needed for cell wall and membrane biogenesis (peptidoglycan and LPS synthesis pathways, the synthesis of CoA and fatty acid biosynthesis), and (III) general protein synthesis (amino acid biosynthesis pathways, ribosomal proteins and translation factors—EF-Tu and peptide chain release factor RF3). The central carbon metabolism plays essential roles in a cell by providing energy and precursors for many biosynthetic pathways. Under stress conditions, the central carbon metabolism and other metabolic pathways become altered to provide the metabolites necessary to overcome the stress effects reviewed in [76]. For example, we observed increased amounts of the pentose phosphate pathway steps generating the reductant NADPH and nucleotide precursors for DNA repair [77,78]. Ribulose-phosphate 3-epimerase, which participates in the nonoxidative phase of the pentose phosphate pathway, was the protein that showed the most increased level (>10-fold). The involvement of this enzyme in the stress response has not yet been reported in bacteria. However, in the yeast *Saccharomyces cerevisiae*, a lack of ribulose 5-phosphate epimerase resulted in an increased sensitivity to H_2_O_2_ [77]. Upregulation of the cell wall biogenesis enzymes may indicate issues with maintaining the integrity of the envelope (proper structure/function of the peptidoglycan and cell membranes). Increased expression of 25 genes involved in protein synthesis was also observed in the *P. atrosepticum dsbA* mutant, and this phenomenon has also been attributed to the stress response [23].

When analyzing the *D. solani* secretomes, we observed many proteins whose location were assigned to the periplasm or even to the cytoplasm. The pattern of most cellular proteins was generally comparable in both strains, indicating that the presence of these proteins in the growth medium was a result of cell lysis during the bacterial culture and possibly at the centrifugation step. However, many cytoplasmic and periplasmic proteins were enriched in the *dsbA* mutant secretome. This observation and the increased level of proteins involved in the cell wall and membrane biogenesis in the *D. solani dsbA* mutant proteome suggest that the cellular barriers do not work correctly in the mutant. This assumption was confirmed by the increased sensitivity of the *dsbA* mutant to low concentrations of SDS. A properly structured outer membrane provides protection against anionic detergents (e.g., SDS) [79], and permeabilization of the outer membrane can be manifested as increased susceptibility to the bacteriolytic action of detergents [80]. On the other hand, the *dsbA* mutant and wild-type strain of *D. solani* did not differ in PI entry; we thus concluded that the cytoplasmic membrane permeability was not affected by the lack of DsbA. It seems possible that the presence of not properly folded proteins due to the absence of DsbA leads to disturbances in the outer membrane and possibly to the peptidoglycan layer, making a cell more susceptible to lysis. There is also another explanation for the presence of cellular proteins in the growth medium. The removal of damaged proteins from the periplasm is one of the ways to deal with envelope protein folding stress. For example, protein leakage across the outer membrane was observed in the absence of the key protein quality control protease DegP (HtrA) [81]. In Gram-negative bacteria, *degP/htrA* mutants are characterized by an increased production of outer membrane vesicles, a phenomenon explained by the need to remove damaged proteins [82,83]. Therefore, the increased abundance of some periplasmic and outer membrane proteins in the *dsbA* mutant secretome may result from both protein leakage across damaged outer membrane and the elevated production of outer membrane vesicles.

The extracytoplasmic protein folding stress undoubtedly affects the protein homeostasis of the whole cell. The misfolding or deficiency of some membrane proteins can lead to disturbances in cell membrane functionality, for example, nutrient uptake. Indeed, the *dsbA* mutant proteome contained a lower content of some transport-related proteins, such as GltI (glutamate/aspartate periplasmic-binding protein), OppA (periplasmic oligopeptide-binding protein), inositol transport system sugar-binding protein (secretome) and hemin-binding protein (secretome). Moreover, as described above, the siderophore-dependent iron uptake system seems to be severely disturbed in the *D. solani dsbA* mutant. Dysfunctions of the envelope systems must have consequences for cytoplasmic homeostasis. Therefore, it was not surprising that the stress-related response was also induced in the cytoplasm. Several cytoplasmic proteins involved in stress response were significantly upregulated in the *D. solani dsbA* mutant, such as an organic hydroperoxide resistance protein of the Ohr/OsmC family, a glutathione synthetase and an aldo/keto reductase previously assigned as general stress protein 69. Moreover, we detected an increased content of the biosynthetic arginine decarboxylase SpeA and the S-adenosylmethionine decarboxylase SpeD, both involved in the biosynthesis of polyamines. Polyamines, e.g., putrescine and spermidine, play an essential role in stabilizing nucleic acids, increasing the efficiency of translation and providing resistance to environmental stress, including temperature changes, reactive oxygen species, osmotic pressure or the presence of toxic compounds [84].

Interestingly, the proteins mentioned above became induced in the wild-type *D. solani* strain by treatment with the oxidant H_2_O_2_. Several other proteins which were upregulated in the *dsbA* mutant were also induced in the wild-type strain under oxidative stress (Table 2, Figure 5D). Therefore, the impacts of the *dsbA* mutation in the cytoplasm seem to be similar to those of oxidative stress. This observation explains why oxidative stress caused slight changes in the *dsbA* mutant proteome. In fact, in the *D. solani dsbA* mutant treated with H_2_O_2_, mainly the proteins specific to oxidative stress response were upregulated (Table 2, Figure 5D). They included superoxide dismutase, DNA protection during starvation protein Dps, spermidine synthase, alkyl hydroperoxide reductase and catalase. The high level of an exported lipase observed in both strains after H_2_O_2_ treatment may reflect a necessity to modulate fatty acid composition in the oxidatively damaged membranes.

It is worth noting that the levels of the general cytoplasmic chaperones DnaK, ClpB, DnaJ and GroEL, were not significantly modified in the *dsbA* mutant, as well as in the wild-type strain under oxidative stress. Most probably, neither mutation nor the oxidant affected the structure of the cytoplasmic proteins significantly. This observation is in agreement with our previous finding, demonstrating that the *dnaK, dnaJ* and *groEL* genes were not induced by oxidative stress in *D. solani* [85].

## 4. Materials and Methods

### 4.1. Bacterial Strains and Growth Conditions

The wild-type *Dickeya* strains used in this study were *D. dadantii* 3937 [86] and *D. solani* IPO2222 [20]. The φ-EC2 generalized transducing phage was used for transduction of the *Dickeya* strains [87]. Bacterial strains and plasmids used in this study are listed in Table 4.

Bacteria were cultivated at 30 or 37 °C in minimal medium M63Y (0.1 M KH_2_PO_4_, 15 mM (NH_4_)_2_SO_4_, 9 μM FeSO_4_, 1 mM MgSO_4_, 1 mg L^−1^ vitamin B_1_ and 3 g L^−1^ glycerol, pH = 7.0) [92] in aerobic or microaerobic conditions, as described in [85]. When required, chloramphenicol (Cm) was added at a concentration of 12.5 µg mL^−1^. The oxidative stress was induced by 0.1 or 0.25 mM hydrogen peroxide (H_2_O_2_). For combined H_2_O_2_ and acid stress, the medium was acidified to pH = 5.0 by malic acid [93]. The osmotic stress was induced and analyzed as described in [85]. Briefly, ionic stress was induced by an increase in sodium chloride (NaCl) content to a concentration of 0.8 M in liquid medium or 0.1–0.3 M in solid medium. To induce osmotic non-ionic stress, sucrose was added to a final concentration of 0.32 M (liquid medium) or 0.3–0.6 M (solid medium). The thermal stress was induced by transferring the liquid bacterial cultures from 30 to 40 °C and subsequent 5 h incubation at elevated temperature. The assessments of cell cultivability and viability were performed as described in [85].

### 4.2. Construction of the D. solani dsbA Knockout Strain

The *D. solani dsbA* mutant was generated in two steps, according to [94]. In the first stage, the *dsbA* gene of the *D. dadantii* 3937 chromosome was disrupted by the insertion of a Cm resistance cassette. Briefly, the marker exchange plasmid containing the *D. dadantii dsbA* gene interrupted by the Cm cassette was constructed after amplification of the *dsbA* gene by PCR using the primers *Dd-dsbA-L* and *Dd-dsbA-R* (Table 5). The PCR product was cloned into the pGEM-7 vector (PROMEGA, Madison, WI, USA) into the SmaI restriction site, generating the plasmid pDF-dsbA. The Cm cassette used for the gene inactivation was amplified from the pCYC-184-cm plasmid using primers *Cm-Sawitzke-L* and *Cm-Sawitzke-R* modified from [90]. Then, the *dsbA* gene sequence was interrupted by introduction of the Cm cassette into the AfeI restriction site at one-third of the gene length to generate the marker-exchange plasmid pDF-dsbA::cm. The pDF-dsbA::cm plasmid was introduced to *D. dadantii* cells via electroporation. Marker exchange recombination was obtained after growth in a low-phosphate medium, as described by [94]. The presence of the Cm cassette within the *dsbA* gene in the *D. dadantii* chromosome was verified via PCR using the primers *Cm-Sawitzke-L* and *Cm-Sawitzke-R*.

Subsequently, the inactivated *dsbA* (*dsbA::cm^R^*) gene was transferred from *D. dadantii dsbA::cm^R^* (TP01 strain) into the *D. solani* IPO2222 chromosome by φ-EC2 phage transduction. First, a phage stock was prepared on the *D. dadantii dsbA::cm*. Then, the phage stock was used to infect *D. solani* IPO2222, and the mutants were selected on LB + Cm plates. Generally, five different Cm^R^ colonies were recovered from each transduction. The presence of the mutation in the *D. solani* genome was confirmed via PCR using the primers *Ds-dsbA-L* and *Ds-dsbA-R*.

### 4.3. Construction of the D. solani dsbA Complementation Strain

The *dsbA* gene from *D. dadantii* and a kanamycin (Kn) resistance cassette were cloned into the pGEM-7 vector generating the pDF-dsbA-kn plasmid. The *dsbA* gene was cloned into the SmaI restriction site. The Kn cassette was amplified from the pDOC-K plasmid [91] using the primers *Kan-L* and *Kan-R* and cloned into the PmlI restriction site, which is located 48 nucleotides downstream from the *dsbA* gene. Subsequently, the pDF-dsbA-kn plasmid was introduced via electroporation into the *D. dadantii* cells. The next steps were similar to the construction of the *D. solani dsbA::cm* mutant, except that selection was performed on LB + Kn plates. The reintroduction of the wild-type *dsbA* gene to its native locus in the *D. solani dsbA::cm^R^* genome was confirmed via PCR using the primers *Dd-dsbA-L* and *DddsbA-R*.

### 4.4. Quantitative Real-Time PCR (qPCR)

Bacterial cultures for gene expression analyses, subsequent RNA isolation, reverse transcription and quantitative real-time PCR were performed as described in [95]. The characteristics of the primers are shown in Table 6.

### 4.5. Virulence Determinants Assays

The motility of bacteria was evaluated under aerobic or microaerobic conditions. A swimming test was performed on the 0.3% agar MMA minimal medium (K_2_HPO_4_ 7g L^−1^, KH_2_PO_4_ 3 g L^−1^, MgSO_4_ × 7 H_2_O 0.1 g L^−1^, (NH_4_)_2_SO_4_ 1 g L^−1^ and 1 mM galactose as an attractant). Swarming was tested on the 0.5% agar TSA (Tryptic Soy Agar). In both tests, individual colonies of the wt or mutant strains were stab inoculated into the center of the motility plate and incubated right-side-up at 30 °C. The radius of outward expansion from the inoculation point was measured at 72 (swimming) or 9 and 24 h (swarming) post inoculation.

Activities of the secreted PCWDEs were determined using plate and spectrophotometric assays. The experiments on plates were performed essentially as described in [96]. In brief, 5 μL aliquots of bacterial suspensions of stationary grown cultures containing 10^4^ or 10^6^ CFU were spotted on appropriate indicatory plates and incubated for 72 h at 30 °C. Proteases were detected on a medium containing skim milk (12.5 g L^−1^) [97]. Pectinolytic activity was tested on M63Y medium with polygalacturonic acid (PGA), as described in [98]. The ability to secrete active cellulases was analyzed on M63Y agar plates supplemented with 2 g L^−1^ glycerol and 10 g L^−1^ carboxymethylcellulose (CMC), as described by [99].

The spectrophotometric assay of pectate lyase activity was based on [100,101]. The rates of increase in absorbance at λ = 235 nm (A_235_) due to the formation of unsaturated products from polygalacturonate [102] were measured every 30 s for 2 min. The enzyme activity (units) (micromoles of 4,5-unsaturated product released in one minute) was calculated using the formula:

$$enzyme\left[u\right]=\frac{∆\left|Abs_{235nm}\right|}{l\times \varepsilon_{235nm}}÷OD_{595nm}\times 10^{6}÷dillution;$$

$$l=1cm,\varepsilon_{235nm}=4600\frac{dm^{3}}{mol\times cm}$$

The cellulase activity spectrophotometric assay was conducted based on [101,103]. The cellulolytic activity was expressed as the amount of digested product of ostazin brilliant red cellulose using the following formula:

$$amount\;of\;product={∆}_{550nm}\times 100÷OD_{595nm}$$

The ${∆}_{550nm}$ value represents a difference between absorbance values of the test and control samples; OD_595_ is the optical density of cultures. The control sample was fresh growth medium with the ostazin brilliant red cellulose solution. Both spectrophotometric tests were performed using the U-5100 spectrophotometer (Hitachi, Tokyo, Japan).

To detect secreted siderophores, the Chrome-Azurol-S agar was used according to [104]. After incubation, the diameter of the orange halo around bacterial colonies was measured.

### 4.6. Infection Tests

The virulence of the *D. solani dsbA* mutant and the control strains (the parental wt strain and the complemented strain) was assessed on a model plant, chicory (*Cichorium intybus*), and the natural host, potato (*Solanum tuberosum*).

The chicory leaf virulence test was performed as previously described [105]. Briefly, each leaf was infected with 20 μL aliquots of bacterial solutions containing approximately 10^4^ or 10^6^ CFU per inoculation site. After incubation in a dew chamber for two days at 30 °C, the sizes of rotten tissue were evaluated and compared. At least five chicory leaves were used for each strain and each bacterial suspension.

The potato tubers (cv. Vineta) obtained locally in Gdansk, Poland were prepared and infected basically as described in [105]. For each tuber, the mass of tissue rotten caused by the wt *D. solani* strain was set at 100%, and a degree of maceration caused by the other strains was calculated as the percentage of the wt value. This approach enabled us to minimize the maceration differences due to the different properties of each tuber. The experiments were performed twice. Nine tubers were used for each biological replicate.

For the potato tuber slice test, the tubers of potatoes cv. Vineta were sterilized as described in [13] and sterile cut into approximately 1 cm thick slices. In the middle of each slice, a hole was made and 10 µL aliquots of suspensions of each strain containing 10^4^ or 10^6^ CFU were inoculated. As a negative control, 10 µL of sterile Ringer solution was injected. For disease development, the slices were incubated at 30 °C for 72 h in a humid box, then the diameters of rotting tissue around the wells were compared.

Statistical analysis of differences was determined by a two-way analysis of variance (ANOVA) test followed by a post hoc Dunnett test. A result was considered significant at *p* < 0.05.

The pot-grown potato plants were obtained from potato tubers cv. Vineta and subsequently cultivated as described in [95]. The plants were infected after root injury by soaking potting compost in each pot with 30 mL of bacterial suspensions containing 10^8^ CFU mL^−1^. Five independent repeats were used for the infection of twenty plants with each strain (*D. solani* wt or *dsbA* mutant). As a negative control, twenty plants were inoculated with sterile Ringer’s solution. On the 7th, 11th and 14th day post infection (dpi), symptoms were visually assessed for wilting, blackleg, stem desiccation and plant death, and the percentage of diseased plants was calculated.

The in vitro grown potato plantlets of cultivar Kondor were propagated on Murashige and Skoog (MS) medium [106] with 30 g L^−1^ sucrose and 7 g L^−1^ agar in culture tubes as previously described [107]. The plantlets were grown at 20–22 °C for 20 days prior to inoculation with bacteria. For inoculation, 10 μL aliquots of bacterial suspensions in water containing 10^5^ CFU mL^−1^ of *D. solani dsbA::cm* (three independently obtained isolates) or the parental *D. solani* IPO2222 (positive control) or sterile Ringer solution (negative control) were deposited at the interspace between the stem base and MS medium of each individual plant. Thirty-six individual plantlets were used per bacterial strain or negative control. The inoculated plantlets were grown at 20–22 °C under white fluorescent light with a 16 h photoperiod (white cool fluorescent light, Philips, TLD 58 W/84o, 30–35 μmol m^−2^ s^−1^). On the 2nd, 6th, 9th and 13th dpi, the plantlets were visually assessed for disease symptoms (wilting, typical blackleg, turbidity around the roots, stem desiccation and plant death), and the percentage of blackleg-diseased plants was calculated per time point. At the same time, 9 plants per strain were aseptically removed from the tubes and 1 cm long stem fragments were cut out from each plant approx. 1 cm above the stem base and separately suspended in 1 mL of 1/4-strength Ringer’s buffer (Merck, Darmstadt, Germany) supplemented with 0.02% diethyldithiocarbamic acid (Acros Organics, Geel, Belgium). To release bacteria from plant tissues, samples were grinded using sterile metal pestles, and serial dilutions were made in Ringer’s buffer and spotted onto LB agar plates to estimate the bacterial density (CFU per 1 g of plant tissue).

### 4.7. Cell Viability and Membrane Permeability Assays

The viability of cells was monitored by means of a LIVE/DEAD BacLight Bacterial Viability Kit (Thermo Fisher Scientific, Eugene, OR, USA), as described in [108]. Depending on the growth phase and culture density, 100–400 µL aliquots of bacterial cultures were centrifuged, washed and suspended in 100 µL of Ringer’s buffer. A freshly prepared mixture of fluorescent dyes (SYTO 9 and PI) was added to 10 µL portions of bacterial suspensions, according to the manufacturer’s instructions. Next, bacteria were pipetted onto glass slides, and pictures were taken after 8 min of incubation using a fluorescence microscope (AXIO Scop, Carl Zeiss, Jena, Germany). Data obtained from at least three experiments were analyzed with IMAGEJ software (http://imagej.nih.gov/ij/ accessed date: the 18 October 2017; Research Services Branch, National Institute of Mental Health, Bethesda, MD, USA.).

The permeability of the outer membrane was assessed by a degree of cell lysis in the presence of sodium dodecyl sulphate (SDS), determined by a decrease in the optical density (OD) of the cell culture. The method was partially based on [79,109]. The bacterial cultures were centrifuged, washed twice, and suspended in 1 mL of buffer PBS to OD in the range of 0.642–1.096 or 0.417–0.610 in the case of the exponential or stationary growth phase, respectively. Then, 900 µL aliquots of bacterial suspensions were mixed with 100 µL of 10% SDS (final concentration: 1%), and the optical density was measured at one-minute intervals for 10 min.

### 4.8. Mass Spectrometry

#### 4.8.1. Sample Preparation for Mass Spectrometry

Five independently inoculated cultures of each *D. solani* strain (wt or *dsbA*) were grown overnight in the minimal medium M63Y at 30 °C with aeration. Then, the OD of the cultures was normalized to 0.7, and the bacterial suspensions were inoculated 1:50 into fresh medium. Bacteria were further grown with shaking (180 rpm) for 16 h under microaerobic conditions. To induce oxidative stress, H_2_O_2_ was added to a final concentration of 0.25 mM, and the cultures were incubated for 30 min.

For the whole-cell proteome analysis, 14 mL of each culture was centrifuged (5000× *g*, 10 min), the cells were suspended in the lysis solution (4% SDS, 100 mM Tris/HCl pH 7.6, 0.1 M DTT) and incubated 9 min at 96–98 °C. Suspensions were cooled on ice for 10 min and stored at −80 °C.

To isolate the secreted protein fraction (secretome), 45 mL of each bacterial culture was centrifuged for 10 min at 5000× *g*. The supernatant, containing secreted proteins, was collected and filtered using syringe filters (PVDF membrane, 0.22 µm, Bionovo, Legnica, Poland) to remove the remaining bacterial cells. EDTA was added to a final concentration of 1.0 mM and incubated for 5 min on ice. Then, the supernatant volumes were reduced to 2 mL via centrifugation (6000× *g*, 4 °C) using VIVASPIN 20 concentrators (Membrane 3000 MWCO, Sartorius, Stonehouse, UK). The secretome samples were supplemented with protease inhibitors (Roche, Basel, Switzerland) and were further concentrated to a volume of 1 mL. The concentrated protein solutions (approx. 1 mL) were mixed with 9 mL of 99% cold ethanol and centrifuged (30,882× *g*, 4 °C, 30 min). The precipitates were washed in 2 mL of ethanol, centrifuged (30,882× *g*, 4 °C, 10 min) and dried at −20 °C overnight. Next, the secretome precipitates were dissolved in the lysis solution and stored at −80 °C.

#### 4.8.2. Protein Digestion

Proteins were digested by trypsin in a standard FASP (Filter-Aided Sample Preparation) procedure [110]. Briefly, 100 µg of protein was placed in a Microcon with a 10 kDa cutoff membrane (Merck-Millipore, Darmstadt, Germany) and washed multiple times with urea-containing buffer (8 M urea, 100 mM Tris-HCl, pH 8.5) via centrifugation at 10,000× *g*. Next, the cysteine residues were alkylated using the 55 mM iodoacetamide solution in the urea buffer with a 20 min incubation period in darkness at room temperature. The excess iodoacetamide was washed away with subsequent centrifugations with the urea buffer followed by the digestion buffer (50 mM Tris-HCl, pH 8.5). Filters were placed in new tubes, and samples were incubated with trypsin (1:50 enzyme-to-substrate weight ratio) overnight at 37 °C. After incubation, proteolytic peptides were eluted via centrifugation at 10,000× *g*. Samples were then desalted using a STAGE Tips procedure [111] on in-house-prepared tips containing Empore C18 resin (3M). Tips were prepared via subsequent centrifugations with methanol, 60% acetonitrile and 1% acetic acid in water and 1% acetic acid in water. Then, 10 µg of peptide was placed in each tip, and tips were washed with 1% acetic acid in water before the final elution with 60% acetonitrile and 1% acetic acid in water to new tubes. Samples were concentrated to 40 µL in a SpeedVac before mass spectrometry measurements.

#### 4.8.3. Liquid Chromatography and Mass Spectrometry in Data-Dependent Acquisition Mode

LC-MS/MS measurements were conducted in a positive ion mode on a Triple TOF 5600+ mass spectrometer (SCIEX, Framingham, MA, USA) coupled with an Ekspert MicroLC 200 Plus System (Eksigent, Redwood City, CA, USA). The instrument was operated by Analyst TF 1.7.1 software (SCIEX, Framingham, MA, USA). LC separations were carried out on the ChromXP C18CL column (3 µm, 120 Å, 150 × 0.3 mm, Eksigent, Framingham, MA, USA) in 60 min gradients of 3.5–20% buffer B or 7–35% buffer B for proteome and secretome analysis, respectively (buffer A: 0.1% formic acid in water, buffer B: 0.1% formic acid in acetonitrile). MS runs of whole-proteome samples in data-dependent analysis (DDA) mode consisted of a TOF scan in the *m/z* range of 400–1200 Da in 50 ms and a Product ion scan in the m/z range of 100–1800 m/z in 40 ms, resulting in a cycle time of 0.9 s. DDA analysis of secretome samples consisted of a TOF scan in the m/z range of 400–1200 Da in 100 ms and a Product ion scan in the m/z range of 100–1800 m/z in 50 ms, resulting in a cycle time of 1.15 s. Precursor ions were fragmented using collision-induced dissociation (CID). All samples were analyzed once for the purpose of spectral library generation.

#### 4.8.4. SWATH Mass Spectrometry Experiments

MS analysis in data-independent acquisition (DIA) mode was conducted with the SWATH-MS method [112] focused on high sensitivity. MS methods were adjusted separately for the whole proteome and secretome samples. In both cases, the m/z range of 400–1200 Da was covered by 25 overlapping windows of variable width constructed in the SWATHTuner software [113] with a focus on equalized ion frequency. All SWATH-MS measurements comprised a TOF survey scan in the m/z range of 400–1200 Da in 50 ms and Product Ion scans in the m/z range of 100–1800 Da in 40 ms, resulting in a cycle time of 1.1 s. All samples were measured in triplicate.

#### 4.8.5. Data Analysis

All data analysis steps were performed separately for the whole proteome and secretome samples. A database search was conducted in ProteinPilot 5.0.2 software (SCIEX, Framingham, MA, USA) against the UniprotKB *D. solani* D s0432-1 database (version from 19.10.2020) with an automated false discovery rate analysis. Only proteins identified at 1% FDR were considered valid identifications. SWATH-MS data were analyzed in a similar manner as described before [114]. Database search results were imported to the PeakView 2.2 software (SCIEX, Framingham, MA, USA) to construct spectral libraries with the exclusion of shared peptides. SWATH-MS measurement files were uploaded into software and analyzed using relevant libraries. The results were exported to MarkerView software (SCIEX, Framingham, MA, USA) and normalized using the Total Area Sums (TAS) approach. T-tests between studied groups were performed, and the protein concentration changes were considered to be statistically significant if the *p*-value was lower than 0.05 and the fold change was lower than 0.5 or greater than 2.

The mass spectrometry proteomics data were deposited to the ProteomeXchange Consortium via the PRIDE [115] partner repository with the dataset identifier PXD028047.

## 5. Conclusions

**Figure 7:**
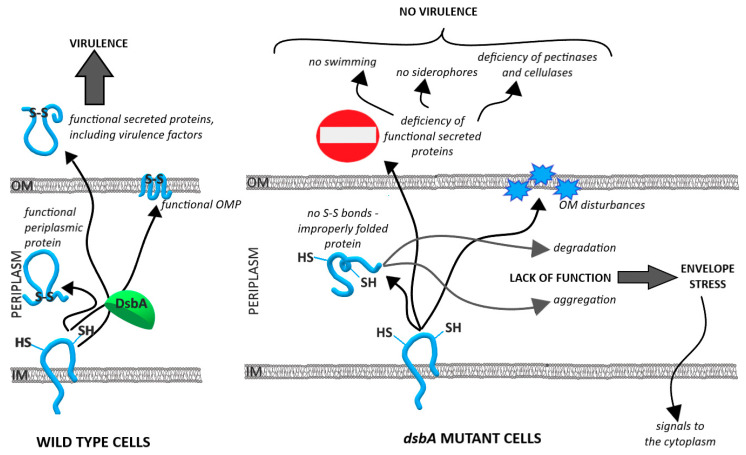
Model depicting the functioning of the *D. solani* cell in the presence and absence of the oxidoreductase DsbA. The properly functioning DsbA enzyme maintains the homeostasis of the cell envelope and ensures production of active virulence factors (left panel). The lack of DsbA disrupts the cell envelope homeostasis and causes an avirulent phenotype (right panel).

1. The lack of a functional *dsbA* gene resulted in the loss of virulence of *D. solani* IPO2222. Consequently, the mutant was unable to develop disease symptoms in the natural host, the potato plant.
2. The *dsbA* mutant was non-motile and deficient in the secretion of most extracellular virulence factors (pectinases, cellulases and siderophores).
3. The lack of the DsbA protein led to multifaceted effects in the *D. solani* cells, with pronounced changes observed in the cellular proteome and secretome.
4. The proteomic analysis suggested the induction of the extracytoplasmic and cytoplasmic stress responses in the *dsbA* mutant cells, that could lead to repression of biosynthesis pathways involved in virulence (flagella and PCWDE). A graphical summary of the conclusions is shown in Figure 7.

To our knowledge, our work provides the first comprehensive study regarding the importance of the DsbA function in the processes related to *D. solani* pathogenicity, which are based on detailed proteomic analyses and phenotypic and virulence assays.

## Figures and Tables

**Table 1:** Summary of proteins whose level depends on DsbA activity.

UniProt ID	Name	*p*-Value	Fold Change	Description	Number of Cys	Predicted Localization
MOTILITY AND CHEMOTAXIS						
A0A2K8VVH9 A0A6N0AT44	CheA	0.0023	*0.4227*	Signal transduction histidine kinase CheA	3	C
A0A6N0AAK8	CheV	0.0062	2.1167	Chemotaxis protein	1	C
A0A6N0AH46	CheW	0.0269	*0.4451*	Chemotaxis protein	0	C
A0A6N0B2V1	FlgE	0.0085	*0.3043*	Flagellar hook protein	0	EX
A0A6N0AG83	FliC	<0.0001	*0.0080*	Flagellin 1	0	EX
TRANSPORT						
A0A6N0AQD6	GltI	0.0010	*0.2706*	Glutamate/aspartate periplasmic-binding protein	2	EX
A0A6N0B4D0	OppA	0.0234	*0.3684*	Periplasmic oligopeptide-binding protein	0	EX
A0A6N0AX80	PstS	0.0214	2.6510	Phosphate-binding protein	0	EX
A0A2K8W558 A0A6N0B1L8	SitA	0.0025	2.6286	Manganese ABC transporter, periplasmic-binding protein	0	EX
STRESS RESPONSE AND PROTEIN QUALITY CONTROL						
A0A6N0AF95	DegP	0.0162	2.9982	Periplasmic serine endoprotease	2	EX
A0A6N0AY12	GroES	0.0004	*0.2959*	10 kDa chaperonin	0	C
A0A6N0BH32	GshB	0.0043	2.7279	Glutathione synthetase	4	C
A0A6N0ARS9	RecA	<0.0001	2.5880	Recombinase A	4	C
A0A6N0ABQ5	RcsB	0.0274	2.1452	Transcriptional regulatory protein	0	C
VIRULENCE AND ATTACHMENT						
A0A6N0ADK4	OmpW	0.0465	*0.4662*	Outer membrane protein W	0	EX
A0A2K8W5L9 A0A6N0BBS5	OmpX	0.0001	2.0530	Attachment invasion locus protein precursor	0	EX
PRIMARY METABOLISM						
A0A6N0AP75	MaeA	0.0202	3.0996	NAD-dependent malic enzyme	8	C
A0A2K8VWM7	SuhB	0.0004	5.5655	Inositol-1-monophosphatase	3	C
A0A6N0AXG4	AccA	0.0005	2.2560	Acetyl-coenzyme A carboxylase carboxyl transferase subunit alpha	4	C
A0A2K8W5K9 A0A6N0B1Z5		0.0059	*0.3826*	acidPPc domain-containing protein	3	EX
A0A6N0AKV1		0.0452	2.8777	Cytochrome bo(3) ubiquinol oxidase subunit 1	4	EX
A0A2K8VUJ2 A0A6N0AFZ5		0.0009	3.6718	2-dehydropantoate 2-reductase	5	C
A0A6N0AZD1		0.0260	*0.1796*	L-asparaginase	0	C
A0A2K8W0U8 A0A6N0AIW6		0.0472	*0.3564*	NADH dehydrogenase	6	C
A0A6N0AL19		0.0108	2.1397	NAD(P)H-flavin reductase	2	C
A0A6N0AAE7		0.0089	6.8300	Phosphoglucomutase	5	C
A0A6N0B8R8		0.0279	10.8132	Ribulose-phosphate 3-epimerase	1	C
→ protein synthesis						
A0A6N0B0C3	HisD	0.0057	2.5386	Histidinol dehydrogenase, histidine biosynthesis	7	C
A0A6N0AIR2	LeuA	0.0357	*0.4026*	2-isopropylmalate synthase	6	C
A0A6N0AQ52	L32 (RpmF)	0.0294	*0.4699*	50S ribosomal protein L32	0	C
A0A6N0AJL8	TrpS	0.0024	2.1145	Tryptophan-tRNA ligase	4	C
A0A6N0AWV6	TypA (BipA)	0.0012	*0.4929*	GTP-binding protein TypA/BipA (ribosome biogenesis)	5	C
A0A2K8VZB6 A0A6N0AI45	YxeP	0.0013	3.3600	N-acetyl-L,L-diaminopimelate deacetylase-like protein (Leu biosynthesis)	2	C
A0A6N0BB83		0.0012	3.9494	Elongation factor Tu	3	C
A0A6N0AJ97		0.0051	3.9942	Elongation factor Tu	3	C
→ nucleic acids AND cofactors synthesis and homeostasis						
A0A6N0BGD6	PurF	0.0098	*0.4078*	Amidophosphoribosyltransferase	6	C
A0A2K8VZA5 A0A6N0AL00		0.0002	2.1056	Adenylosuccinate lyase (*de novo* purine synthesis)	2	C
→ polyamine biosynthesis						
A0A6N0ASX1	SpeA	0.0010	3.1524	Biosynthetic arginine decarboxylase	8	EX
A0A6N0AWG0	SpeD	0.0253	2.0829	S-adenosylmethionine decarboxylase proenzyme	6	C
LIPOPROTEINS, MEMBRANE AND CELL WALL BIOGENESIS						
A0A6N0BB31	BamA	0.0019	2.5034	Outer membrane protein assembly factor	2	EX
A0A2K8VZQ3 A0A6N0AK79	DcrB	0.0126	2.3492	Periplasmic bacteriophage sensitivity protein DcrB	1	EX
A0A6N0BHR4	MurC	0.0060	2.9827	UDP-N-acetylmuramate-L-alanine ligase	2	C
OTHERS, UNIDENTIFIED						
A0A2K8W438 A0A6N0APE9	VirK	0.0480	*0.1451*	VirK protein (*D. dadantii* YbjX homologue)	2	EX
A0A2K8VTQ1 A0A6N0AFA2		0.0001	*0.3205*	CaMKII-AD, RNS: SgcJ/EcaC family oxidoreductase	2	EX
A0A6N0AEH1		0.0233	*0.4976*	Uncharacterized protein	0	N

UniProtID in gray are the archival numbers. The fold change values of downregulated proteins are in italics; number of cysteine residues in a protein (Number of. Cys); predicted protein localization: the cytoplasm (C), the extracytoplasmic space (EX), not known (N).

**Table 2:** The influence of oxidative stress on the proteomes of the *D. solani* wild-type strain and the derived *dsbA* mutant.

UniProt ID	Name	*p*-Value	Fold Change	*p*-Value	Fold Change	Description	No. of Cys	Pred. Local.
		**WILD TYPE**		***dsbA* MUTANT**				
MOTILITY AND CHEMOTAXIS								
A0A2K8W3X3 A0A6N0ABX5				0.0272	*0.3321*	Methyl-accepting chemotaxis protein I, serine chemoreceptor protein	3	EX
TRANSPORT								
A0A6N0B6V8	OmpF	<0.0001	*0.2333*			Outer membrane protein F	0	EX
A0A6N0AX80	PstS	0.0013	4.4512			Phosphate-binding protein	0	EX
A0A6N0A6N3	SecB			0.0139	2.2809	Protein-export protein SecB	4	C
A0A2K8VZD5 A0A6N0B8Y3				0.0453	*0.4820*	ABC-type polar amino acid transport system, ATPase component	1	EX
A0A6N0AZI4		0.0145	*0.4237*			Putative Fe(2+)-trafficking protein	1	EX
A0A2K8W4X3 A0A6N0AAN4				0.0138	2.0647	Outer membrane receptor protein, involved in siderophore uptake	0	EX
CHAPERONES								
A0A6N0B884	IbpB	<0.0001	*0.3876*			Small heat shock protein	0	C
A0A6N0BK24	SlyD	0.0053	*0.4416*			FKBP-type peptidyl-prolyl cis-trans isomerase	5	C
A0A2K8VY47	SurA	0.0098	2.0860			Chaperone	0	EX
STRESS RESPONSE AND PROTEIN QUALITY CONTROL								
A0A6N0AZJ6	ClpP	0.0003	2.0636			ATP-dependent Clp protease proteolytic subunit	2	C
A0A6N0AN16	Dps	0.0010	6.5526	<0.0001	5.6274	DNA protection during starvation protein	0	C
A0A6N0AYR4	KatG	0.0017	2.0880	0.0014	2.3612	Catalase-peroxidase	1	C
A0A6N0AGM0	OhrB	0.0039	6.8570			Organic hydroperoxide resistance protein	2	EX
A0A6N0AY64	Spy	0.0309	2.9841			Spheroplast protein Y	0	EX
A0A6N0ADU5	UspE	0.0320	*0.4192*			Universal stress protein E	4	C
A0A6N0A9Y2		<0.0001	4.7646	<0.0001	6.3572	Alkyl hydroperoxide reductase subunit C	2	C
A0A6N0BH32		0.0012	3.0958			Glutathione synthetase	4	C
A0A6N0A6E0		<0.0001	3.0858	0.0034	2.0317	Superoxide dismutase	1	C
A0A6N0BD39	EC-YbbN	0.0067	2.1508			Thioredoxin	2	C
VIRULENCE AND ATTACHMENT								
A0A2K8VV27 A0A6N0ARC4		0.0076	2.4047			Endo-1,4-beta-xylanase A, cellulolythic enzyme	2	EX
METABOLISM								
A0A6N0AXG4	AccA	0.0011	2.1010			Acetyl-coenzyme A carboxylase carboxyl transferase subunit alpha	4	C
A0A6N0AKX6	CoaBC	0.0380	2.6397			Coenzyme A biosynthesis bifunctional protein	6	C
A0A6N0AEA5	FabA	0.0124	2.9558			3-hydroxydecanoyl-[acyl-carrier-protein] dehydratase	2	C
A0A6N0AP75	MaeA	0.0379	2.4525			NAD-dependent malic enzyme	8	C
A0A6N0B091	MenB	0.0002	2.0532			1,4-Dihydroxy-2-naphthoyl-CoA synthase	8	C
A0A6N0BMC3	MetK	0.0005	2.2850			S-adenosyl-methionine synthase	4	C
A0A6N0B5I8	PanB	0.0029	*0.4842*			3-methyl-2-oxobutanoate hydroxymethyl-transferase	5	C
A0A6N0AI34	Ppa	0.0001	*0.2987*			Inorganic pyrophosphatase	2	C
A0A6N0B8R8	Rpe	<0.0001	9.3293			Ribulose-phosphate 3-epimerase	1	C
A0A2K8VWM7	SuhB	0.0425	2.0263			Inositol-1-monophosphatase	3	C
A0A2K8VW53 A0A6N0ARP4	YfdZ	0.0291	2.4133			Putative PLP-dependent aminotransferase YfdZ	4	C
A0A2K8VWF4 A0A6N0B632	YhdN_3	0.0010	3.6295			Oxidoreductase	0	C
A0A2K8VUJ2 A0A6N0AFZ5		<0.0001	3.3844			2-dehydropantoate 2-reductase	5	C
A0A6N0BJD2		0.0027	*0.1940*			4-hydroxy-3-methylbut-2-en-1-yl diphosphate synthase	5	C
A0A2K8VYG6 A0A6N0AT13		<0.0001	7.0231	0.0042	7.5702	Exported lipase	1	EX
A0A2K8VZB6 A0A6N0AI45		0.0010	3.3037			N-acetyl-L,L-diaminopimelate deacetylase-like protein	2	C
A0A6N0B1V3		0.0312	*0.4177*			NADH:flavin oxidoreductase	1	C
A0A6N0AAE7		0.0275	2.5083			Phosphoglucomutase	5	C
→ Protein synthesis								
A0A6N0BHG2	HisB	0.0023	2.5244			Histidine biosynthesis bifunctional protein	6	C
A0A6N0B0C3	HisD	0.0025	3.1096			Histidinol dehydrogenase, histidine biosynthesis	7	C
A0A6N0BKQ0	LeuC			0.0003	*0.4741*	3-isopropylmalate dehydratase large subunit 1, L-leucine biosynthesis	7	C
A0A6N0B5Q4	LeuD			0.0013	*0.4868*	3-isopropylmalate dehydratase small subunit 1, L-leucine biosynthesis	2	C
A0A6N0AIZ0	RplL			<0.0001	2.7155	50S ribosomal protein L7/L12	0	C
A0A6N0BJY9	RplX	<0.0001	*0.4967*			50S ribosomal protein L24	0	C
A0A2K8VYU2 A0A6N0B5N4	RpsJ	0.0005	*0.4470*			30S ribosomal protein S10	0	C
A0A6N0BIP4	RpsT	0.0048	*0.4073*			30S ribosomal protein S20	0	C
A0A2K8VTM5 A0A6N0BHV7	TrpD	0.0020	3.6390			Anthranilate phosphoribosyl-transferase	2	C
A0A6N0AJL8	TrpS	0.0003	2.2887			Tryptophan-tRNA ligase	4	C
A0A6N0BHM5	YfiA	<0.0001	2.0523			Ribosome-associated inhibitor A	1	C
A0A6N0B1J2	YgfZ	0.0299	*0.4667*			tRNA-modifying protein	3	C
A0A6N0AMQ7		0.0114	2.0908			DNA-binding protein H-NS	0	C
A0A6N0BB83		0.0224	3.2046			Elongation factor Tu	3	C
A0A6N0AJ97		0.0307	2.7311			Elongation factor Tu	3	C
A0A6N0ALX1		0.0057	3.0829			Peptide chain release factor 3	6	C
A0A6N0B620		0.0377	2.0594			Sulfite reductase (NADPH) flavoprotein alpha-component, cysteine biosynthesis	1	C
→ Nucleic acids and cofactor synthesis and homeostasis								
A0A6N0AB00	Apt	0.0208	*0.4420*			Adenine phosphoribosyl-transferase	1	C
A0A6N0BBV4	CspE	0.0051	*0.4385*			Cold shock-like protein	0	C
A0A6N0AQX1	IhfA	0.0172	2.0893			Integration host factor subunit alpha	0	C
A0A6N0BHM3		0.0232	2.0453			Exoribonuclease 2	7	C
→ Poliamine biosynthesis								
A0A6N0ASX1	SpeA	0.0102	2.5712			Biosynthetic arginine decarboxylase	8	EX
A0A2K8VWZ1 A0A6N0BJQ5	SpeE	0.0170	3.2343	0.0056	3.0386	Polyamine aminopropyltransferase	8	C
LIPOPROTEINS, MEMBRANE AND CELL WALL BIOGENESIS								
A0A6N0BJT9	ArnA			0.0129	2.4335	Bifunctional polymyxin resistance protein ArnA	7	C
A0A6N0AHH6	BamD	0.0005	2.6820			Outer membrane protein assembly factor	1	EX
A0A6N0AZB8	GlmU	0.0086	2.7221			Bifunctional protein, cell wall biogenesis	4	C
A0A6N0BB99	YbiS	0.0120	2.5079			L,D-transpeptidase	1	EX
A0A6N0ARZ3		0.0371	2.9322			Osmotically inducible lipoprotein E	4	EX
A0A6N0BHR4		0.0042	2.6863			UDP-N-acetylmuramate-L-alanine ligase	2	C
A0A6N0AV98		0.0025	3.6632			UDP-N-acetylmuramoyl-tripeptide-D-alanyl-D-alanine ligase	4	C
OTHERS, UNIDENTIFIED								
A0A2K8VZQ3 A0A6N0AK79	DcrB	0.0020	3.0076			Periplasmic bacteriophage sensitivity protein DcrB	1	EX
A0A6N0AWQ4	ZapB			0.0094	3.1295	Cell division protein ZapB	0	C
A0A2K8VTW3		0.0009	5.9074			Uncharacterized protein	3	N
A0A6N0AH49		0.0032	2.6561			Uncharacterized protein	2	N

UniProtID in gray are the archival numbers. The fold change values of downregulated proteins are in italics; number of cysteine residues in a protein (Number of. Cys); predicted protein localization: the cytoplasm (C), the extracytoplasmic space (EX), not known (N).

**Table 3:** Extracellular proteins whose level is downregulated due to the lack of a functional DsbA protein.

Peak Name	Name	*p*-Value	Fold Change	Description	Number of Cys
MOTILITY AND CHEMOTAXIS					
A0A6N0AH46	CheW	0.0168	*0.4267*	Chemotaxis protein CheW	0
A0A6N0AF74	CheY	0.0012	*0.0683*	Chemotaxis protein CheY	0
A0A6N0ADL9	FlgB	0.0006	*0.0973*	Flagellar basal body rod protein FlgB	0
A0A6N0B762	FlgC	<0.0001	*0.2802*	Flagellar basal body rod protein FlgC	0
A0A6N0ADS2	FlgD	0.0011	*0.1424*	Basal body rod modification protein FlgD	0
A0A6N0B2V1	FlgE	<0.0001	*0.1296*	Flagellar hook protein FlgE	0
A0A6N0BFR1	FlgF	<0.0001	*0.1241*	Flagellar basal body rod protein FlgF	0
A0A6N0B547	FlgG_1	0.0006	*0.0664*	Flagellar basal body rod protein FlgG	0
A0A6N0AH33	FlgK	<0.0001	*0.0324*	Flagellar hook-associated protein 1	0
A0A6N0ADK6	FlgL	<0.0001	*0.0458*	Flagellar hook-associated protein 3	0
A0A6N0AGC2	FlgM	<0.0001	*0.0238*	Negative regulator of flagellin synthesis	0
A0A6N0AG83	FliC_1	<0.0001	*0.0291*	Flagellin 1	0
A0A6N0ASL9	FliD	<0.0001	*0.0201*	Flagellar hook-associated protein 2	0
A0A6N0B5D6	FliE	<0.0001	*0.0420*	Flagellar hook–basal body complex protein FliE	0
A0A6N0B4U8	FliK	0.0013	*0.0256*	Flagellar hook-length control protein	0
VIRULENCE, ATTACHMENT					
A0A2K8VTS7		<0.0001	*0.0402*	Putative cellulase	2
A0A2K8VVJ2 A0A6N0BEF2		<0.0001	*0.0255*	Endo-1,4-beta-xylanase A	4
A0A6N0AEA4	PelA	<0.0001	*0.0118*	Pectate lyase A	10
A0A6N0ATI6	PelC_1	<0.0001	*0.0471*	Pectate lyase C	4
A0A6N0BJ90	PelC_2	<0.0001	*0.0227*	Pectate lyase C	4
A0A6N0ARF7	PelE	<0.0001	*0.0181*	Pectate lyase E	2
A0A6N0ANC2	PelL_1	<0.0001	*0.0255*	Pectate lyase L	3
A0A6N0AG85	PelL_2	0.0017	*0.0200*	Pectate lyase L	4
A0A2K8VZ10 A0A6N0AYE1		<0.0001	*0.0700*	Pectate lyase	1
A0A6N0B698	PemA	<0.0001	*0.1544*	Pectinesterase A	2
A0A6N0BBW4	Pnl	<0.0001	*0.0408*	Pectin lyase	3
A0A6N0B2L1	PrtA	0.0364	*0.3663*	Serralysin A	0
A0A2K8VWN4 A0A6N0BGU2		0.0114	*0.2795*	Neutral metalloproteinase	2
A0A6N0AC94	XynC	0.0003	*0.1446*	Glucuronoxylanase XynC	3

UniProtID in gray are the archival numbers. The fold change values of downregulated proteins are in italics.

**Table 4:** Bacterial strains and plasmids.

Strains	Relevant Genotype	Reference or Source
*Dickeya dadantii* 3937	WT	[88]
*D. dadantii* 3937 *dsbA::cm* (TP01)	*dsbA* gene inactivated by Cm^R^ insertion	This work
*D. dadantii* 3937 *dsbA-kn*	Complementation strain with a functional *dsbA* gene and a Kn^R^ cassette	This work
*D. solani* IPO2222	WT	[89]
*D. solani* IPO2222 *dsbA::cm* (TP02)	*dsbA* gene inactivated by Cm^R^	This work
*D. solani* IPO2222 *dsbA-kn* (TP03)	Complementation strain with functional *dsbA* gene and Kn^R^	This work
**Plasmids**	**Feature**	**Reference or Source**
pGEM-7	Cloning vector	Promega
pCYC-184-cm	Donor of Cm^R^ cassette	[90], New England Biolabs
pDOC-K	Donor of Kn^R^ cassette	[91]
pDF-dsbA::cm	pGEM-7 with *D. dadantii dsbA* gene inactivated by Cm^R^ cassette	This work
pDF-dsbA-kn	pGEM-7 with *D. dadantii* functional *dsbA* gene and Kn^R^ cassette	This work

**Table 5:** Primer sequences.

Name	Sequence
**Dd-dsbA-L**	GGACAATGACGACCAACTGGAG
**Dd-dsbA-R**	GCTCGCTCATTACGCTTTTTGC
**Cm-Sawitzke-L**	ACCAGCAATAGACATAAGCG
**Cm-Sawitzke-R**	TGTGACGGAAGATCACTTC
**Ds-dsbA-L**	GGCACCGATGTACTGACACTTA
**Ds-dsbA-R**	GCAGTAAGCAGAAATGTCCAAAC
**Kan-L**	GACCGGTCAATTGGCTGGAG
**Kan-R**	GAATATCCTCCTTAGTTCC

**Table 6:** Primers used for qPCR.

	FWD Primer Sequence (5′-3′)	REV Primer Sequence (5′-3′)	Amplicon Length	PCR Efficiency	R^2^	Concentration [µM]
*dsbA*	AACAGCATCAAACATCAGCGGG	TCTGGGAGAATTGGGTAAGGAGC	97	1.99	0.99	0.225
16S rRNA	GCTCGTGTTGTGAAATGTTGGGTT	GCAGTCTCCCTTGAGTTCCCAC	94	1.96	1.0	0.225

## Data Availability

The mass spectrometry proteomics data were deposited to the ProteomeXchange Consortium via the PRIDE [115] partner repository with the dataset identifier PXD028047.

## Supplementary Materials

The following are available online at https://www.mdpi.com/article/10.3390/ijms23020697/s1.
