# Supplementary material for: The Periplasmic Oxidoreductase DsbA Is Required for Virulence of the Phytopathogen Dickeya solani
Source: Int J Mol Sci. 2022 Jan 9;23(2):697. doi: 10.3390/ijms23020697 (PMC8775594; doi:10.3390/ijms23020697)
Supplement: Supplementary file 1 [file ijms-23-00697-s001.zip › ijms-1504896 suppl figures.pdf]

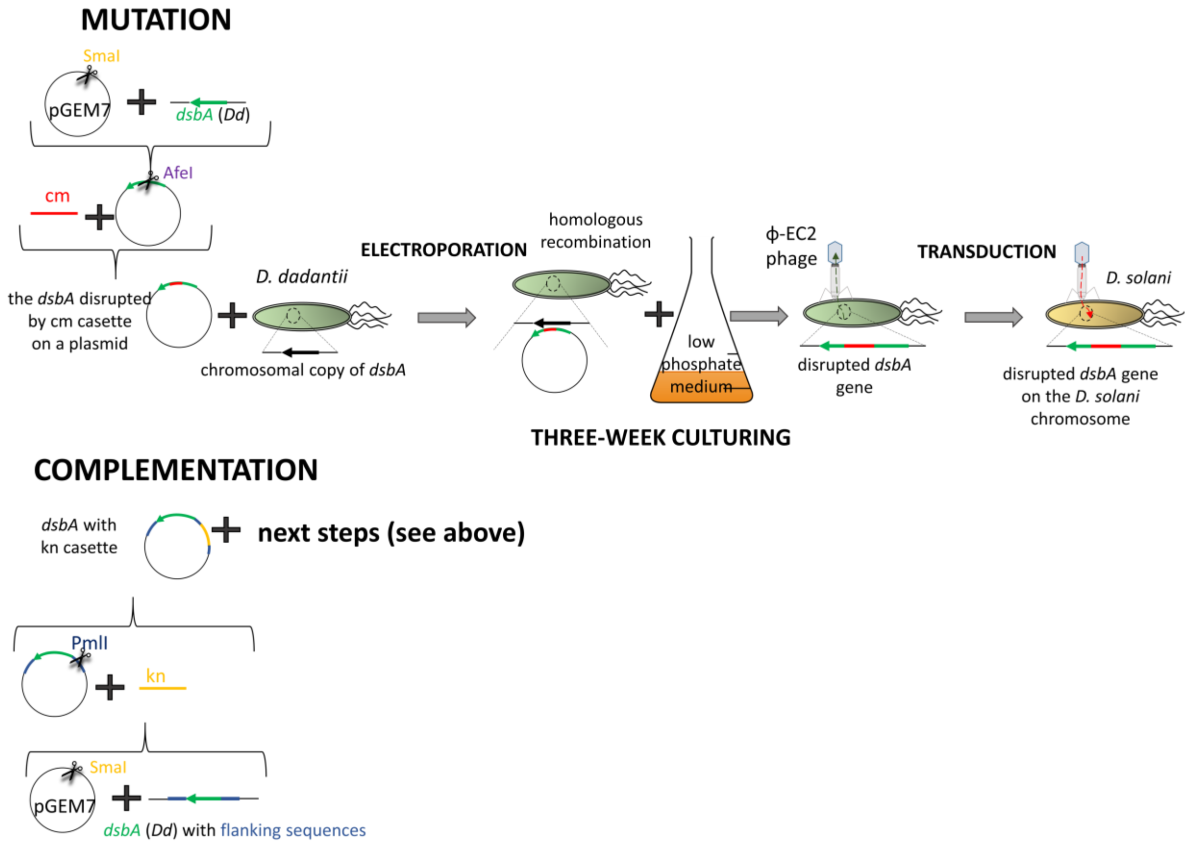

Figure S1. Scheme of the construction of the *D. solani* IPO222 *dsbA* mutant and complemented strains.

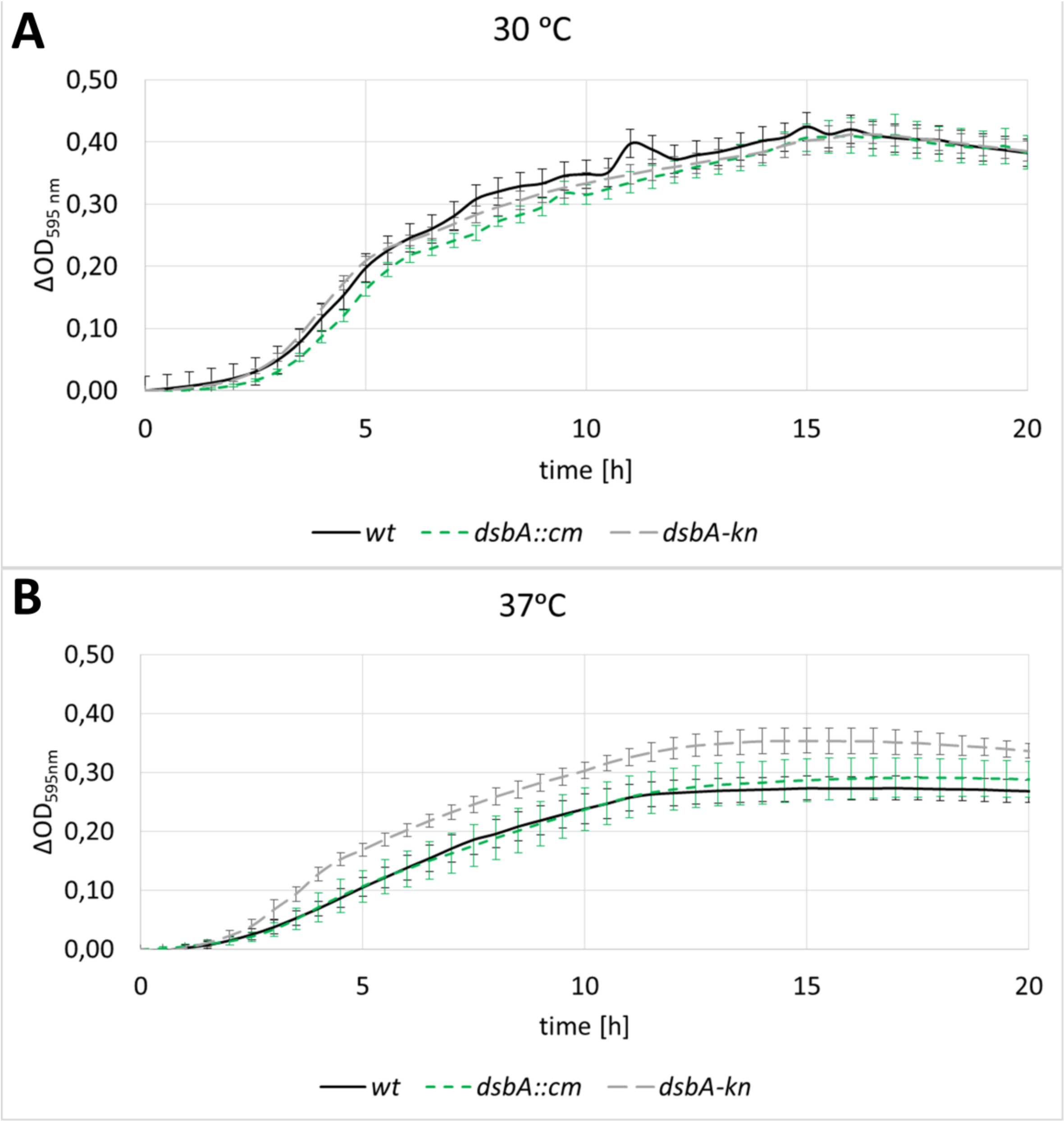

Figure S2. Growth curves of the *D. solani* IPO2222 strains and its derivatives. Growth curves were determined with the use of the EnSpire plate reader (PerkinElmer, Waltham, MA, USA) in a 24-well nontreated plate (#702011 Wuxi NEST Biotechnology Co., Ltd., Wuxi, China). The overnight cultures of the wild type (wt), *dsbA* mutant TP02, (dsbA) and complemented TP03 (comp) strains were diluted 1:50 with LB medium to a final volume of 1 ml and incubated at 30 °C (A) or 37 °C (B), with orbital shaking (120 rpm). The optical density was measured for 20 h and averaged for five biological replicates. The ΔOD_{595nm} values were obtained by subtraction of the OD values at time "0" from the values at the subsequent time points. The final values were averaged for five independent biological replicates.

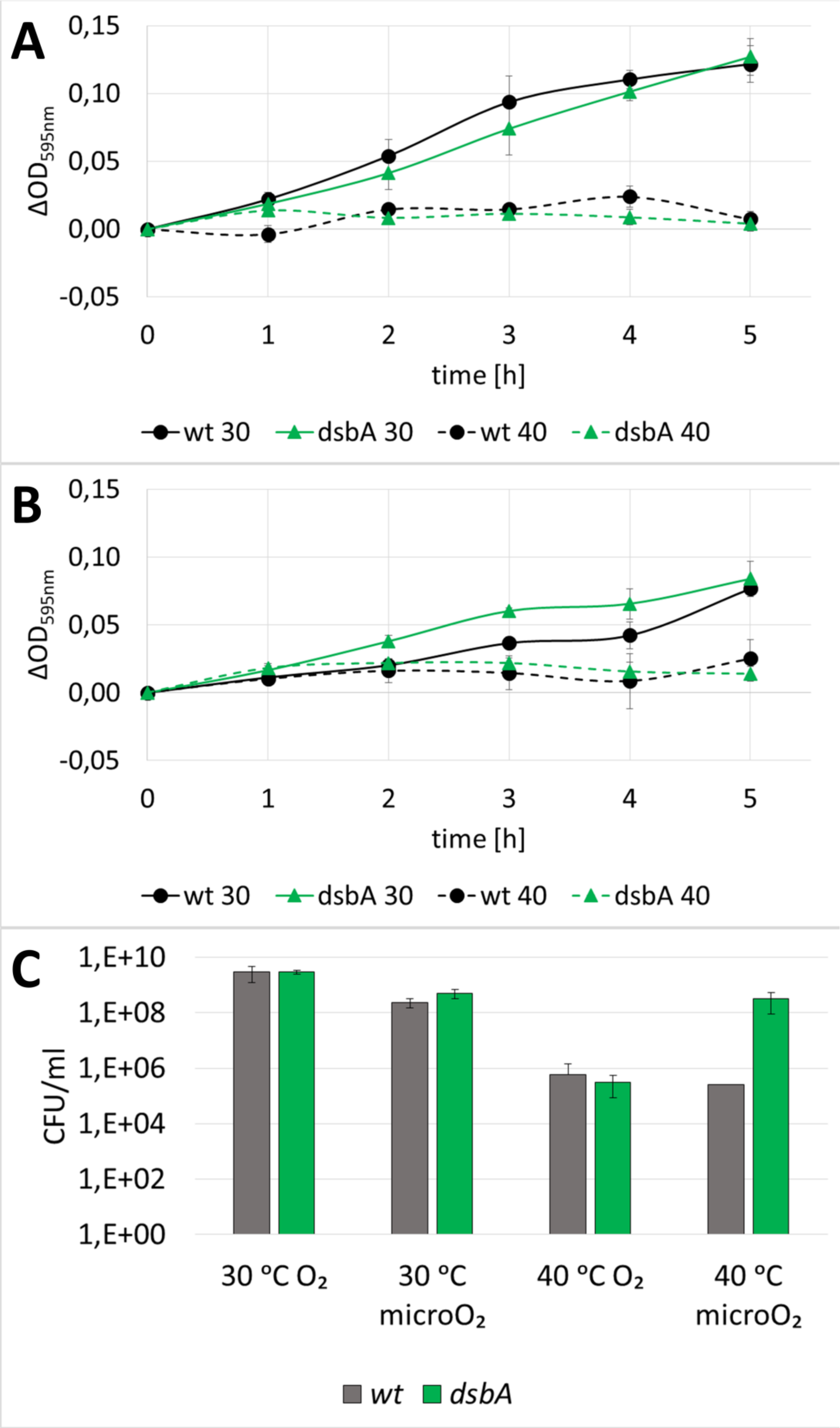
A
0,15
0,10
0,05
0,00
-0,05
ΔOD595nm
0
1
2
3
4
5
time [h]
wt 30
dsbA 30
wt 40
dsbA 40
B
0,15
0,10
0,05
0,00
-0,05
ΔOD595nm
0
1
2
3
4
5
time [h]
wt 30
dsbA 30
wt 40
dsbA 40
C
1,E+10
1,E+08
1,E+06
1,E+04
1,E+02
1,E+00
CFU/ml
30 °C O2
30 °C microO2
40 °C O2
40 °C microO2
wt
dsbA

Figure S3. D. solani IPO2222 wild type and dsbA mutant (TP02) growth and viability at elevated temperature (40 oC). Growth curves of the exponential (A) and stationary (B) phase cultures at 30 ºC (control) or 40 ºC. Viability was evaluated by measurement of CFU of bacteria growth on M63Y plates at 40 ºC under aerobic or microaerobic conditions (C). Error bars represent SD from three independent experimental repetitions.

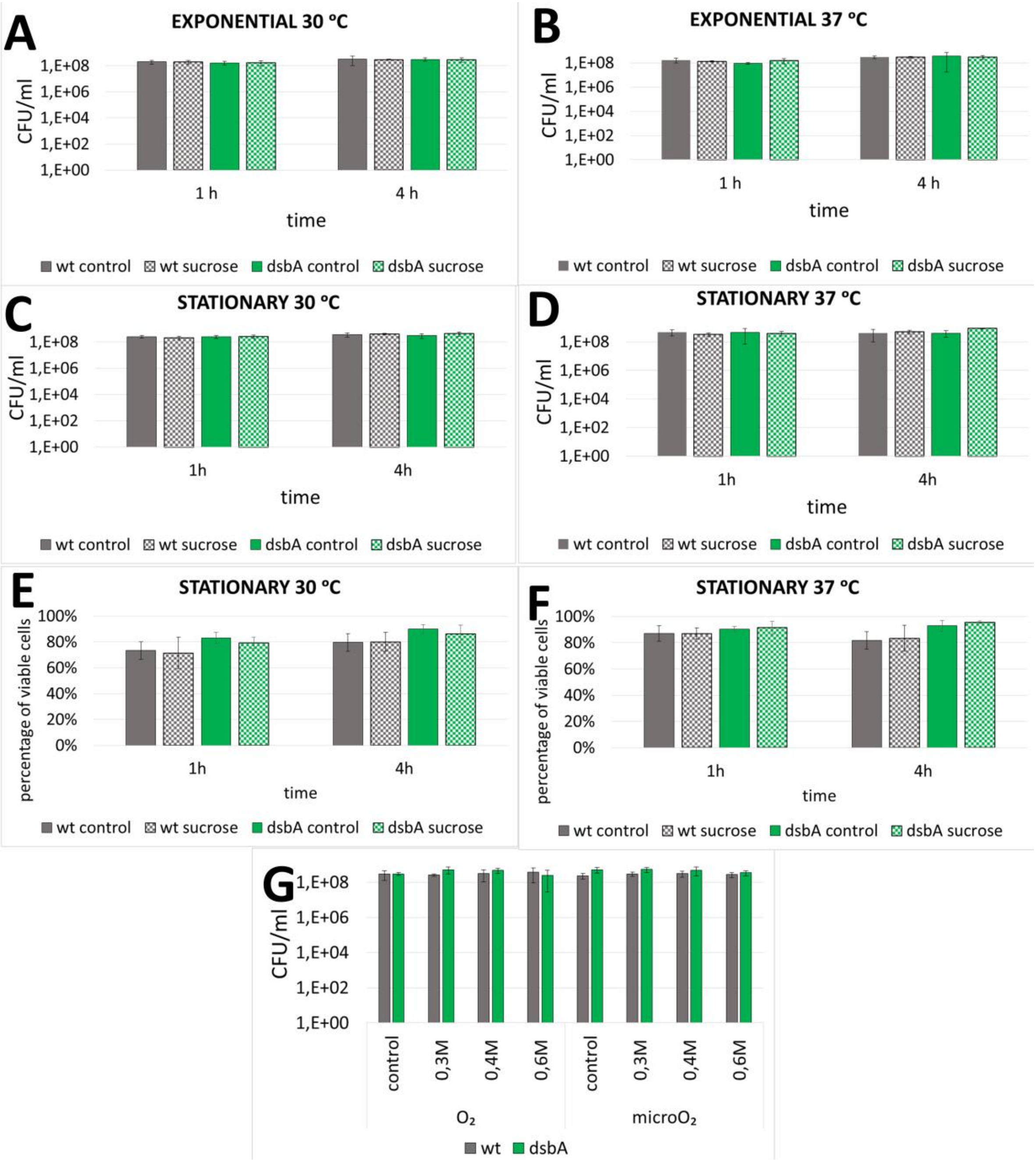

Figure S4. Resistance of D. solani IPO2222 wild type and dsbA mutant (TP02) to osmotic non-ionic stress in the exponential (A and B), stationary (C-F) growth phase. Viability was measured as a CFU ml-1 (A-D) or percentage of viable cells in live/dead microscopic test (E and F). Panel G shows CFU enumeration of bacteria exposed to different concentrations of sucrose in solid medium. In the CFU tests, error bars represent SD from three independent experimental repetitions; in the live-dead assay,

data obtained from three independent biological replicates with at least three technical replicates per sample were analyzed.

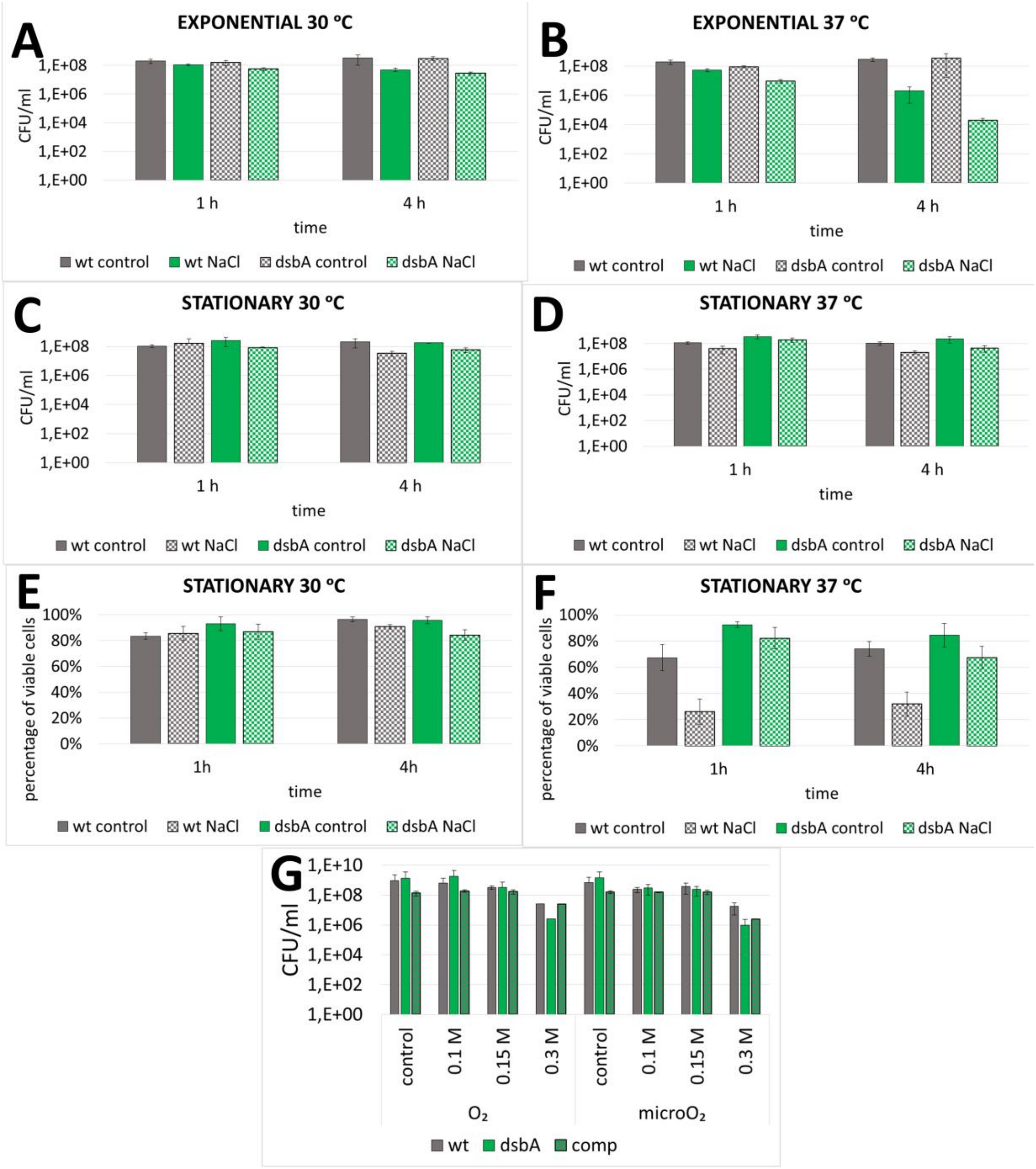

Figure S5. Resistance of D. solani IPO2222 wild type and dsbA mutant (TP02) to salt stress in the exponential (A and B), stationary (C-F) growth phase. Viability was measured as a CFU ml-1 (A-D) or percentage of viable cells in live/dead microscopic test (E and F). Panel G shows growth of bacteria exposed to different concentration of NaCl in solid medium (comp – complemented strain, TP03). In the CFU tests, error bars represent SD from three independent experimental repetitions; in the live-dead assay, data obtained from three independent biological replicates with at least three technical replicates per sample were analyzed.

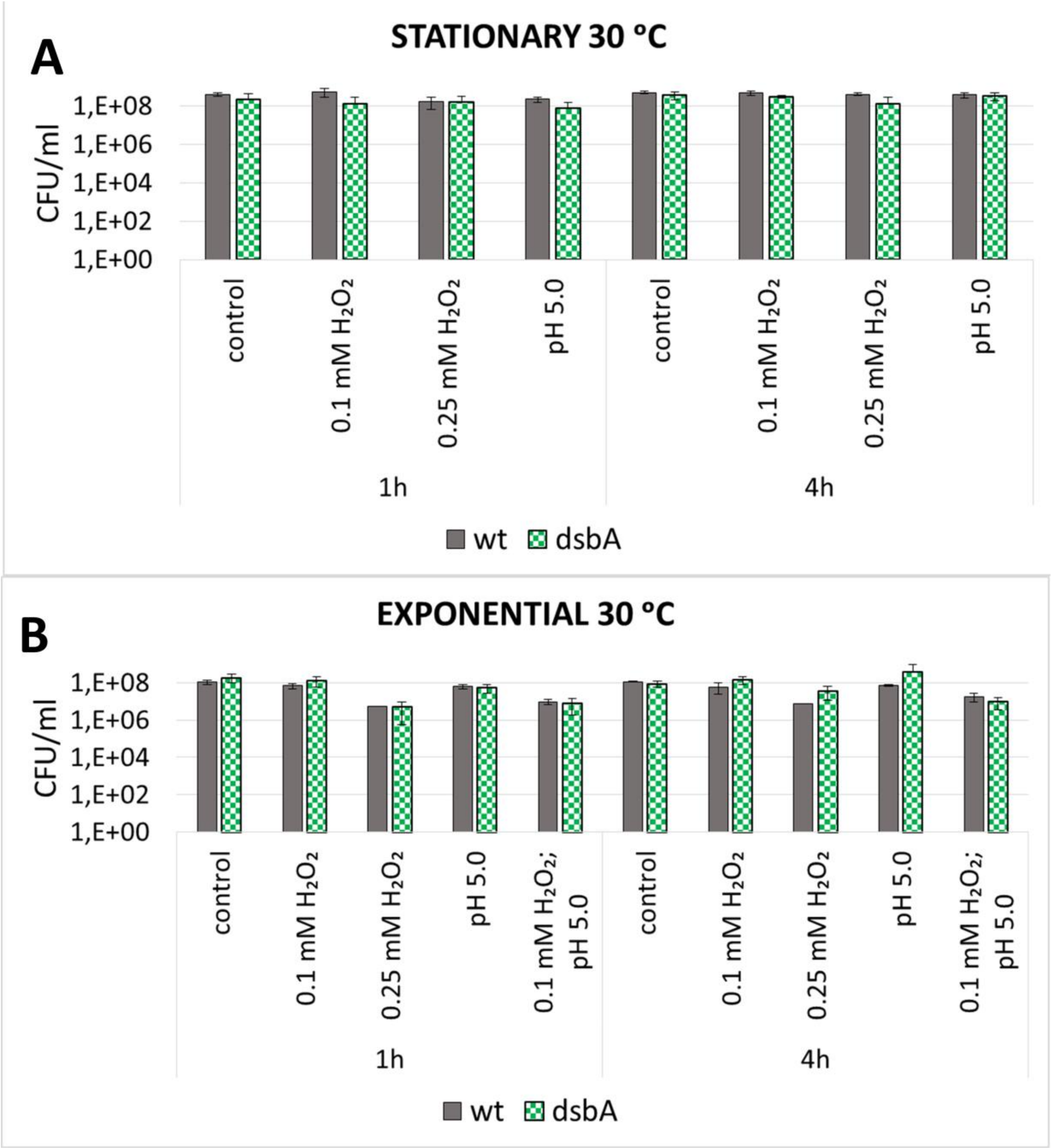

Figure S6. Resistance of D. solani IPO2222 wild type and dsbA mutant to oxidative or acidic stress in the exponential (A) or stationary growth phase (B) at optimal temperature. The error bars represent SD from three experimental repetitions.

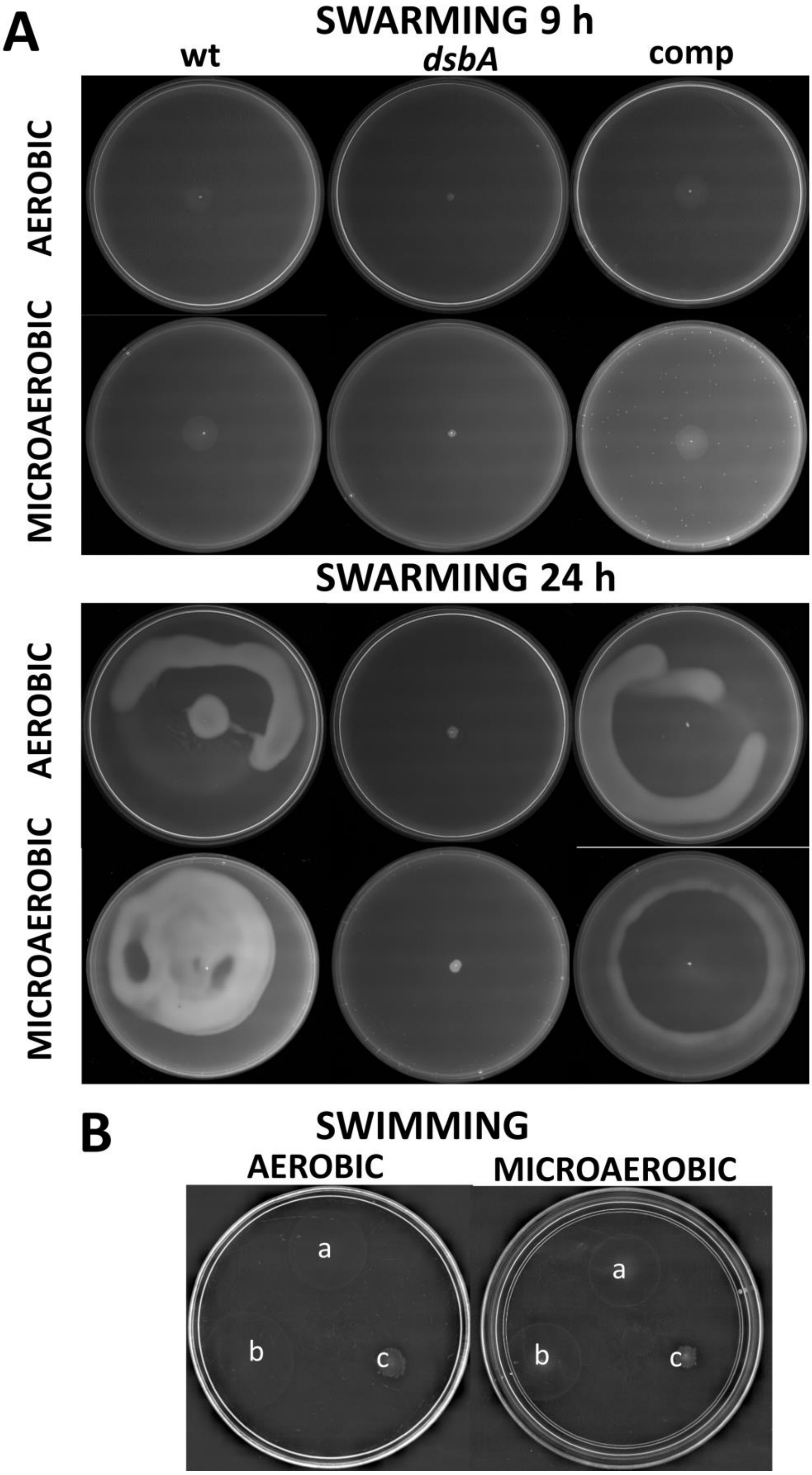

Figure S7. Effect of the dsbA knockout on the motility of D. solani. Representative examples of swarming (A) and swimming (B) plates; wt – wild type strain; dsbA – mutant strain TP02; comp –

complemented strain TP03; (a - D. solani wild type strain; b – complemented strain TP03; c – dsbA mutant TP02); wt – wild type strain; dsbA – mutant strain TP02; comp – complemented strain TP03.

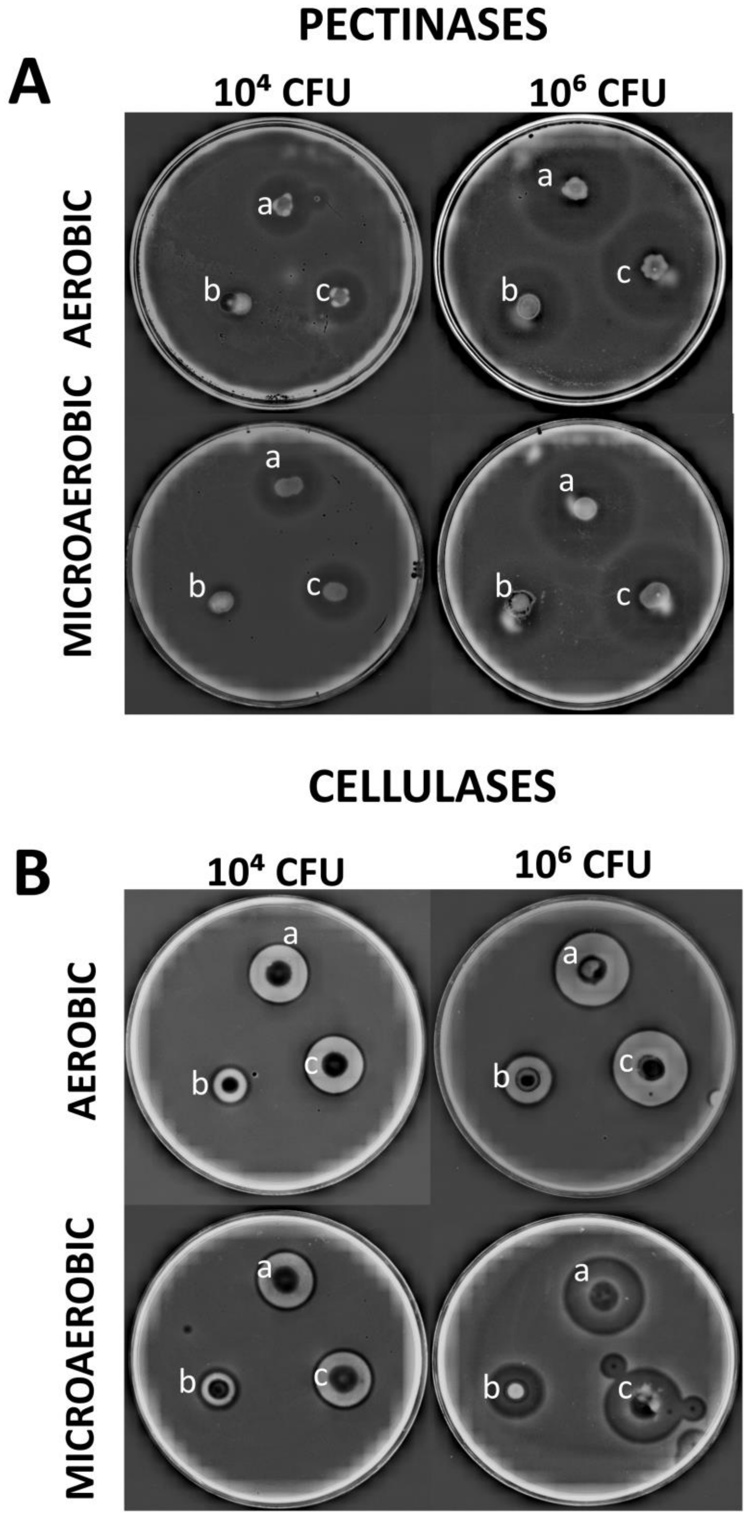

Figure S8. Impact of the *dsbA* knockout on the virulence factors of the *D. solani* secretome: (A) (B) Representative examples of the pectinase (A) and cellulase (B) plate assay (a - *D. solani* wild type strain; b - *dsbA* mutant TP02; c - complemented strain TP03); wt – wild type strain; *dsbA* – mutant strain TP02; comp – complemented strain TP03.

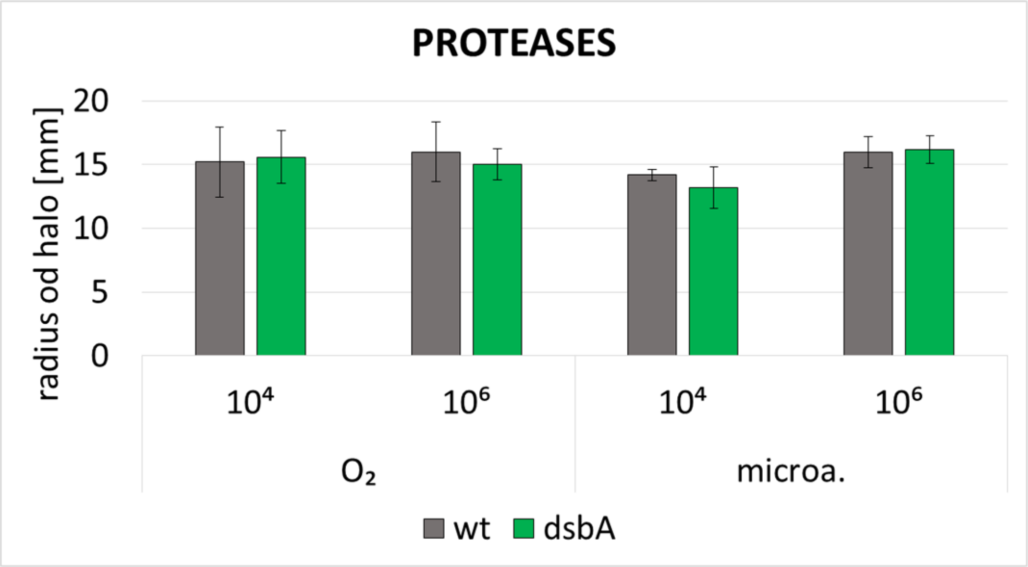

Figure S9. Secreted proteolytic activity of the *dsbA* mutant and wt *Dickeya solani* IPO2222. The error bars represent SD from five independent experimental repetitions.

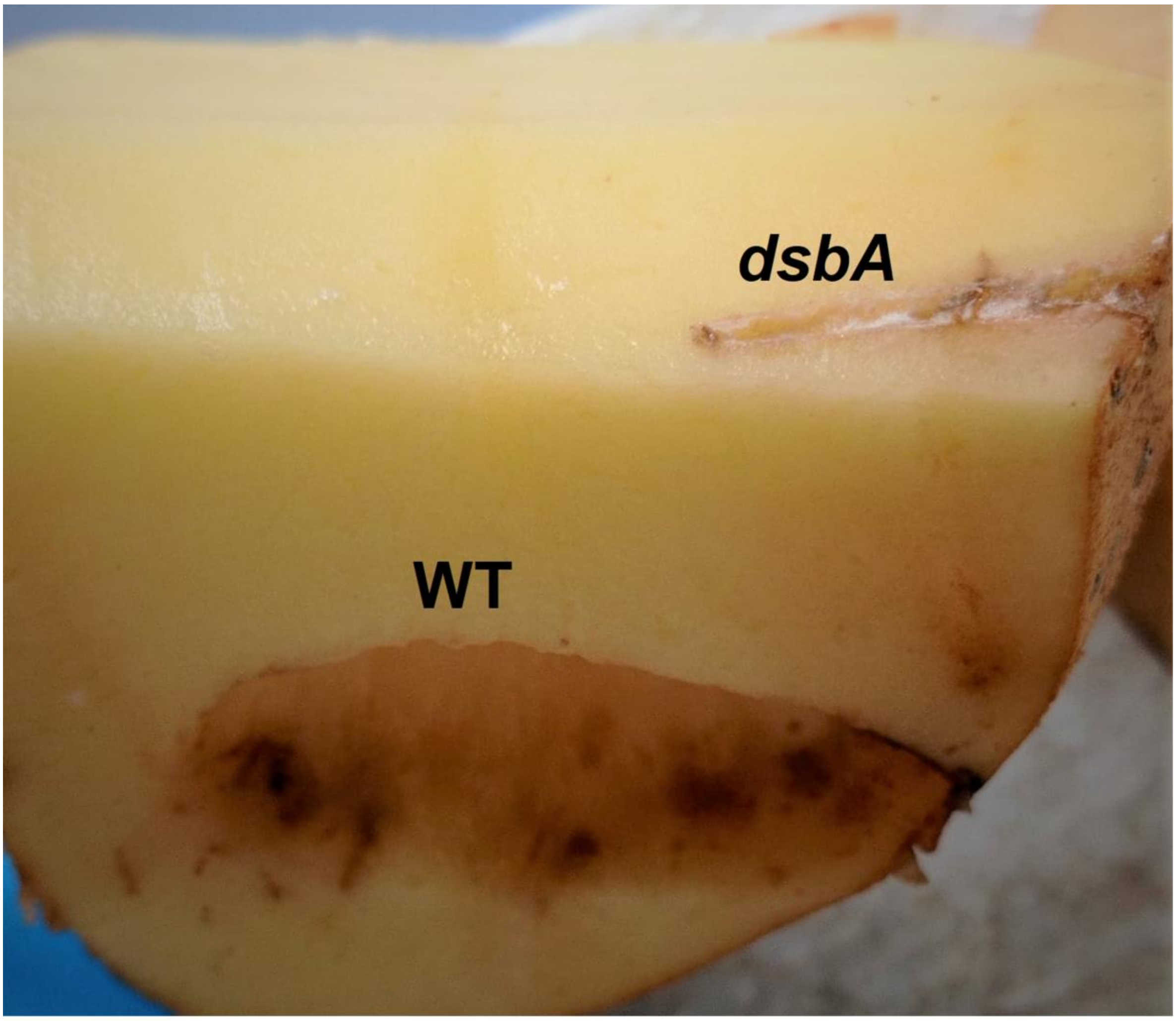

Figure S10. Potato tuber maceration caused by the D. solani IPO2222 strains (holes after removal of the macerated tissue).

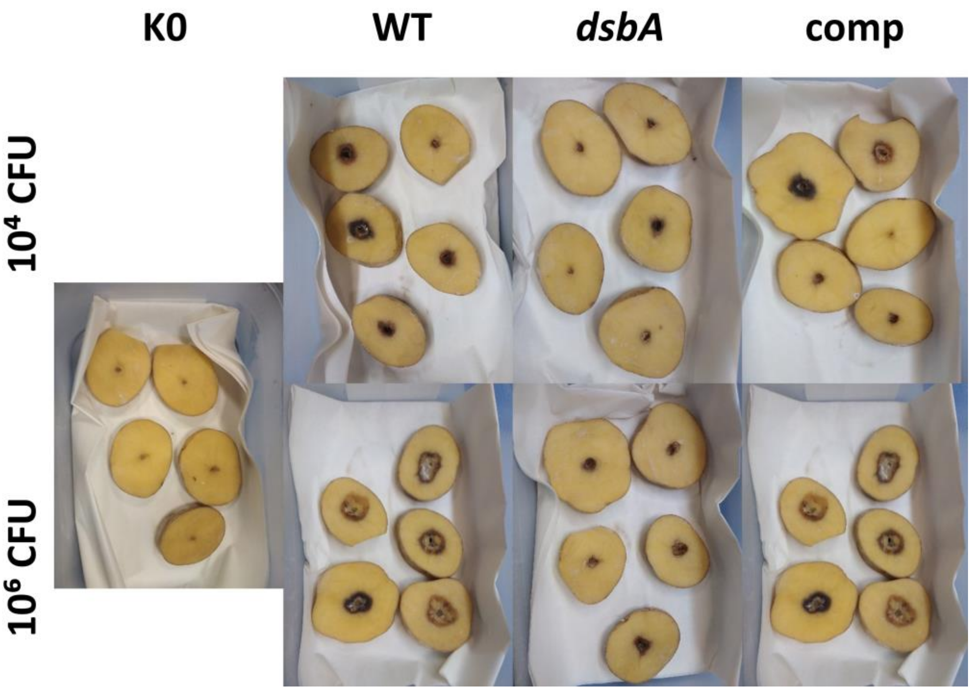

Figure S11. Potato tuber slices maceration by the *D. solani* IPO2222 strains (wt – wild type strain, *dsbA* – mutant strain, TP02; comp – complemented strain, TP03).

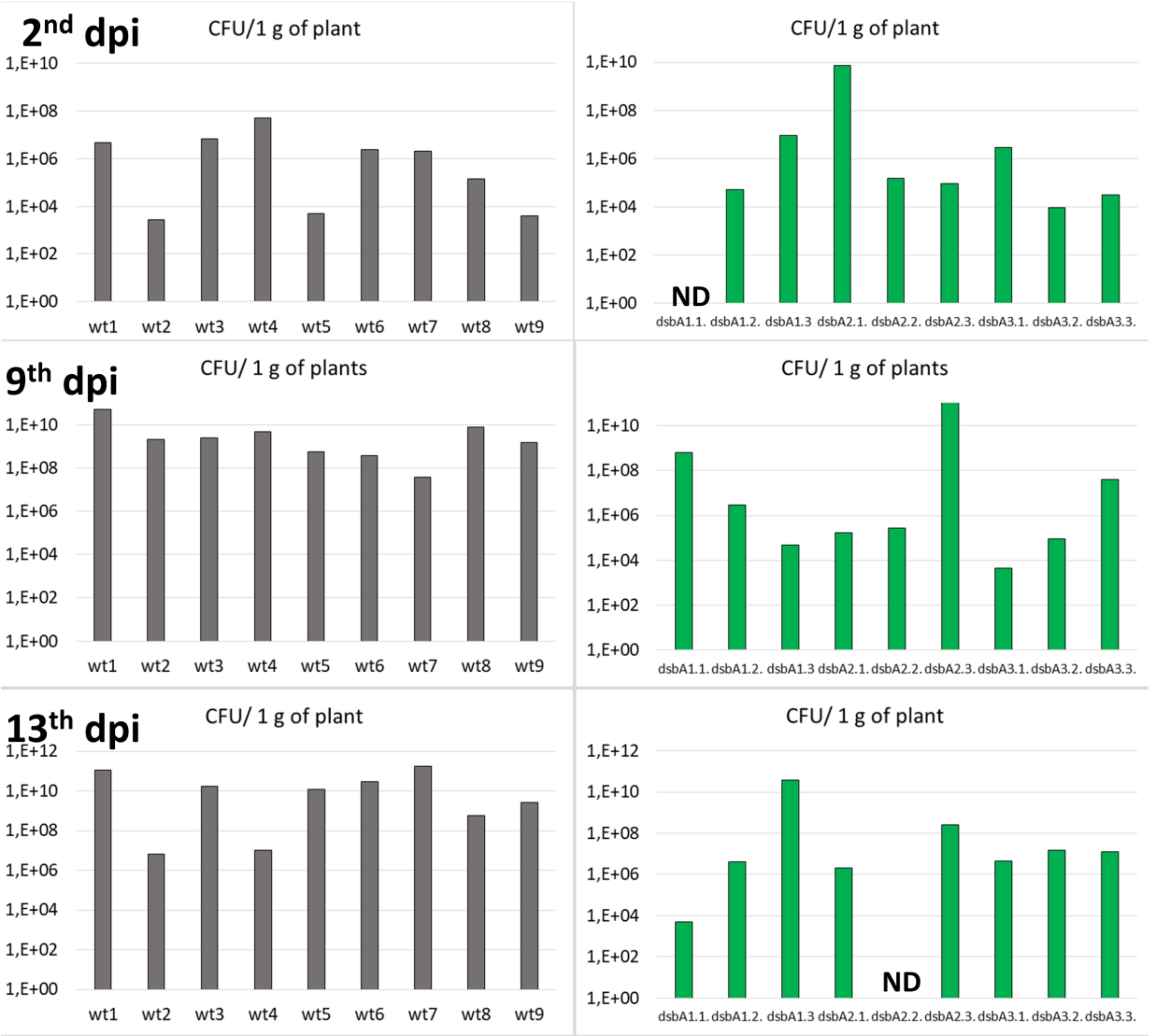

MEDIAN CFU/ 1 g of plant					
2nd dpi	9th dpi	13th dpi	2nd dpi	9th dpi	13th dpi
2,13E+06	2,01E+09	1,17E+10	1,23E+05	2,80E+05	8,48E+06
STANDARD DEVIATION					
1,60E+07	1,55E+10	6,32E+10	2,47E+09	5,91E+10	1,35E+10

Figure S12. *In vitro* infection assay. CFU counts in plantlets' tissues 2, 9 or 13 days post-infection (dpi). The Dunett test performed on the median CFU/1g values of wild type and *dsbA* mutant (TP02) after discarding the ND values did not show significant differences (2 dpi: 0.306685; 9th dpi: 0.553635; 13th dpi: 0.157768). ND, notdetected; dsbA1, dsbA2, dsbA3 stand for the three independently obtained *dsbA* knockout strains.
