# Supplementary material for: The Periplasmic Oxidoreductase DsbA Is Required for Virulence of the Phytopathogen Dickeya solani
Source: Int J Mol Sci. 2022 Jan 9;23(2):697. doi: 10.3390/ijms23020697 (PMC8775594; doi:10.3390/ijms23020697)
Supplement: Supplementary file 1 [file ijms-23-00697-s001.zip › supplemenatry table caption.pdf]

Table S1. Proteomic comparison of the D. solani dsbA and the parental D. solani IPO2222 grown under non-stressful or oxidative stress conditions. Green aeras: p-value x<0.05; red areas: fold change 2.0<y<0.5. For the analysis, the Dickeya solani D s0432-1 database from Uniprot.org on the 19th of October 2020 was used.

Table S2. List of all proteins quantified in the *Dickeya solani* IPO2222 secretomes. Protein ratios of *dsbA* mutant to the wt strain are presented (fold change). Green aeras: p-value x<0.05; red areas: fold change 2.0<y<0. For the analysis, the *Dickeya solani* D s0432-1 database from Uniprot.org on the 19th of October 2020 was used.
