# Supplementary material for: The Periplasmic Oxidoreductase DsbA Is Required for Virulence of the Phytopathogen Dickeya solani
Source: Int J Mol Sci. 2022 Jan 9;23(2):697. doi: 10.3390/ijms23020697 (PMC8775594; doi:10.3390/ijms23020697)
Supplement: Supplementary file 1 [file ijms-23-00697-s001.zip › Table_S1.pdf]

Index	UniProt ID	Name/Description	NON-STRESSFUL CONDITIONS: dsbA/wt		wt strain: OXIDATIVE STRESS/NON-S. CONDITIONS		dsbA strain: OXIDATIVE STRESS/NON-S. CONDITIONS	
			p-value	Fold Change	p-value	Fold Change	p-value	Fold Change
1	A0A6N0AV44	DNA-directed RNA polymerase subunit beta	0,97540	1,00288	0,04044	1,16126	0,09010	1,18265
2	A0A6N0A6S9	DNA-directed RNA polymerase subunit beta	0,00540	1,22724	0,16907	0,95291	0,05006	0,86422
3	A0A6N0ANI6	60 kDa chaperonin	0,02668	0,94556	0,00168	0,85170	0,19121	0,93837
4	A0A6N0ABM2	30S ribosomal protein S1	0,21829	0,85591	0,01967	0,79017	0,76208	0,96468
5	A0A6N0AVZ8	Glycerol kinase	0,00678	0,82840	0,75861	0,97472	0,59990	0,96380
6	A0A6N0ARG9	Outer membrane protein F	0,34767	1,04808	0,17244	1,06588	0,83256	1,01147
7	A0A6N0BHM0	Aerobic glycerol-3-phosphate dehydrogenase	0,13188	1,04578	0,00286	0,87503	0,07641	0,92173
8	A0A6N0ALD5	Glutamate synthase [NADPH] large chain	0,95753	0,99126	0,00613	0,84043	0,61829	0,89796
9	A0A6N0ABI9	Formate acetyltransferase 1	0,01436	0,81264	0,01965	0,86248	0,00905	0,76363
10	A0A6N0AJI3	Elongation factor G	0,01312	1,09812	0,22494	1,03271	0,05482	1,04030
11	A0A6N0AV51	5-methyltetrahydropteroyltriglutamate--homocysteine methyltransferase	0,18654	0,86388	0,70150	1,02456	0,29024	1,10241
12	A0A6N0ASH5	Aconitate hydratase 2	0,56540	0,95404	0,08965	0,92351	0,06337	0,80340
13	A0A6N0B4P0	Phosphoenolpyruvate synthase	0,00826	1,35367	0,90814	0,99183	0,97937	0,99879
14	A0A6N0AJ97	Elongation factor Tu	0,00511	3,99424	0,03074	2,73112	0,11803	0,79810
15	A0A6N0BKU6	Chaperone protein DnaK	0,67410	1,05016	0,44913	0,96429	0,94320	0,99246
16	A0A6N0AI53	ATP synthase subunit alpha	0,54449	0,92445	0,26005	1,07257	0,83296	1,03045
17	A0A6N0BFA5	Outer membrane protein A	0,03895	1,29758	0,00018	0,78498	0,53269	0,94113
18	A0A6N0BE12	Glyceraldehyde-3-phosphate dehydrogenase A	0,00036	1,35572	0,00039	0,86487	0,01068	0,81880
19	A0A6N0AIK9	Chaperone protein ClpB	0,67667	1,04162	0,62758	1,04727	0,43166	0,97388
20	A0A6N0B4R5	Pyruvate dehydrogenase E1 component	0,02077	0,90362	0,01724	1,10571	0,12521	1,05126
21	A0A6N0AIL9	Carbamoyl-phosphate synthase large chain	0,03371	0,66265	0,31982	1,11689	0,16791	1,39752
22	A0A6N0ANU7	Trigger factor	0,77830	0,98702	0,01992	0,84653	0,00393	0,86485
23	A0A6N0AEN7	Periplasmic oligopeptide-binding protein	0,01942	0,80756	0,06400	1,11833	0,01886	1,13637
24	A0A6N0B9R8	ATP synthase subunit beta	0,62531	0,96323	0,05463	1,14672	0,01692	1,20470

25	A0A6N0BI17	Enolase	0,05407	0,90823	0,00130	1,14325	0,10192	1,14314
26	A0A6N0AF73	Isocitrate dehydrogenase [NADP]	0,09894	0,87435	0,01223	0,77865	0,14624	0,90862
27	A0A6N0BJS7	Ketol-acid reductoisomerase	0,23208	0,93701	0,12865	1,09094	0,08624	1,07157
28	A0A6N0AUU7	Transketolase 1	0,04046	1,19073	0,00105	1,27717	0,61562	1,04443
29	A0A6N0BEV9	Lon protease	0,00110	1,32679	0,00042	1,36665	0,09818	1,04195
30	A0A6N0AAG6	2-oxoglutarate dehydrogenase E1 component	0,50479	0,96401	0,00148	0,65557	0,00837	0,79031
31	A0A6N0AIR3	Phosphoglycerate kinase	0,03988	1,31629	0,85785	0,98762	0,36821	0,89974
32	A0A6N0B6V8	Outer membrane protein F	0,00576	0,86939	0,00000	0,23332	0,03278	0,54027
33	A0A6N0AAL1	6-phosphogluconate dehydrogenase, decarboxylating	0,00496	1,46423	0,02114	1,33624	0,50815	1,03486
34	A0A6N0B712	DNA-directed RNA polymerase subunit alpha	0,97027	1,00171	0,02045	0,84084	0,02825	0,89482
35	A0A6N0AME6	Chaperone protein HtpG	0,35814	0,94796	0,00002	0,75888	0,28798	0,88702
36	A0A6N0B0F6	Aldehyde-alcohol dehydrogenase	0,00216	1,18886	0,09360	0,94741	0,60077	0,98873
37	A0A6N0AMD5	Polyribonucleotide nucleotidyltransferase	0,15596	1,09491	0,14010	1,09845	0,75320	1,00788
38	A0A6N0AG29	Phenylalanine--tRNA ligase beta subunit	0,08863	1,18504	0,07528	0,85110	0,10983	0,87332
39	A0A6N0AQ96	Ribonuclease E	0,76024	1,04996	0,23910	1,20162	0,05422	1,13127
40	A0A6N0BAV5	Periplasmic pH-dependent serine endoprotease DegQ	0,00189	1,53756	0,03331	1,20218	0,06586	1,08213
41	A0A6N0AKK0	Glutamine synthetase	0,01028	1,34443	0,75312	1,03011	0,08691	1,12320
42	A0A6N0AYX3	Malate dehydrogenase	0,05079	0,66959	0,14484	0,88112	0,07877	1,36210
43	A0A6N0AN74	Phosphoserine aminotransferase	0,00143	1,28587	0,00001	1,32105	0,03437	1,09152
44	A0A6N0APB6	Succinyl-CoA ligase [ADP-forming] subunit beta	0,81394	0,96802	0,01957	0,54421	0,00481	0,73196
45	A0A6N0BHD7	Phosphoribosylformylglycinamidine synthase	0,76638	0,95093	0,12809	1,16195	0,28282	1,17260
46	A0A6N0AC21	Asparagine synthetase B [glutamine-hydrolyzing]	0,01936	1,24695	0,00192	1,45123	0,00293	1,38565
47	A0A6N0AD88	Citrate synthase	0,12172	1,05496	0,03503	1,10477	0,00943	1,08017
48	A0A6N0B1Z2	Pyruvate kinase II	0,18214	0,89585	0,01634	0,74021	0,01126	0,74788
49	A0A6N0AXP2	Transaldolase B	0,00028	1,28985	0,08865	1,05661	0,23608	1,01945
50	A0A6N0BAU3	Cysteine synthase A	0,23226	0,96047	0,13146	0,95297	0,83718	1,01195
51	A0A6N0BBN8	Translation initiation factor IF-2	0,09853	1,16933	0,98043	0,99795	0,04957	0,90269
52	A0A6N0AG83	Flagellin 1	0,00006	0,00798	0,01857	1,22990	0,58581	0,81741
53	A0A6N0AVH0	Threonine synthase	0,05298	1,43294	0,00952	1,30772	0,49949	1,09573
54	A0A6N0BH82	Alanine--tRNA ligase	0,06149	0,90560	0,00074	0,65782	0,00008	0,82331
55	A0A6N0API8	2-hydroxypropyl-CoM lyase	0,40183	0,81821	0,55116	0,88228	0,16609	1,16203

56	A0A6N0B2K0	Peptidyl-prolyl cis-trans isomerase D	0,03349	1,12083	0,00152	1,17428	0,15375	1,05169
57	A0A6N0BIA0	Serine hydroxymethyltransferase	0,15339	1,19836	0,23434	1,12765	0,96873	0,99558
58	A0A6N0AL81	Uncharacterized protein	0,01621	1,24265	0,05486	1,16720	0,90844	0,99467
59	A0A6N0ACS4	Alcohol dehydrogenase 2	0,52742	1,03628	0,00381	0,69153	0,00062	0,70075
60	A0A6N0AS49	Phosphate acetyltransferase	0,01727	1,17495	0,00020	1,38466	0,04156	1,10924
61	A0A6N0BB31	Outer membrane protein assembly factor BamA	0,00193	2,50341	0,15048	1,51768	0,01603	0,74658
62	A0A6N0B776	Dihydrolipoyl dehydrogenase	0,01738	0,88683	0,01873	0,89444	0,86803	1,00473
63	A0A6N0B8D8	Glycerophosphoryl diester phosphodiesterase	0,08901	0,89092	0,00950	0,87175	0,11724	0,85472
64	A0A6N0BIQ5	50S ribosomal protein L2	0,18908	0,80458	0,05476	1,25985	0,24992	1,26576
65	A0A6N0AZ67	Elongation factor Ts	0,57395	0,93856	0,36352	1,08240	0,91831	1,01024
66	A0A6N0ASY5	Adenylosuccinate synthetase	0,02886	1,27531	0,77280	1,01600	0,10544	0,88120
67	A0A6N0BBJ0	Rod shape-determining protein MreB	0,26019	1,08211	0,01803	0,85523	0,11128	0,82258
68	A0A6N0B7Z8	Bifunctional aspartokinase/homoserine dehydrogenase 1	0,05169	1,26556	0,13135	1,14842	0,02853	1,21066
69	A0A6N0ABE3	Uncharacterized protein	0,73345	1,04516	0,57911	1,07493	0,01485	1,22789
70	A0A6N0AY02	Succinate dehydrogenase flavoprotein subunit	0,00185	1,62433	0,01602	1,32048	0,19260	0,91829
71	A0A6N0B8L0	GDP-mannose 4,6-dehydratase	0,04513	0,88186	0,80536	1,00575	0,14953	1,08397
72	A0A6N0BEK9	Fe(3+)-binding periplasmic protein	0,01517	0,86268	0,00016	0,70901	0,00071	1,35190
73	A0A6N0ARJ8	Sulfate-binding protein	0,97943	0,99770	0,00715	1,48482	0,01447	1,17077
74	A0A6N0BES5	2,3-bisphosphoglycerate-dependent phosphoglycerate mutase	0,03305	0,59693	0,20416	0,83593	0,06392	1,29878
75	A0A6N0BK46	D-methionine-binding lipoprotein MetQ	0,65935	0,97084	0,00528	1,23420	0,11950	1,17955
76	A0A6N0B818	Fructose-bisphosphate aldolase class 2	0,07492	0,87794	0,00001	0,54639	0,04051	0,81284
77	A0A6N0AVX9	30S ribosomal protein S3	0,07731	0,86400	0,00146	0,74121	0,12935	0,84738
78	A0A6N0B031	50S ribosomal protein L1	0,13434	0,77733	0,02383	1,23028	0,26780	1,23407
79	A0A6N0AQD6	Glutamate/aspartate periplasmic-binding protein	0,00100	0,27062	0,04980	1,22894	0,04656	1,44403
80	A0A6N0AW84	ATP-dependent zinc metalloprotease FtsH	0,11323	1,26378	0,30633	0,91142	0,21753	0,82698
81	A0A6N0AXG5	30S ribosomal protein S2	0,01637	0,72755	0,00001	0,57659	0,25821	0,86902
82	A0A6N0AW60	Inosine-5'-monophosphate dehydrogenase	0,32337	1,24490	0,34866	0,93256	0,36118	0,79461
83	A0A6N0A960	Protein translocase subunit SecD	0,12062	1,11791	0,59279	1,03020	0,31493	1,04960
84	A0A6N0ABE6	ATP-dependent Clp protease ATP-binding subunit ClpA	0,02417	1,44457	0,01595	1,46119	0,37851	0,89943

85	A0A6N0ABA8	Dihydrolipoyllysine-residue succinyltransferase component of 2-oxoglutarate dehydrogenase complex	0,10130	0,90403	0,10840	0,89535	0,42372	0,95381
86	A0A6N0AHF5	50S ribosomal protein L5	0,34974	0,93466	0,01541	0,78817	0,05677	0,80154
87	A0A6N0AVV3	Uncharacterized protein	0,06609	1,10274	0,34293	0,94981	0,03539	0,91492
88	A0A6N0BLC9	Dihydrolipoyllysine-residue acetyltransferase component of pyruvate dehydrogenase complex	0,00286	0,60251	0,00218	1,43888	0,00110	1,97245
89	A0A6N0ADN0	Glutamine-binding periplasmic protein	0,31959	1,17636	0,06344	1,40607	0,03750	1,07269
90	A0A6N0AUY9	Putative amino-acid-binding protein YxeM	0,39363	0,81852	0,03462	1,23719	0,37091	1,26423
91	A0A6N0B831	50S ribosomal protein L4	0,00300	0,89739	0,01068	0,88450	0,00242	0,83553
92	A0A6N0BAJ7	Oligopeptidase A	0,33912	1,13932	0,17178	1,14499	0,95942	1,00506
93	A0A6N0BBY6	Putrescine-binding periplasmic protein	0,96143	0,99548	0,00675	1,53891	0,24355	1,09353
94	A0A6N0BB30	Outer membrane protein TolC	0,16787	1,04551	0,15847	1,05698	0,55416	1,03786
95	A0A6N0AGR8	30S ribosomal protein S4	0,08112	0,82698	0,19170	1,05532	0,48342	1,07566
96	A0A6N0B591	3-oxoacyl-[acyl-carrier-protein] synthase 1	0,02950	1,22621	0,01032	1,54975	0,00028	1,25368
97	A0A6N0AKS4	Putative peroxiredoxin	0,30275	0,74345	0,52678	0,95516	0,70985	1,13137
98	A0A6N0AAK8	Chemotaxis protein CheV	0,00619	2,11673	0,03297	1,81756	0,00032	0,83983
99	A0A6N0AXW9	50S ribosomal protein L10	0,04600	0,90027	0,00032	0,64283	0,00695	0,86759
100	A0A6N0AJK1	Aspartate ammonia-lyase	0,03169	1,07893	0,62309	1,01082	0,00404	0,79247
101	A0A6N0A824	Argininosuccinate synthase	0,21668	1,12189	0,12145	1,16231	0,58139	0,97251
102	A0A6N0AIL4	Putative amino-acid ABC transporter-binding protein	0,00039	1,47094	0,00577	1,62501	0,00118	1,15741
103	A0A6N0BNN1	Leu/Ile/Val-binding protein	0,13489	0,57630	0,88694	1,03019	0,19001	1,34965
104	A0A6N0BBA1	5-methyltetrahydropteroyltriglutamate--homocysteine methyltransferase	0,06529	0,93214	0,41207	0,98323	0,44093	0,97152
105	A0A6N0BCF9	D-galactose-binding periplasmic protein	0,00443	1,27894	0,03917	1,17555	0,00495	1,25458
106	A0A6N0ATR5	Sulfite reductase [NADPH] hemoprotein beta-component	0,89372	0,98661	0,45287	1,07636	0,64531	0,96342
107	A0A6N0BMR8	Phosphoenolpyruvate carboxykinase [ATP]	0,12862	1,31476	0,02635	1,36762	0,44558	1,08869
108	A0A6N0BDZ0	Pyruvate kinase I	0,13423	0,85900	0,00492	0,54801	0,00459	0,72216
109	A0A6N0A9V6	Glutathione-binding protein GsiB	0,69885	1,02406	0,00758	1,21432	0,33070	0,94482
110	A0A6N0AFA2	Uncharacterized protein	0,00010	0,32048	0,00059	1,42244	0,00023	1,61947
111	A0A6N0BMC3	S-adenosylmethionine synthase	0,67344	0,94848	0,00049	2,28498	0,01501	1,60500
112	A0A6N0B8G8	D-ribose-binding periplasmic protein	0,16748	1,06062	0,02773	1,11440	0,00240	1,14397

113	A0A6N0BKQ0	3-isopropylmalate dehydratase large subunit 1	0,00687	1,38201	0,00393	0,70050	0,00029	0,47409
114	A0A6N0AT44	Chemotaxis protein CheA	0,00233	0,42265	0,01132	0,70904	0,32893	0,78578
115	A0A6N0AJK7	Aspartate-semialdehyde dehydrogenase	0,62515	1,03737	0,60846	1,03747	0,55667	1,04295
116	A0A6N0BI02	50S ribosomal protein L9	0,01030	0,75023	0,00001	0,66949	0,33152	0,91933
117	A0A6N0ACI6	Ribose-phosphate pyrophosphokinase	0,02914	1,23361	0,44651	1,06454	0,68362	1,01936
118	A0A6N0AVE9	50S ribosomal protein L6	0,18458	1,07789	0,98320	0,99897	0,68120	0,98855
119	A0A6N0AIX9	ATP synthase gamma chain	0,18175	1,15152	0,79846	1,02537	0,28453	0,97072
120	A0A6N0AUZ6	Putative ABC transporter ATP-binding protein YjjK	0,14878	0,75301	0,11529	1,13726	0,59354	1,14336
121	A0A6N0BJ85	50S ribosomal protein L3	0,16066	0,78129	0,07413	1,14182	0,22334	1,24230
122	A0A6N0BAD7	Glutamine--fructose-6-phosphate aminotransferase [isomerizing]	0,00155	1,80829	0,09976	1,23133	0,00848	0,82714
123	A0A6N0BHV4	CTP synthase	0,10508	1,15140	0,38465	0,94040	0,17456	0,92974
124	A0A6N0AP75	NAD-dependent malic enzyme	0,02024	3,09957	0,03788	2,45248	0,61164	0,88963
125	A0A6N0AGK2	NADH-quinone oxidoreductase subunit G	0,30360	0,84142	0,77368	1,04990	0,68726	1,04640
126	A0A6N0AIZ0	50S ribosomal protein L7/L12	0,00158	0,55146	0,01018	0,76084	0,00001	2,71550
127	A0A6N0A8Q4	Glutamate synthase [NADPH] small chain	0,04673	1,36496	0,11049	1,49253	0,00136	1,29921
128	A0A6N0AH41	NADH-quinone oxidoreductase subunit F	0,30614	1,11281	0,00506	1,27143	0,85351	0,97419
129	A0A6N0BC61	Phosphoenolpyruvate-protein phosphotransferase	0,11056	1,37899	0,86935	0,96669	0,63219	0,96347
130	A0A6N0AVI0	Cytosol aminopeptidase	0,00016	1,77607	0,00025	1,41170	0,25674	0,91272
131	A0A6N0A780	Glycine--tRNA ligase beta subunit	0,87899	1,01323	0,05134	0,77444	0,06769	0,87104
132	A0A6N0ADU3	Aspartate aminotransferase	0,04569	0,75356	0,19717	0,89211	0,12826	1,21026
133	A0A6N0ARF6	DNA-binding protein H-NS	0,89105	1,02341	0,00443	1,93670	0,15101	1,28151
134	A0A6N0BHK2	NADH-quinone oxidoreductase subunit C/D	0,38339	0,92753	0,14156	0,87943	0,75200	0,97441
135	A0A6N0AQQ9	Aspartate--tRNA ligase	0,00176	1,54960	0,02138	1,26185	0,01144	0,78918
136	A0A6N0AGF3	Isoleucine--tRNA ligase	0,42774	1,20844	0,44557	1,11031	0,83767	0,95070
137	A0A6N0B6R0	Acetolactate synthase isozyme 3 large subunit	0,93246	0,98853	0,02686	1,44282	0,00937	1,38723
138	A0A6N0AQA3	Outer membrane protein assembly factor BamC	0,54176	1,17687	0,69386	1,08122	0,60001	1,09683
139	A0A6N0BBD8	ATP-dependent Clp protease ATP-binding subunit ClpX	0,00909	1,23894	0,22122	0,92420	0,55723	0,96277
140	A0A6N0ADG0	Glucans biosynthesis protein G	0,38281	1,06553	0,69328	1,05221	0,59190	1,07151
141	A0A6N0BJY9	50S ribosomal protein L24	0,00165	0,84972	0,00001	0,49672	0,00499	0,81580
142	A0A6N0AGF6	Vitamin B12-binding protein	0,08251	1,42476	0,09938	1,28647	0,36267	1,14031

143	A0A6N0APT0	Thiol peroxidase	0,00220	1,21523	0,00517	1,14702	0,00316	1,15501
144	A0A6N0AVX4	Dihydroxy-acid dehydratase	0,08605	1,24088	0,17541	0,82256	0,23017	1,06208
145	A0A6N0AUB8	Enoyl-[acyl-carrier-protein] reductase [NADH] FabI	0,95038	0,98701	0,89647	1,02722	0,79567	1,02202
146	A0A6N0A9G1	Ribonucleoside-diphosphate reductase 1 subunit alpha	0,28324	1,26337	0,09176	1,30759	0,27679	1,16384
147	A0A6N0AJ33	Proline--tRNA ligase	0,05086	1,68743	0,08670	1,55980	0,99784	1,00019
148	A0A6N0ADF0	Uncharacterized protein	0,07308	1,38604	0,03804	1,35517	0,22906	1,14525
149	A0A6N0AHU9	Periplasmic dipeptide transport protein	0,04627	0,62017	0,01243	0,78548	0,34358	0,72973
150	A0A6N0AKM9	ATP synthase subunit b	0,93056	1,00765	0,00914	1,22597	0,44548	1,08703
151	A0A6N0AAE7	Phosphoglucomutase	0,00893	6,82999	0,02750	2,50826	0,52022	0,83779
152	A0A6N0B8V3	Acetyl-coenzyme A synthetase	0,20880	0,81456	0,51612	1,08114	0,36529	1,15407
153	A0A6N0BHM4	Lysine-arginine-ornithine-binding periplasmic protein	0,78280	0,98614	0,00590	1,23065	0,12754	1,10584
154	A0A6N0B8P1	Triosephosphate isomerase	0,02054	0,85403	0,00143	0,72176	0,35434	0,95590
155	A0A6N0AA80	DNA-binding protein HU-alpha	0,03730	1,28692	0,00002	1,72754	0,03567	1,25249
156	A0A6N0AK18	Modulator of FtsH protease HflK	0,45499	1,17333	0,27400	1,13046	0,90075	1,03018
157	A0A6N0AIH5	Cell division protein FtsZ	0,18802	1,15331	0,82343	0,97702	0,00622	0,85238
158	A0A6N0BIG4	30S ribosomal protein S7	0,53565	0,89854	0,08952	0,86867	0,34370	0,83280
159	A0A6N0A9K2	Succinyl-CoA ligase [ADP-forming] subunit alpha	0,87262	1,01425	0,00006	1,61908	0,19235	1,28157
160	A0A6N0BJT9	Bifunctional polymyxin resistance protein ArnA	0,49026	1,35171	0,08111	1,64055	0,01286	2,43347
161	A0A6N0ABF6	Serine--tRNA ligase	0,21140	1,17374	0,01173	1,51432	0,01302	1,22363
162	A0A6N0ANX1	Phosphoribosylaminoimidazole-succinocarboxamide synthase	0,17513	1,15684	0,01592	1,34686	0,03403	1,18103
163	A0A6N0BC06	Succinate dehydrogenase iron-sulfur subunit	0,14507	1,05848	0,44514	0,97004	0,03558	1,07430
164	A0A6N0AWV6	GTP-binding protein TypA/BipA	0,00122	0,49289	0,00422	0,63224	0,61466	1,11191
165	A0A6N0AGQ9	FKBP-type peptidyl-prolyl cis-trans isomerase FkpA	0,00726	1,59034	0,05061	1,79650	0,02855	1,33710
166	A0A6N0ARS9	Protein RecA	0,00007	2,58795	0,20971	0,92234	0,84179	0,98839
167	A0A6N0BE90	NH(3)-dependent NAD(+) synthetase	0,14498	0,93096	0,01018	1,15752	0,00124	1,21282
168	A0A6N0BFR0	3-oxoacyl-[acyl-carrier-protein] reductase FabG	0,81181	0,96513	0,28644	1,18134	0,17850	1,07167
169	A0A6N0BBS5	Outer membrane protein X	0,00011	2,05299	0,00107	0,85199	0,33524	0,85741
170	A0A6N0BGF8	Methyl-accepting chemotaxis protein III	0,01701	0,55486	0,00780	1,50002	0,10479	1,29222
171	A0A6N0ATH2	Acetylornithine/succinyldiaminopimelate aminotransferase	0,94215	1,01278	0,00583	1,86909	0,02357	1,68765
172	A0A6N0A987	Phosphoenolpyruvate carboxylase	0,82345	0,93442	0,13961	0,64939	0,35563	0,76075

173	A0A6N0A9K7	Protein TolB	0,00195	1,36654	0,43587	1,04109	0,95664	0,99148
174	A0A6N0AL00	3-carboxy-cis,cis-muconate cycloisomerase	0,00023	2,10515	0,00085	1,70898	0,98693	1,00068
175	A0A6N0AD51	Leucine--tRNA ligase	0,12871	1,26771	0,00101	1,70612	0,08611	1,24753
176	A0A6N0B112	Septum site-determining protein MinD	0,04466	1,20527	0,01126	1,26430	0,23157	1,07170
177	A0A6N0B8N1	ATP-dependent protease ATPase subunit HslU	0,48363	1,11211	0,11927	1,22395	0,83118	1,02268
178	A0A6N0ACH9	Iron uptake system component EfeO	0,29980	1,13324	0,07466	1,26479	0,00003	1,66653
179	A0A6N0BJB5	GMP synthase [glutamine-hydrolyzing]	0,02991	1,46498	0,11597	0,74718	0,02580	0,65935
180	A0A6N0AN25	Valine--tRNA ligase	0,00070	1,54511	0,25969	0,80381	0,51181	0,96807
181	A0A6N0BHD6	50S ribosomal protein L22	0,19692	0,41727	0,49453	0,67285	0,54923	1,42038
182	A0A6N0AE52	Adenylosuccinate lyase	0,21954	1,13356	0,21734	1,14759	0,55907	0,94563
183	A0A6N0B0A8	Methionine--tRNA ligase	0,00542	1,16542	0,00581	1,28763	0,15048	1,08505
184	A0A6N0BHX4	Bifunctional protein HldE	0,49347	1,11489	0,34528	0,87015	0,43545	0,90602
185	A0A6N0ATA7	30S ribosomal protein S8	0,00449	0,77782	0,05416	0,89833	0,06656	0,91678
186	A0A6N0ATJ6	PTS system mannose-specific EIIAB component	0,14019	0,81149	0,02986	1,27265	0,50569	1,06041
187	A0A6N0AGH9	Aminopeptidase N	0,00577	1,51532	0,00004	1,50452	0,16910	1,11893
188	A0A6N0AYC1	Peptidoglycan-associated lipoprotein	0,72460	0,94364	0,43694	1,07333	0,54125	1,13962
189	A0A6N0BGD8	Oligogalacturonate-specific porin KdgM	0,05859	0,84589	0,52674	0,96292	0,52290	0,95716
190	A0A6N0AMP5	Phosphoribosylamine--glycine ligase	0,34794	0,91416	0,53559	1,09379	0,08163	1,24969
191	A0A6N0ASG4	Acetate kinase	0,13011	1,22230	0,01469	0,83069	0,11799	0,79491
192	A0A6N0BKC3	30S ribosomal protein S6	0,95554	0,98659	0,00868	1,93944	0,02357	1,92193
193	A0A6N0ALR6	UTP--glucose-1-phosphate uridylyltransferase	0,00280	1,78642	0,04903	1,30902	0,19791	0,93296
194	A0A6N0ANC0	L-glyceraldehyde 3-phosphate reductase	0,87908	0,96875	0,97844	0,99430	0,00816	1,12839
195	A0A6N0AXG4	Acetyl-coenzyme A carboxylase carboxyl transferase subunit alpha	0,00049	2,25602	0,00111	2,10096	0,64222	0,96979
196	A0A6N0A9J2	Uracil phosphoribosyltransferase	0,00053	0,73527	0,00009	0,71031	0,03994	0,88703
197	A0A6N0BMC4	50S ribosomal protein L16	0,09190	0,92422	0,33150	0,97142	0,56482	0,94685
198	A0A6N0BJ16	Fumarate reductase flavoprotein subunit	0,07855	0,86684	0,01000	1,13649	0,09468	0,81652
199	A0A6N0AIJ2	3-isopropylmalate dehydrogenase	0,00522	1,47449	0,24436	1,12508	0,15700	0,89960
200	A0A6N0B8Q9	Bifunctional purine biosynthesis protein PurH	0,04973	0,73462	0,00657	1,25545	0,27046	1,22485
201	A0A6N0AFU0	2-dehydro-3-deoxyphosphooctonate aldolase	0,02414	1,14145	0,00184	0,73617	0,00295	0,81921
202	A0A6N0AXT5	Modulator of FtsH protease HflC	0,00014	1,81509	0,00012	1,69184	0,57805	1,04170

203	A0A6N0ART2	Asparagine--tRNA ligase	0,29926	0,89496	0,01624	0,62811	0,07505	0,64304
204	A0A6N0AJL8	Tryptophan--tRNA ligase	0,00241	2,11444	0,00026	2,28868	0,86050	1,02191
205	A0A6N0AV63	Argininosuccinate lyase	0,42595	1,13707	0,11193	1,27076	0,13275	1,15473
206	A0A6N0B620	Sulfite reductase [NADPH] flavoprotein alpha-component	0,48103	1,14628	0,03766	2,05943	0,66869	1,04095
207	A0A6N0AIU7	Carbamoyl-phosphate synthase small chain	0,94534	0,99339	0,01299	1,37793	0,00457	1,19584
208	A0A6N0AZR2	Glucose-specific phosphotransferase enzyme IIA component	0,13567	1,07311	0,00039	1,37087	0,10077	1,17610
209	A0A6N0BDS3	ATP phosphoribosyltransferase	0,47438	1,12213	0,76256	0,95915	0,35438	0,89686
210	A0A6N0BBU3	4-hydroxy-tetrahydrodipicolinate synthase	0,42084	1,09454	0,81022	1,02318	0,19514	1,10590
211	A0A6N0BH32	Glutathione synthetase	0,00425	2,72790	0,00124	3,09581	0,11849	1,23026
212	A0A6N0AD36	UDP-glucose 6-dehydrogenase	0,03981	1,19972	0,01598	0,87347	0,06114	0,82502
213	A0A6N0ASJ1	Thioredoxin reductase	0,92612	0,98832	0,17289	1,16114	0,04865	1,26851
214	A0A2K8VY47	Chaperone SurA	0,09174	1,62416	0,00981	2,08602	0,16463	1,20056
215	A0A6N0BMB3	30S ribosomal protein S5	0,00381	0,84773	0,00005	0,61691	0,03784	0,88806
216	A0A6N0AZK5	Adenylate kinase	0,22315	0,81546	0,23345	1,10635	0,39516	1,15842
217	A0A6N0B7M5	ATP synthase subunit delta	0,08974	0,66323	0,24625	0,81989	0,19453	1,24509
218	A0A6N0B632	General stress protein 69	0,05642	2,00076	0,00095	3,62952	0,39277	1,12280
219	A0A6N0AIR2	2-isopropylmalate synthase	0,03569	0,40261	0,25693	0,70109	0,16289	1,34601
220	A0A6N0B2W0	Uncharacterized protein	0,01626	0,65854	0,00237	1,53704	0,60770	1,04742
221	A0A6N0AYR4	Catalase-peroxidase	0,81085	1,05364	0,00173	2,08803	0,00139	2,36121
222	A0A6N0ARE9	NAD(P)H nitroreductase YdjA	0,79186	1,04220	0,71542	0,95247	0,21593	1,18799
223	A0A6N0ACQ0	Peroxiredoxin bcp	0,00268	1,21773	0,00019	0,74491	0,00255	0,79051
224	A0A6N0AF95	Periplasmic serine endoprotease DegP	0,01620	2,99824	0,40622	1,35366	0,33500	1,20336
225	A0A6N0B5N4	30S ribosomal protein S10	0,00221	0,59315	0,00048	0,44698	0,15513	0,90541
226	A0A6N0ADU6	Formate dehydrogenase-O major subunit	0,55537	1,11916	0,67061	0,96738	0,24921	0,78161
227	A0A6N0AUS8	Aerobic respiration control protein ArcA	0,41700	1,12448	0,11442	1,21446	0,79673	1,04022
228	A0A6N0B672	Outer membrane protein assembly factor BamB	0,32982	1,22159	0,06602	1,39820	0,79626	1,04687
229	A0A6N0AT12	Peptidase B	0,29493	1,09851	0,44429	0,94080	0,69280	1,02247
230	A0A6N0AI34	Inorganic pyrophosphatase	0,03444	0,85660	0,00011	0,29872	0,00014	0,57573
231	A0A6N0AMS6	Cytochrome bd-I ubiquinol oxidase subunit 1	0,05609	0,77266	0,04471	0,76401	0,00230	0,64370
232	A0A6N0B8W4	PTS system mannitol-specific EIICBA component	0,70740	0,92412	0,93537	0,98779	0,10172	1,36425

233	A0A6N0B1Z5	Major phosphate-irrepressible acid phosphatase	0,00592	0,38262	0,31226	1,13501	0,05373	0,85841
234	A0A6N0BBV4	Cold shock-like protein CspE	0,00547	1,28486	0,00514	0,43848	0,01803	0,68921
235	A0A6N0ASX1	Biosynthetic arginine decarboxylase	0,00101	3,15235	0,01024	2,57119	0,34586	1,12798
236	A0A6N0B974	6-phosphofructokinase isozyme 1	0,86768	0,97300	0,68738	1,05954	0,69011	1,04826
237	A0A6N0AFS3	Protein translocase subunit SecA	0,03114	1,64354	0,49198	1,24558	0,12037	0,59716
238	A0A6N0AL83	DNA gyrase subunit A	0,87042	1,01848	0,00052	1,31615	0,18580	1,18107
239	A0A6N0BD39	Thioredoxin	0,33217	1,27276	0,00665	2,15078	0,18546	1,42257
240	A0A6N0B2Y4	Ferrichrome-iron receptor	0,79197	0,81545	0,59793	1,29402	0,57809	0,66083
241	A0A6N0AT47	Glucose-6-phosphate isomerase	0,50373	1,18239	0,55603	1,16898	0,65603	1,02669
242	A0A6N0AFN3	Phage shock protein A	0,39875	0,88745	0,00122	1,28996	0,19296	0,79299
243	A0A6N0AW53	Uncharacterized protein	0,02273	0,75267	0,04224	0,86806	0,83497	0,97366
244	A0A6N0ACU7	Mannose-6-phosphate isomerase	0,00525	1,38851	0,33684	0,85623	0,07591	0,79836
245	A0A6N0BDC0	50S ribosomal protein L25	0,47301	0,85924	0,15213	0,69211	0,14539	0,72862
246	A0A6N0BM05	Vitamin B12 transporter BtuB	0,14167	1,37450	0,02208	1,66146	0,90763	1,01195
247	A0A6N0A805	Purine nucleoside phosphorylase DeoD-type	0,05193	1,50957	0,47988	1,10806	0,17724	0,84939
248	A0A6N0ANT6	Uncharacterized protein	0,18955	0,83425	0,01224	1,30696	0,48306	1,09427
249	A0A6N0AKT8	2,3,4,5-tetrahydropyridine-2,6-dicarboxylate N-succinyltransferase	0,90502	1,00663	0,00042	0,66875	0,03371	0,84047
250	A0A6N0B7X5	5,10-methylenetetrahydrofolate reductase	0,01395	0,77516	0,34063	0,93957	0,46749	1,06398
251	A0A6N0AY12	10 kDa chaperonin	0,00037	0,29591	0,17639	0,63569	0,67357	0,90979
252	A0A6N0BJ22	50S ribosomal protein L15	0,01670	0,66411	0,03250	0,72944	0,53719	0,92992
253	A0A6N0B9G6	Alcohol dehydrogenase YqhD	0,74984	1,05683	0,18268	0,73416	0,07246	0,85609
254	A0A6N0ANV8	Phosphoribosylformylglycinamidine cyclo-ligase	0,53027	1,07468	0,66182	1,05702	0,93620	1,01848
255	A0A6N0BHK3	Sulfate adenylyltransferase subunit 1	0,01356	0,71651	0,06411	1,14082	0,05415	1,32979
256	A0A6N0AXL0	Multidrug efflux pump subunit AcrA	0,85192	1,03523	0,41112	1,09646	0,47490	1,11640
257	A0A6N0BIX2	Branched-chain-amino-acid aminotransferase	0,37441	1,29858	0,55869	1,14557	0,22370	1,23064
258	A0A6N0AGS8	S-ribosylhomocysteine lyase	0,10636	0,81471	0,05666	0,78869	0,29340	0,90991
259	A0A6N0AUW7	Acetylglutamate kinase	0,00165	1,30130	0,01483	1,24583	0,15106	1,12632
260	A0A6N0BK33	cAMP-activated global transcriptional regulator CRP	0,87223	0,96544	0,00687	1,92157	0,79252	1,06351
261	A0A6N0AM80	50S ribosomal protein L21	0,08443	0,65579	0,03294	1,46144	0,14862	1,50726
262	A0A6N0AHP1	Nucleoside diphosphate kinase	0,63086	0,98861	0,01155	0,86966	0,87861	0,99633

263	A0A6N0AVN6	Transcriptional regulatory protein OmpR	0,35850	1,35801	0,09040	1,74402	0,83084	1,05734
264	A0A6N0AIR5	Osmotically-inducible protein Y	0,97942	1,00530	0,01130	1,53776	0,33962	1,27210
265	A0A6N0BBU2	Periplasmic dipeptide transport protein	0,25167	0,75194	0,84125	0,97160	0,90259	1,04662
266	A0A6N0BIQ6	Phosphomannomutase/phosphoglucomutase	0,95992	1,01884	0,85070	0,93325	0,22309	0,76420
267	A0A2K8VTD8	Arginine--tRNA ligase	0,43171	0,82034	0,54612	0,86788	0,66819	1,04625
268	A0A6N0A915	N-acetyl-gamma-glutamyl-phosphate reductase	0,35035	1,09517	0,01305	0,66365	0,03021	0,83117
269	A0A6N0ABD6	ABC transporter arginine-binding protein 2	0,02738	1,75284	0,01916	1,78976	0,78198	0,93334
270	A0A6N0BCX3	DNA-binding protein HU-beta	0,00261	0,77552	0,93319	0,99702	0,00208	1,20346
271	A0A6N0B633	Aromatic-amino-acid aminotransferase	0,74768	0,89054	0,47584	0,77675	0,85661	0,95324
272	A0A6N0B8P5	Aldehyde dehydrogenase B	0,39990	0,78288	0,04417	1,47417	0,16280	1,36328
273	A0A6N0A8I6	NADP-dependent malic enzyme	0,00647	1,71556	0,00531	1,86478	0,10140	1,24973
274	A0A6N0ABY0	Tryptophan synthase beta chain	0,80885	0,95175	0,00069	1,63933	0,01825	1,73193
275	A0A6N0AXE2	Uncharacterized protein	0,37238	1,12933	0,02771	1,40840	0,19016	1,14424
276	A0A6N0AXS3	Uncharacterized protein	0,00257	1,54018	0,04429	1,13839	0,03318	0,80229
277	A0A6N0B7Y6	Lysine-sensitive aspartokinase 3	0,11165	0,54689	0,14430	0,67542	0,51312	1,20671
278	A0A6N0BI12	Ribonuclease R	0,00305	1,41558	0,07805	1,16275	0,29617	0,86668
279	A0A6N0A802	UDP-N-acetylglucosamine 1-carboxyvinyltransferase	0,13211	0,77512	0,05875	0,73883	0,29917	1,11498
280	A0A6N0A6E0	Superoxide dismutase [Mn]	0,60241	1,10087	0,00003	3,08577	0,00341	2,03166
281	A0A6N0B030	Ribosome-recycling factor	0,46159	0,88050	0,51066	0,85542	0,47639	0,79630
282	A0A6N0AIT6	Thioredoxin-1	0,01872	1,17953	0,06009	0,86866	0,03492	0,93058
283	A0A6N0AKI9	DNA-directed RNA polymerase subunit omega	0,33462	0,92559	0,01100	0,73816	0,37625	0,94934
284	A0A6N0B8S8	Biotin carboxylase	0,05624	1,10176	0,92467	1,00342	0,21748	0,90472
285	A0A6N0AF74	Chemotaxis protein CheY	0,02714	0,58608	0,47452	1,12170	0,14316	1,14242
286	A0A6N0B0S2	Tryptophan synthase alpha chain	0,14027	0,80617	0,00393	1,26934	0,01725	1,55037
287	A0A6N0AN16	DNA protection during starvation protein	0,71186	1,14148	0,00098	6,55255	0,00009	5,62736
288	A0A6N0AG39	3-oxoacyl-[acyl-carrier-protein] synthase 3	0,00144	1,63160	0,29006	1,03877	0,85496	0,97814
289	A0A6N0AWS8	Universal stress protein A	0,12041	1,35839	0,00927	1,53533	0,64069	0,91072
290	A0A6N0AWP5	Cystathionine gamma-synthase	0,44825	0,96244	0,04351	0,83609	0,17934	1,03915
291	A0A6N0BAH9	Ribonuclease PH	0,91185	1,02173	0,04343	1,40748	0,08217	1,40464
292	A0A6N0AL50	6,7-dimethyl-8-ribityllumazine synthase	0,54941	0,84695	0,14899	0,57854	0,41282	1,06019
293	A0A6N0AB00	Adenine phosphoribosyltransferase	0,18675	0,80964	0,02080	0,44201	0,34452	1,08326

294	A0A6N0A9Y2	Alkyl hydroperoxide reductase subunit C	0,15091	0,66837	0,00003	4,76460	0,00004	6,35725
295	A0A6N0BG99	Nucleoid-associated protein YejK	0,79867	0,88525	0,37643	0,61160	0,63207	0,83854
296	A0A6N0BAC9	Ornithine carbamoyltransferase chain I	0,91080	0,98556	0,03206	1,38560	0,27464	1,18672
297	A0A6N0B602	Peptidyl-prolyl cis-trans isomerase B	0,14730	0,74047	0,80296	1,04514	0,00419	1,84478
298	A0A6N0B689	30S ribosomal protein S16	0,00006	0,87281	0,00000	0,66704	0,00011	0,84841
299	A0A6N0AVB9	30S ribosomal protein S13	0,31258	0,76941	0,00399	1,49486	0,50469	1,23091
300	A0A2K8VYT1	30S ribosomal protein S11	0,78816	0,97621	0,00039	1,66363	0,00658	1,52169
301	A0A6N0A7R6	50S ribosomal protein L11	0,27417	1,31732	0,78728	1,07185	0,02421	0,88459
302	A0A6N0AWC1	Autonomous glycyl radical cofactor	0,51906	0,86012	0,23906	0,75361	0,17838	0,74546
303	A0A6N0ATK8	Cysteine desulfurase	0,35327	0,92018	0,08733	1,15573	0,83314	1,02813
304	A0A6N0BKH3	Phospho-2-dehydro-3-deoxyheptonate aldolase, Tyr-sensitive	0,50823	1,06410	0,57043	1,06282	0,81632	0,97736
305	A0A6N0BFC9	ABC transporter substrate-binding protein YesO	0,81287	1,05089	0,00571	1,82098	0,45034	0,83952
306	A0A6N0AS38	NADH-quinone oxidoreductase subunit B	0,07001	0,84364	0,26359	1,10733	0,19285	1,10974
307	A0A6N0BHZ7	D-3-phosphoglycerate dehydrogenase	0,17212	1,47488	0,33112	1,25421	0,49995	1,12719
308	A0A6N0BFC7	Vitamin B12-binding protein	0,87745	1,11560	0,43346	0,65024	0,32300	1,87950
309	A0A6N0AR21	Leucine-responsive regulatory protein	0,01911	0,82032	0,00096	1,44068	0,04963	1,16110
310	A0A6N0AMQ6	D-alanyl-D-alanine carboxypeptidase DacA	0,97315	0,99390	0,01772	0,67484	0,58737	0,89097
311	A0A6N0AK79	Uncharacterized protein	0,01255	2,34919	0,00199	3,00759	0,00291	1,42318
312	A0A6N0BAA2	Lysine--tRNA ligase	0,20847	0,70422	0,09128	1,25641	0,11165	1,53736
313	A0A6N0APD8	Fumarate hydratase class I, aerobic	0,21047	0,90527	0,25661	1,21818	0,33170	0,79381
314	A0A6N0BA28	Membrane protein insertase YidC	0,02257	1,30162	0,00396	1,21932	0,36701	1,06496
315	A0A6N0AU66	Glutamate--tRNA ligase	0,99956	0,99984	0,38288	1,12188	0,53248	1,16356
316	A0A6N0B047	Multidrug efflux pump subunit AcrB	0,91311	0,98421	0,62177	0,95695	0,39293	1,12737
317	A0A6N0AZU4	D-methionine-binding lipoprotein MetQ	0,59882	1,14580	0,05637	1,63864	0,19400	1,15693
318	A0A6N0AGA6	Uncharacterized protein	0,04511	0,84156	0,15099	0,88621	0,31275	0,96300
319	A0A6N0AI45	Putative hydrolase YxeP	0,00133	3,36004	0,00100	3,30366	0,89179	0,97234
320	A0A6N0ARC4	Carbohydrate acetyl esterase/feruloyl esterase	0,62476	1,16962	0,00764	2,40469	0,73487	1,09410
321	A0A6N0BEP3	Hydrogen peroxide-inducible genes activator	0,08469	1,32940	0,34084	1,17081	0,28734	1,10583
322	A0A6N0BEI8	Chaperone protein Skp	0,56132	1,08634	0,41285	1,12048	0,01264	1,19153
323	A0A6N0AFG9	30S ribosomal protein S18	0,02131	0,79457	0,01235	0,80197	0,98759	0,99871

324	A0A6N0BK11	USG-1 protein	0,14420	1,93566	0,17818	1,29821	0,15886	0,51150
325	A0A6N0B6G0	Cytosol non-specific dipeptidase	0,01472	1,91633	0,08910	1,35383	0,62525	1,13157
326	A0A6N0B675	FKBP-type 22 kDa peptidyl-prolyl cis-trans isomerase	0,97712	1,00595	0,00448	1,30077	0,13750	1,38382
327	A0A6N0A9L8	Cation efflux system protein CusB	0,18276	1,14122	0,71205	1,05367	0,01514	0,85456
328	A0A6N0B1Z6	Dihydroorotate dehydrogenase (Quinone)	0,40745	0,76547	0,98304	1,00456	0,35401	1,29040
329	A0A6N0AH46	Chemotaxis protein CheW	0,02691	0,44510	0,92289	1,00914	0,40672	1,28596
330	A0A6N0AVD9	50S ribosomal protein L23	0,02001	0,87243	0,00031	0,66029	0,02169	0,81983
331	A0A6N0ADA5	Threonine--tRNA ligase	0,75877	0,92254	0,03685	1,40713	0,82398	1,06373
332	A0A6N0BI00	Transcriptional regulatory protein YycF	0,03727	1,12150	0,00010	0,63911	0,00103	0,71601
333	A0A6N0B2Q9	Ribosome-binding ATPase YchF	0,72997	1,06465	0,01242	1,56206	0,06797	1,37402
334	A0A6N0ARF4	UTP--glucose-1-phosphate uridylyltransferase	0,16965	1,14980	0,06764	0,94609	0,19876	0,83164
335	A0A6N0ADU5	Universal stress protein E	0,29578	0,76989	0,03203	0,41925	0,62262	0,92356
336	A0A6N0ADF1	Dihydroorotase	0,80795	1,13771	0,08242	2,20790	0,05514	1,57906
337	A0A6N0B1S2	S-(Hydroxymethyl)glutathione dehydrogenase	0,87075	0,94516	0,61583	0,83357	0,58528	1,10143
338	A0A6N0BHM5	Ribosome-associated inhibitor A	0,00030	1,72749	0,00006	2,05232	0,00258	1,21412
339	A0A6N0B1V3	NADPH dehydrogenase	0,13922	1,36783	0,03122	0,41770	0,03482	0,67762
340	A0A6N0AYU5	Regulator of ribonuclease activity A	0,41564	1,62280	0,81953	0,88822	0,93473	1,03075
341	A0A6N0AJ31	DNA gyrase subunit B	0,34967	1,49905	0,25479	1,82079	0,19085	1,37894
342	A0A6N0B6D3	L-aspartate oxidase	0,39059	0,92389	0,00914	0,65515	0,28697	0,87750
343	A0A6N0BBM6	50S ribosomal protein L13	0,15042	0,75988	0,12733	1,06745	0,22522	1,27016
344	A0A6N0AIP1	Chaperone protein DnaJ	0,04031	1,35819	0,00483	1,69001	0,00329	1,22802
345	A0A6N0ATU8	ATP-dependent RNA helicase RhlB	0,05389	1,42659	0,00217	1,99842	0,03240	1,30052
346	A0A6N0A9T3	Glyoxylate/hydroxypyruvate reductase B	0,91290	0,94919	0,05213	1,84229	0,32774	1,67296
347	A0A6N0A6N3	Protein-export protein SecB	0,74080	1,14609	0,02348	1,83527	0,01388	2,28091
348	A0A6N0B819	Fructose-1,6-bisphosphatase class 1	0,01228	1,41882	0,61096	0,93789	0,72409	0,95829
349	A0A6N0ASW1	Ribose-5-phosphate isomerase A	0,66075	1,12546	0,12612	1,29415	0,75598	0,85831
350	A0A6N0AH49	Uncharacterized protein	0,82852	1,06894	0,00320	2,65606	0,77669	1,10074
351	A0A6N0AF66	Phenylalanine--tRNA ligase alpha subunit	0,80580	0,77742	0,14286	2,16127	0,38854	2,25381
352	A0A6N0AY01	Mandelate racemase	0,68371	0,95375	0,00299	0,85355	0,60902	0,92849
353	A0A6N0AYD1	Uncharacterized protein	0,32312	1,29511	0,36599	1,19790	0,84087	1,03107
354	A0A6N0BHB7	50S ribosomal protein L17	0,00037	0,88528	0,00107	0,70766	0,00006	0,83591

355	A0A6N0ADJ5	3-oxoacyl-[acyl-carrier-protein] synthase 2	0,25138	0,90686	0,00492	1,32536	0,00657	1,44036
356	A0A6N0ADH1	Hydroperoxy fatty acid reductase gpx1	0,63042	1,04936	0,29357	0,89484	0,17740	0,87409
357	A0A6N0BEQ9	Acetyl-coenzyme A carboxylase carboxyl transferase subunit beta	0,15456	0,68085	0,01367	0,60784	0,78951	0,91222
358	A0A6N0AIG5	5'-methylthioadenosine/S-adenosylhomocysteine nucleosidase	0,10040	1,25287	0,00964	1,45341	0,04459	1,25800
359	A0A6N0BA21	Glutamine--tRNA ligase	0,98083	1,00443	0,01866	1,42486	0,56405	1,11228
360	A0A6N0AQ52	50S ribosomal protein L32	0,02939	0,46988	0,04731	0,56738	0,42621	1,37839
361	A0A6N0ABU4	Glucose-6-phosphate 1-epimerase	0,14066	1,46370	0,04757	1,62574	0,31858	1,19355
362	A0A6N0BIF9	30S ribosomal protein S19	0,40715	1,18166	0,46672	0,82974	0,72534	1,05070
363	A0A6N0BFA8	Cytoskeleton protein RodZ	0,79101	0,93160	0,34487	0,81555	0,83943	1,05655
364	A0A6N0B7R8	Uroporphyrinogen-III C-methyltransferase	0,00720	1,57176	0,03246	1,32131	0,25457	0,93686
365	A0A6N0AIQ3	UDP-N-acetylmuramoyl-L-alanyl-D-glutamate--2, 6-diaminopimelate ligase	0,41966	0,67305	0,66795	0,83926	0,22633	1,24588
366	A0A6N0BE41	Uncharacterized protein	0,21779	1,21775	0,66065	1,07522	0,10172	0,84411
367	A0A6N0B3P2	Ureidoglycolate lyase	0,35003	0,84727	0,12329	0,73039	0,65995	0,95956
368	A0A6N0BKZ5	Uncharacterized protein	0,72246	1,04954	0,13224	1,24089	0,26011	0,91010
369	A0A6N0A900	ADP-L-glycero-D-manno-heptose-6-epimerase	0,00643	1,27977	0,00597	1,33653	0,07261	1,07213
370	A0A6N0BH40	RNA-binding protein Hfq	0,53402	0,87615	0,13801	0,74459	0,86303	1,03400
371	A0A6N0AXS8	Cytochrome bo(3) ubiquinol oxidase subunit 2	0,65117	0,84833	0,48385	1,21110	0,35265	1,33936
372	A0A6N0AJ43	Enamine/imine deaminase	0,83796	1,08177	0,65993	1,22671	0,39270	1,28965
373	A0A6N0BDZ1	NAD(P) transhydrogenase subunit alpha	0,96766	0,98341	0,01803	0,55989	0,41855	1,61590
374	A0A6N0B5Q4	3-isopropylmalate dehydratase small subunit 1	0,62497	1,04477	0,08668	0,58843	0,00125	0,48681
375	A0A6N0AF63	1-(5-phosphoribosyl)-5-[(5-phosphoribosylamino)methylideneamino] imidazole-4-carboxamide isomerase	0,65646	1,10665	0,04000	1,51585	0,01122	1,63180
376	A0A6N0B491	Aerotaxis receptor	0,43281	1,38242	0,90689	1,05439	0,88038	0,96975
377	A0A6N0AT52	Soluble cytochrome b562	0,08392	1,19495	0,00274	0,77545	0,27810	1,14425
378	A0A6N0AT35	DNA topoisomerase 1	0,07408	1,77409	0,02448	1,89071	0,72562	0,95037
379	A0A6N0AG11	Tail-specific protease	0,82696	0,94532	0,01174	1,31214	0,17397	1,39812
380	A0A6N0B091	1,4-Dihydroxy-2-naphthoyl-CoA synthase	0,01859	1,40543	0,00017	2,05315	0,30975	1,12996

381	A0A6N0B404	Enolase-phosphatase E1	0,34736	1,35522	0,64489	1,14188	0,72770	1,06306
382	A0A6N0B1Y5	Imidazole glycerol phosphate synthase subunit HisF	0,04466	1,10620	0,00251	0,72396	0,04315	0,83279
383	A0A6N0AMJ7	Phosphopentomutase	0,06539	1,50755	0,89339	0,98174	0,24806	0,63957
384	A0A6N0AJ09	Cystine-binding periplasmic protein	0,84782	1,07388	0,81914	1,06621	0,35725	1,27744
385	A0A6N0AKD8	Thiol:disulfide interchange protein DsbA	0,00366	0,03661	0,11067	1,34972	0,15995	1,75839
386	A0A6N0B884	Small heat shock protein IbpB	0,00386	0,75308	0,00000	0,38764	0,00082	0,51344
387	A0A6N0BHM3	Exoribonuclease 2	0,39907	1,31137	0,02316	2,04531	0,29459	1,19377
388	A0A6N0BD16	Methyl-accepting chemotaxis protein II	0,32752	1,51733	0,28056	1,59300	0,12927	0,75295
389	A0A6N0B252	HIT-like protein	0,60464	1,07466	0,03003	0,85801	0,78497	0,94680
390	A0A6N0AZT3	Modulator of FtsH protease HflK	0,43054	1,35703	0,08657	1,98096	0,52638	1,05390
391	A0A6N0BIP4	30S ribosomal protein S20	0,02823	0,50102	0,00483	0,40726	0,52353	0,84945
392	A0A6N0AFS9	50S ribosomal protein L20	0,29528	0,89281	0,01614	0,77607	0,07182	1,20613
393	A0A6N0AJ59	30S ribosomal protein S9	0,25031	0,86455	0,10461	1,11247	0,58685	1,08304
394	A0A6N0AEN6	Histidine--tRNA ligase	0,41102	0,94204	0,61370	1,06067	0,45529	1,07303
395	A0A6N0AJY7	50S ribosomal protein L27	0,80876	1,00705	0,00034	0,79024	0,48807	1,02011
396	A0A6N0AJX9	RNA polymerase sigma factor RpoD	0,27638	0,63575	0,12026	1,18116	0,20289	1,68692
397	A0A6N0BGU0	50S ribosomal protein L19	0,02011	0,77748	0,00212	0,65708	0,04845	0,84837
398	A0A6N0AUY7	Homoserine kinase	0,00101	1,27401	0,00029	0,52724	0,00908	0,68724
399	A0A6N0B7U0	Catabolite repressor/activator	0,46905	1,13625	0,94319	1,00343	0,14982	0,69827
400	A0A2K8VYS8	50S ribosomal protein L18	0,21390	0,56154	0,60237	0,80263	0,40550	0,85171
401	A0A6N0AHE2	Quinone oxidoreductase 1	0,00704	1,70640	0,04056	1,44747	0,69726	0,96983
402	A0A2K8VTM2	Multifunctional fusion protein	0,33441	1,46022	0,31766	1,51594	0,27461	1,44392
403	A0A6N0AC03	Glutathione S-transferase GstB	0,67836	0,96907	0,00736	0,78296	0,09703	0,84943
404	A0A6N0AH02	Anaerobic glycerol-3-phosphate dehydrogenase subunit B	0,44555	1,08236	0,02932	1,34314	0,23680	0,85053
405	A0A6N0B307	Glucose-6-phosphate 1-dehydrogenase	0,30543	1,36500	0,15820	1,49221	0,67221	0,89664
406	A0A6N0B2P8	Uncharacterized protein	0,76503	1,20465	0,33829	1,41584	0,36582	1,47314
407	A0A6N0B7I1	Uncharacterized protein	0,08030	1,20407	0,11817	0,81827	0,09984	0,85917
408	A0A6N0AJG4	Single-stranded DNA-binding protein	0,63195	0,97796	0,00001	0,68782	0,10736	0,87716
409	A0A6N0AT13	Carboxylesterase NlhH	0,90764	0,97512	0,00001	7,02314	0,00421	7,57025
410	A0A6N0A8R2	Penicillin-binding protein activator LpoA	0,24613	1,47365	0,00197	1,82825	0,28047	1,29253
411	A0A6N0B3Z1	Galactose-6-phosphate isomerase subunit LacB	0,11781	0,89758	0,00024	0,53754	0,00113	0,63563

412	A0A6N0AFC6	TrmH family tRNA/rRNA methyltransferase	0,64617	1,07753	0,39854	0,86869	0,09104	0,77257
413	A0A6N0AGM0	Organic hydroperoxide resistance protein OhrB	0,09839	3,16750	0,00389	6,85696	0,10624	1,80503
414	A0A6N0AG15	Cold shock-like protein CspC	0,26046	1,01874	0,87155	0,99738	0,06033	0,93775
415	A0A6N0A8K8	Biotin carboxyl carrier protein of acetyl-CoA carboxylase	0,03245	0,62300	0,15261	0,83737	0,90003	1,02591
416	A0A6N0BHN6	Signal recognition particle protein	0,36176	0,68360	0,24091	0,71818	0,78105	0,86336
417	A0A6N0AMQ7	DNA-binding protein H-NS	0,25118	2,05519	0,01139	2,09078	0,77500	0,88215
418	A0A6N0ACS3	Pectin degradation repressor protein KdgR	0,48284	0,98172	0,00309	1,22199	0,07347	1,12430
419	A0A6N0BHG2	Histidine biosynthesis bifunctional protein HisB	0,10659	1,99609	0,00234	2,52438	0,14404	1,41007
420	A0A6N0AL19	NAD(P)H-flavin reductase	0,01077	2,13966	0,91848	1,02944	0,10235	0,56386
421	A0A6N0ACI7	Translation initiation factor IF-3	0,69123	1,06852	0,08804	1,21423	0,24601	1,16913
422	A0A6N0B8Z5	50S ribosomal protein L30	0,06517	0,77994	0,81735	0,98022	0,10989	1,15046
423	A0A6N0AH68	Universal stress protein G	0,38647	0,90937	0,13506	0,82704	0,04367	0,79126
424	A0A6N0B5T5	Peptidyl-prolyl cis-trans isomerase A	0,24346	1,42609	0,92245	0,97142	0,62549	0,92956
425	A0A6N0A9R0	Molybdate-binding periplasmic protein	0,25765	0,89181	0,98752	1,00114	0,03140	0,79899
426	A0A6N0B2V1	Flagellar hook protein FlgE	0,00849	0,30426	0,36161	0,84854	0,26889	0,50927
427	A0A6N0BLV3	Uncharacterized protein	0,58655	0,82298	0,00217	1,92807	0,22693	1,56320
428	A0A6N0BJ75	50S ribosomal protein L14	0,00847	0,83116	0,00004	0,70711	0,01974	0,83056
429	A0A6N0AQX1	Integration host factor subunit alpha	0,34707	1,36404	0,01717	2,08931	0,76852	1,05921
430	A0A6N0AFW1	p-protein	0,05280	2,77294	0,22156	1,95189	0,04599	0,87874
431	A0A6N0BHE7	2-oxoglutaramate amidase	0,94801	1,02134	0,75409	1,07814	0,95859	0,97913
432	A0A6N0BLK7	2-hydroxy-3-oxopropionate reductase	0,37697	1,11631	0,04464	1,23892	0,69774	1,03397
433	A0A6N0ABX5	Methyl-accepting chemotaxis protein III	0,00426	0,56627	0,03495	0,60083	0,02724	0,33208
434	A0A6N0AL97	N-carbamoyl-D-amino acid hydrolase	0,34431	1,05749	0,03779	1,08036	0,18625	1,12638
435	A0A6N0AK76	Putative acrylyl-CoA reductase AcuI	0,64495	0,87693	0,00428	1,61795	0,22907	1,40990
436	A0A2K8W2M6	Protein GrpE	0,21424	1,14375	0,03684	0,73491	0,07136	0,84640
437	A0A6N0BI05	Phosphoadenosine phosphosulfate reductase	0,41337	1,30675	0,79505	1,09491	0,07570	0,92510
438	A0A6N0AZ21	Transcription elongation factor GreA	0,54393	0,95499	0,15058	1,12501	0,03989	1,17358
439	A0A6N0AK09	Ribosome-binding factor A	0,23679	0,67825	0,63817	0,91880	0,30363	1,41368
440	A0A6N0ATH5	Phosphoglycolate phosphatase	0,10364	0,74580	0,07855	0,66358	0,53931	0,89359
441	A0A6N0AI03	Uncharacterized protein	0,06075	1,33360	0,34083	1,09237	0,10652	0,84464
442	A0A6N0AD72	Flavodoxin	0,25980	1,15339	0,25463	1,24539	0,05258	1,23099

443	A0A6N0B1V4	Glutaredoxin-4	0,15551	1,35706	0,17351	0,66617	0,00347	0,70953
444	A0A6N0BNE7	Acetolactate synthase isozyme 2 large subunit	0,85925	0,95358	0,11157	0,74675	0,95094	1,03093
445	A0A6N0B1J2	tRNA-modifying protein YgfZ	0,19966	1,27154	0,02989	0,46674	0,00624	0,58729
446	A0A6N0AGY4	Xanthine phosphoribosyltransferase	0,72520	0,87221	0,43352	0,75059	0,76864	0,84086
447	A0A6N0ALM2	Uncharacterized protein	0,46964	1,23066	0,23241	1,17004	0,50722	1,15922
448	A0A6N0AAN4	Ferric aerobactin receptor	0,30900	0,65199	0,45302	0,75207	0,01380	2,06469
449	A0A6N0B792	2-aminoadipate transaminase	0,94654	1,02640	0,62243	1,19432	0,01329	1,26888
450	A0A6N0AST7	Putative quorum-quenching lactonase YtnP	0,86027	1,06343	0,30368	1,31066	0,87018	1,06117
451	A0A6N0AVZ4	N5-carboxyaminoimidazole ribonucleotide mutase	0,45119	0,88478	0,04053	1,17951	0,12009	1,54808
452	A0A6N0B5W1	Mannose permease IID component	0,00080	0,69340	0,03375	1,08577	0,35643	1,05671
453	A0A6N0B1N9	Outer-membrane lipoprotein carrier protein	0,58631	1,23857	0,88593	0,94998	0,85507	0,93857
454	A0A6N0AIQ5	Stringent starvation protein A	0,03857	0,91544	0,07658	0,92350	0,20661	1,08855
455	A0A6N0ATA9	Glutamate-1-semialdehyde 2,1-aminomutase	0,43551	1,03065	0,01599	0,87222	0,11480	1,13540
456	A0A6N0AH26	Shikimate kinase 1	0,52437	0,82433	0,77298	0,97234	0,82612	1,06813
457	A0A6N0B3Z7	ProP effector	0,02328	0,89222	0,00375	0,83691	0,95325	0,99651
458	A0A6N0AJL6	Glucokinase	0,57978	1,09308	0,50678	1,04541	0,67617	1,06514
459	A0A6N0BC52	6-phosphogluconolactonase	0,60501	0,88119	0,82455	1,02987	0,75117	1,07292
460	A0A6N0ADW9	Histidinol-phosphate aminotransferase	0,50248	0,94595	0,42627	0,94980	0,78898	1,02383
461	A0A6N0B4D0	Periplasmic oligopeptide-binding protein	0,02344	0,36843	0,15039	0,71945	0,58901	1,28595
462	A0A6N0ABQ5	Transcriptional regulatory protein RcsB	0,02738	2,14519	0,09929	1,70599	0,59744	0,96288
463	A0A6N0AFZ5	Uncharacterized protein	0,00088	3,67179	0,00006	3,38437	0,10083	1,27090
464	A0A6N0BK24	FKBP-type peptidyl-prolyl cis-trans isomerase SlyD	0,04425	1,29521	0,00533	0,44161	0,00179	0,64666
465	A0A6N0AHH6	Outer membrane protein assembly factor BamD	0,00923	1,93433	0,00052	2,68198	0,59210	1,12189
466	A0A6N0B906	50S ribosomal protein L29	0,70529	1,04678	0,03704	0,67049	0,01749	0,69684
467	A0A6N0AGN0	Uncharacterized protein	0,01103	0,56358	0,09898	1,15478	0,99274	1,00320
468	A0A6N0AIP0	Lipopolysaccharide export system ATP-binding protein LptB	0,69987	1,06286	0,20177	0,94970	0,65799	0,92657
469	A0A6N0AG71	2-C-methyl-D-erythritol 2,4-cyclodiphosphate synthase	0,36654	1,19272	0,00282	1,67700	0,06479	1,42250
470	A0A6N0B4G5	Lichenan-specific phosphotransferase enzyme IIB component	0,43847	1,27942	0,85916	0,94268	0,25303	0,64050
471	A0A6N0BFY8	Tyrosine--tRNA ligase	0,25858	0,81469	0,42797	1,07699	0,03911	1,44343
472	A0A6N0AGD3	Uncharacterized protein	0,68557	1,17874	0,58442	0,71540	0,86375	0,98394

473	A0A6N0AEA5	3-hydroxydecanoyl-[acyl-carrier-protein] dehydratase	0,68050	1,25122	0,01238	2,95580	0,17527	1,91329
474	A0A6N0BDC2	Uncharacterized protein	0,01873	1,39954	0,04141	1,28244	0,78276	1,01816
475	A0A6N0BGW6	NifU-like protein	0,15603	0,64142	0,02105	1,26610	0,52992	1,36387
476	A0A6N0AKX6	Coenzyme A biosynthesis bifunctional protein CoaBC	0,24637	1,61116	0,03800	2,63975	0,01016	1,95361
477	A0A6N0BGK3	Nitrogen regulatory protein P-II	0,77273	0,98429	0,00074	0,71039	0,09366	0,89774
478	A0A6N0B685	Glutathione S-transferase GstB	0,16516	1,78529	0,44634	1,39282	0,01760	0,76989
479	A0A6N0B1L8	Putative periplasmic iron-binding protein	0,00245	2,62862	0,88847	1,05380	0,26436	1,10952
480	A0A6N0AX31	Deoxyuridine 5'-triphosphate nucleotidohydrolase	0,57354	0,80857	0,20548	0,83702	0,82795	1,08500
481	A0A6N0AJ23	Glycine betaine-binding periplasmic protein	0,02590	1,59379	0,34537	1,13119	0,01188	1,51087
482	A0A6N0AFQ9	Major outer membrane lipoprotein Lpp	0,06689	0,72503	0,26554	0,92717	0,47552	0,87550
483	A0A6N0A9K9	Ferric uptake regulation protein	0,51590	1,21605	0,70006	1,06252	0,77908	1,07185
484	A0A6N0BBK3	Phosphocarrier protein HPr	0,08129	0,81151	0,02275	1,26387	0,14172	1,44375
485	A0A6N0BAH2	UDP-glucose 4-epimerase	0,00903	1,25713	0,00881	0,75569	0,00137	0,68486
486	A0A6N0AU32	Methylglyoxal synthase	0,41435	0,96014	0,00179	0,67252	0,00417	0,68694
487	A0A6N0ATR4	RNA polymerase-binding transcription factor DksA	0,08817	0,67214	0,18912	1,16375	0,17965	1,35829
488	A0A6N0AG70	Molybdopterin adenylyltransferase	0,00356	1,36181	0,00353	1,35537	0,19963	1,05606
489	A0A6N0AQY5	Glutaredoxin-1	0,15799	1,35210	0,00501	1,93892	0,03970	1,36575
490	A0A6N0AZB8	Bifunctional protein GlmU	0,08799	2,06858	0,00857	2,72209	0,60985	0,79799
491	A0A6N0B0C3	Histidinol dehydrogenase	0,00565	2,53859	0,00246	3,10964	0,24569	0,80253
492	A0A6N0B1X7	Ribosomal protein S12 methylthiotransferase accessory factor YcaO	0,30444	1,49048	0,61907	1,14001	0,66836	0,88246
493	A0A6N0BB83	Elongation factor Tu	0,00116	3,94935	0,02239	3,20459	0,25836	1,12450
494	A0A6N0AZJ6	ATP-dependent Clp protease proteolytic subunit	0,00110	1,81865	0,00032	2,06361	0,08735	1,11290
495	A0A6N0BB99	Putative L,D-transpeptidase YbiS	0,13448	1,55801	0,01197	2,50793	0,04543	1,58226
496	A0A6N0AL09	Ubiquinone/menaquinone biosynthesis C-methyltransferase UbiE	0,00521	0,66064	0,39653	0,94172	0,00036	1,47591
497	A0A6N0AX80	Phosphate-binding protein PstS	0,02142	2,65800	0,00130	4,45123	0,02257	1,43957
498	A0A6N0AWQ4	Cell division protein ZapB	0,11038	0,45473	0,18949	1,32742	0,00936	3,12950
499	A0A6N0AIW6	Coenzyme A disulfide reductase	0,04724	0,35641	0,54656	0,78919	0,17398	0,65480
500	A0A6N0AWD5	30S ribosomal protein S21	0,29345	0,58992	0,96367	0,97464	0,67739	1,27398
501	A0A6N0ATJ7	3-dehydroquinate synthase	0,07646	1,12111	0,01130	0,91483	0,07981	0,83928

502	A0A6N0BAA6	3,4-dihydroxy-2-butanone 4-phosphate synthase	0,36260	1,46403	0,39399	1,41421	0,70819	1,04859
503	A0A6N0AAD4	Putative phospholipid-binding protein MlaC	0,73784	1,14305	0,87022	1,04351	0,69072	0,83124
504	A0A6N0AV98	UDP-N-acetylmuramoyl-tripeptide--D-alanyl-D-alanine ligase	0,17324	2,47346	0,00253	3,66318	0,83605	1,10558
505	A0A6N0AEH1	Uncharacterized protein	0,02334	0,49759	0,05702	0,52897	0,66455	0,85597
506	A0A6N0AC56	Uncharacterized protein	0,22020	1,27319	0,05671	1,49145	0,35108	1,23309
507	A0A6N0AX27	Alpha-D-glucose-1-phosphate phosphatase YihX	0,01234	1,45679	0,20772	1,36614	0,13950	0,87109
508	A0A6N0BBL6	Putative phospholipid ABC transporter-binding protein MlaD	0,08919	1,88724	0,11465	2,04519	0,60063	1,13838
509	A0A6N0ACP0	Galactose/methyl galactoside import ATP-binding protein MglA	0,36230	0,55568	0,97401	1,01248	0,49298	1,63375
510	A0A6N0BJF2	Acetolactate synthase isozyme 3 small subunit	0,23084	0,78496	0,11853	0,86875	0,56061	1,13243
511	A0A6N0BAN9	30S ribosomal protein S12	0,35102	0,63804	0,20854	1,50214	0,96044	1,04526
512	A0A6N0AGF1	Putative L,D-transpeptidase YcfS	0,26337	0,65882	0,17407	0,68046	0,87768	1,06954
513	A0A6N0B721	Mannose-1-phosphate guanylyltransferase 1	0,26352	1,39471	0,69030	0,91702	0,22711	0,72221
514	A0A6N0AJG1	D-ribose-binding periplasmic protein	0,00724	0,84927	0,46102	1,02431	0,84673	0,99652
515	A0A6N0AZI4	Putative Fe(2+)-trafficking protein	0,51913	1,06941	0,01449	0,42374	0,06272	0,70725
516	A0A6N0AA70	50S ribosomal protein L33	0,08594	0,58821	0,00779	1,73184	0,93280	1,05013
517	A0A6N0AYH2	Fe/S biogenesis protein NfuA	0,06323	1,17510	0,28303	0,89612	0,79718	0,98241
518	A0A6N0AFZ8	Cell division topological specificity factor	0,28817	1,35506	0,77954	0,91318	0,00433	0,71302
519	A0A6N0B970	Glycerol uptake facilitator protein	0,77020	0,96368	0,09106	0,75395	0,24837	0,86398
520	A0A6N0AM30	Glycerol-3-phosphate acyltransferase	0,15542	0,54472	0,14368	0,61174	0,45812	1,33593
521	A0A6N0ANJ7	3-phosphoshikimate 1-carboxyvinyltransferase	0,41598	0,91896	0,00393	0,66454	0,21192	0,69156
522	A0A6N0AS92	GTPase Era	0,65420	1,23591	0,54330	1,17819	0,26997	1,59968
523	A0A6N0B3V9	PTS system glucose-specific EIICB component	0,43608	1,45713	0,78444	1,10427	0,66788	0,82466
524	A0A2K8VTW3	Uncharacterized protein	0,62556	1,32901	0,00087	5,90742	0,85526	0,87251
525	A0A6N0AY64	Spheroplast protein Y	0,87053	0,88314	0,03093	2,98408	0,39612	1,64589
526	A0A6N0BF10	Quinone oxidoreductase 2	0,01670	0,65287	0,07469	0,82348	0,01340	0,60870
527	A0A6N0B2A0	Uridylate kinase	0,02489	1,25067	0,03353	0,79208	0,00975	0,75226
528	A0A6N0AMJ6	Arsenate reductase	0,06337	1,26213	0,01831	1,19730	0,15099	0,79784
529	A0A6N0AI24	Phosphoheptose isomerase	0,82725	1,04233	0,44919	0,87103	0,53550	1,09790

530	A0A6N0BL41	ATP synthase epsilon chain	0,37679	1,38136	0,81726	0,91889	0,98863	0,99604
531	A0A6N0BKR0	Acetolactate synthase isozyme 1 large subunit	0,28172	2,75979	0,34189	1,99013	0,68961	0,76229
532	A0A6N0BFT5	T-protein	0,71645	0,88931	0,59862	0,83790	0,57314	1,16380
533	A0A2K8VWM7	Inositol-1-monophosphatase	0,00041	5,56546	0,04252	2,02630	0,01656	0,56553
534	A0A6N0AF86	ATP-dependent RNA helicase SrmB	0,00837	1,62290	0,73902	1,05927	0,40198	0,92614
535	A0A6N0ANF2	Protein-export membrane protein SecF	0,97164	1,00271	0,34480	1,05220	0,17349	1,11245
536	A0A6N0AWF8	Uncharacterized protein	0,22423	1,73269	0,30700	1,53521	0,64875	1,13721
537	A0A6N0BKV8	Iron-sulfur cluster insertion protein ErpA	0,79648	0,95906	0,38134	0,91985	0,55382	0,85395
538	A0A6N0AHH4	Elongation factor 4	0,41768	0,59545	0,58821	1,17018	0,16002	2,42501
539	A0A6N0AX39	Acetyl esterase	0,37306	0,63671	0,09783	0,37046	0,18837	0,46326
540	A0A6N0AVB8	Elongation factor P	0,45051	0,81337	0,64954	1,05641	0,49298	1,18457
541	A0A6N0ACV6	Putative methyltransferase YcgJ	0,06105	0,74068	0,43047	1,04669	0,10265	1,28414
542	A0A6N0BFL7	Tautomerase PptA	0,67553	1,10047	0,22808	1,29030	0,42161	1,22947
543	A0A6N0B8R8	Ribulose-phosphate 3-epimerase	0,02785	10,81319	0,00003	9,32933	0,76915	1,14017
544	A0A6N0B0H6	Putative phosphatase YcdX	0,29699	1,12194	0,07502	0,76763	0,12258	0,82343
545	A0A6N0A9G2	7-cyano-7-deazaguanine synthase	0,37767	1,43775	0,44415	1,35700	0,12166	1,31798
546	A0A6N0AFF8	FMN-dependent NADPH-azoreductase	0,79506	0,83402	0,57896	0,69721	0,77584	1,13984
547	A0A6N0BI27	Glucarate dehydratase	0,20904	0,70865	0,00004	0,74979	0,72093	0,83943
548	A0A6N0BJQ5	Spermidine synthase	0,62122	0,68482	0,01702	3,23428	0,00556	3,03861
549	A0A6N0BGD6	Amidophosphoribosyltransferase	0,00984	0,40784	0,30077	1,24398	0,17002	1,67002
550	A0A6N0BHW6	50S ribosomal protein L31	0,00888	0,72476	0,01704	0,77384	0,01879	1,19586
551	A0A6N0AD76	Uncharacterized protein	0,98095	0,99291	0,16934	0,67364	0,57071	0,85351
552	A0A6N0AKV1	Cytochrome bo(3) ubiquinol oxidase subunit 1	0,04524	2,87773	0,13331	3,27770	0,09351	1,46849
553	A0A6N0AGW8	30S ribosomal protein S17	0,22245	0,62088	0,17085	1,29880	0,73173	1,19377
554	A0A6N0BAJ3	30S ribosomal protein S14	0,55180	0,89190	0,06552	0,58059	0,00002	0,76389
555	A0A6N0ABD4	Multiphosphoryl transfer protein	0,82514	0,92000	0,18499	1,14656	0,84863	0,91754
556	A0A6N0AHR7	ADP compounds hydrolase NudE	0,54237	1,38445	0,42551	1,40033	0,97102	1,01718
557	A0A6N0BDL9	Exodeoxyribonuclease III	0,18640	1,42980	0,04352	1,77353	0,52606	1,17343
558	A0A6N0B783	23S rRNA (Guanosine-2'-O-)-methyltransferase RlmB	0,00245	1,33723	0,00424	1,33711	0,71401	1,02591
559	A0A6N0BJ27	Uncharacterized protein	0,68363	0,73039	0,24267	1,84068	0,04398	0,58869
560	A0A6N0APE9	Uncharacterized protein	0,04804	0,14509	0,47446	0,76015	0,49509	1,34629

561	A0A6N0BHP8	Ferritin-1	0,44706	1,40105	0,06624	2,49764	0,25142	1,99477
562	A0A6N0ALF5	DNA topoisomerase 4 subunit B	0,30861	6,01317	0,29093	1,87212	0,34197	0,22711
563	A0A6N0B5T4	NADH-quinone oxidoreductase subunit I	0,50269	0,92485	0,15838	0,78101	0,19833	0,93596
564	A0A6N0BHV7	Bifunctional protein TrpGD	0,16695	3,52327	0,00195	3,63899	0,98242	0,99001
565	A0A6N0ARD4	Uncharacterized protein	0,14678	0,92002	0,71704	1,01617	0,10269	1,08282
566	A0A6N0B4E7	Methyl-accepting chemotaxis protein II	0,06872	0,52886	0,08908	0,67265	0,65725	0,85199
567	A0A6N0ANR3	30S ribosomal protein S15	0,10136	0,25157	0,72981	0,86783	0,31935	2,10655
568	A0A6N0BJD2	4-hydroxy-3-methylbut-2-en-1-yl diphosphate synthase	0,01240	0,51574	0,00271	0,19400	0,81797	1,07944
569	A0A6N0AT58	NADP-dependent 3-hydroxy acid dehydrogenase YdfG	0,96036	0,98750	0,37734	1,10278	0,07314	0,36335
570	A0A6N0BG76	Protease HtpX	0,01342	1,25365	0,00059	0,66913	0,00160	0,66237
571	A0A6N0ARZ3	Osmotically-inducible lipoprotein E	0,76649	1,21581	0,03713	2,93218	0,88881	1,08759
572	A0A6N0AP09	Septum site-determining protein MinC	0,09629	1,36193	0,50332	0,96387	0,12076	0,75846
573	A0A6N0BLZ3	L-threonine dehydratase biosynthetic IlvA	0,27095	0,71093	0,81915	0,95478	0,17661	1,68073
574	A0A6N0AU11	Multiple stress resistance protein BhsA	0,86346	1,03958	0,29712	1,25251	0,00210	1,73790
575	A0A6N0BHA3	GTPase Der	0,58415	0,87613	0,24484	1,18669	0,75559	1,11496
576	A0A6N0AAI8	Proton glutamate symport protein	0,00273	0,85443	0,00084	1,50428	0,00003	1,35630
577	A0A6N0AZD1	L-asparaginase	0,02597	0,17964	0,38753	1,22793	0,25328	2,01876
578	A0A6N0AQA1	Flagellar motor switch protein FliM	0,09154	0,59109	0,26552	0,76298	0,01085	0,56247
579	A0A6N0B8Y3	Putative amino-acid import ATP-binding protein YxeO	0,07565	1,76359	0,38749	1,64191	0,04530	0,48199
580	A0A6N0BEU4	Long-chain fatty acid transport protein	0,93239	1,02089	0,13034	0,74218	0,45313	0,83250
581	A0A6N0AX25	Uncharacterized protein	0,69849	1,07711	0,44466	0,82814	0,39968	0,94597
582	A0A6N0ARP4	Glutamate-pyruvate aminotransferase AlaC	0,30122	2,35921	0,02905	2,41334	0,66334	1,22792
583	A0A6N0AK88	Nitrogen regulatory protein	0,69502	1,08894	0,62907	0,90488	0,89391	1,02371
584	A0A6N0BIX0	Siroheme synthase	0,08362	2,42642	0,11506	2,26844	0,43603	0,93216
585	A0A6N0ADK4	Outer membrane protein W	0,04650	0,46623	0,00088	1,55220	0,36061	1,47321
586	A0A6N0AVY3	Soluble pyridine nucleotide transhydrogenase	0,22409	0,83490	0,00591	0,59844	0,99802	0,99967
587	A0A6N0B1T1	Orotidine 5'-phosphate decarboxylase	0,06900	1,24379	0,96429	1,00378	0,08788	0,84636
588	A0A6N0AF08	D-alanyl-D-alanine carboxypeptidase DacC	0,30413	1,29422	0,58403	0,87794	0,23095	0,66411
589	A0A6N0B5I8	3-methyl-2-oxobutanoate hydroxymethyltransferase	0,03936	0,76224	0,00285	0,48424	0,00575	0,64889
590	A0A6N0BNB2	Signal recognition particle receptor FtsY	0,35862	0,60673	0,19132	0,42663	0,07954	2,44624
591	A0A6N0BI16	50S ribosomal protein L35	0,02136	1,36074	0,55123	0,94273	0,08025	1,10507

592	A0A6N0BHR4	UDP-N-acetylmuramate--L-alanine ligase	0,00601	2,98271	0,00420	2,68635	0,88895	1,01415
593	A0A6N0ALX1	Peptide chain release factor 3	0,15576	4,15470	0,00573	3,08292	0,53156	1,33255
594	A0A6N0AE66	Putative phospholipid-binding lipoprotein MlaA	0,66843	0,89323	0,58271	1,13170	0,16147	1,24500
595	A0A6N0BEQ1	Uncharacterized protein	0,62366	1,09417	0,01359	1,60475	0,20943	1,19219
596	A0A6N0APA5	Magnesium and cobalt efflux protein CorC	0,80510	1,07134	0,13008	1,40777	0,50343	1,12152
597	A0A6N0B959	Uncharacterized protein	0,24485	1,28136	0,65006	0,89804	0,88814	1,01113
598	A0A6N0B929	5-keto-4-deoxy-D-glucarate aldolase	0,02295	0,77145	0,00983	0,60049	0,09169	0,78501
599	A0A6N0B7U2	4-hydroxythreonine-4-phosphate dehydrogenase	0,59655	0,74988	0,65518	0,79075	0,84951	1,03387
600	A0A6N0AKW3	Glutathione reductase	0,35856	0,54027	0,82273	0,91233	0,47154	1,52594
601	A0A6N0AC48	UDP-glucose 4-epimerase	0,18584	0,53510	0,22875	0,56051	0,40973	1,39544
602	A0A6N0AFL0	UvrABC system protein A	0,05764	1,93109	0,37541	0,56431	0,87830	0,92307
603	A0A6N0B7F9	Delta-aminolevulinic acid dehydratase	0,28425	1,19090	0,14402	0,67852	0,39547	0,93883
604	A0A6N0B1Y6	Phosphoenolpyruvate synthase regulatory protein	0,85885	0,95119	0,64440	0,88961	0,25783	0,78301
605	A0A6N0BG84	Monooxygenase YcnE	0,23724	1,45877	0,18009	0,51759	0,08324	0,63592
606	A0A6N0B8Y8	Ribulokinase	0,08996	2,33516	0,18764	1,90142	0,17698	1,45109
607	A0A6N0AWG0	S-adenosylmethionine decarboxylase proenzyme	0,02534	2,08286	0,59349	1,16917	0,40153	0,67834
