# Supplementary material for: The Periplasmic Oxidoreductase DsbA Is Required for Virulence of the Phytopathogen Dickeya solani
Source: Int J Mol Sci. 2022 Jan 9;23(2):697. doi: 10.3390/ijms23020697 (PMC8775594; doi:10.3390/ijms23020697)
Supplement: Supplementary file 1 [file ijms-23-00697-s001.zip › Table_S2.pdf]

Index	UniProt ID	Name/Description	p-value	Fold Change
1	A0A6N0ARG9	Outer membrane protein F	0,00044	0,70945
2	A0A6N0ALD5	Glutamate synthase [NADPH] large chain	0,00279	0,52319
3	A0A6N0AV44	DNA-directed RNA polymerase subunit beta	0,03857	1,53782
4	A0A6N0AVZ8	Glycerol kinase	0,03661	0,67953
5	A0A6N0AEN7	Periplasmic oligopeptide-binding protein	0,52052	1,11447
6	A0A6N0AA30	Putative hemoglobin and hemoglobin-haptoglobin-binding protein 2	0,00015	0,72270
7	A0A6N0B4R5	Pyruvate dehydrogenase E1 component	0,00021	0,50100
8	A0A6N0A6S9	DNA-directed RNA polymerase subunit beta	0,29197	1,07288
9	A0A6N0ANI6	60 kDa chaperonin	0,00024	1,51814
10	A0A6N0AAN4	Ferric aerobactin receptor	0,02435	0,78441
11	A0A6N0BBH7	Uncharacterized protein	0,00001	0,38685
12	A0A6N0AIL9	Carbamoyl-phosphate synthase large chain	0,20879	1,13651
13	A0A6N0AJI3	Elongation factor G	0,87347	0,96834
14	A0A6N0BKU6	Chaperone protein DnaK	0,00013	1,33967
15	A0A6N0AHU9	Periplasmic dipeptide transport protein	0,00012	0,74742
16	A0A6N0BJS7	Ketol-acid reductoisomerase	0,00004	0,63541
17	A0A6N0AH33	Flagellar hook-associated protein 1	0,00000	0,03236
18	A0A6N0AUU7	Transketolase 1	0,00000	2,25714
19	A0A6N0BM05	Vitamin B12 transporter BtuB	0,01353	0,88445
20	A0A6N0BE12	Glyceraldehyde-3-phosphate dehydrogenase A	0,00570	1,68697
21	A0A6N0ABI9	Formate acetyltransferase 1	0,20928	1,25748
22	A0A6N0AG83	Flagellin 1	0,00000	0,02905
23	A0A6N0B8D8	Glycerophosphoryl diester phosphodiesterase	0,00013	1,52904
24	A0A6N0ABM2	30S ribosomal protein S1	0,23498	0,75887
25	A0A6N0AMD5	Polyribonucleotide nucleotidyltransferase	0,00749	1,47355
26	A0A6N0AF73	Isocitrate dehydrogenase [NADP]	0,00033	0,68665
27	A0A6N0BHM0	Aerobic glycerol-3-phosphate dehydrogenase	0,00415	1,15384
28	A0A6N0B4P0	Phosphoenolpyruvate synthase	0,00297	0,52008
29	A0A6N0BFA5	Outer membrane protein A	0,00000	2,67030
30	A0A6N0AIR3	Phosphoglycerate kinase	0,18998	1,19835
31	A0A6N0BBA1	5-methyltetrahydropteroyltriglutamate--homocysteine methyltransferase	0,00002	0,29804
32	A0A6N0AI53	ATP synthase subunit alpha	0,01342	1,28677
33	A0A6N0ASL9	Flagellar hook-associated protein 2	0,00000	0,02011
34	A0A6N0B1Z2	Pyruvate kinase II	0,00000	2,34597
35	A0A6N0A9K7	Protein TolB	0,03310	1,48324
36	A0A6N0BAJ7	Oligopeptidase A	0,00345	0,45297
37	A0A6N0B2Y4	Ferrichrome-iron receptor	0,00001	0,22106
38	A0A6N0BB31	Outer membrane protein assembly factor BamA	0,00110	1,32176
39	A0A6N0AV51	5-methyltetrahydropteroyltriglutamate--homocysteine methyltransferase	0,00004	2,98251
40	A0A6N0BHD7	Phosphoribosylformylglycinamidine synthase	0,02687	0,74642
41	A0A6N0B9R8	ATP synthase subunit beta	0,41630	1,26286

42	A0A6N0BAV5	Periplasmic pH-dependent serine endoprotease DegQ	0,00013	2,09866
43	A0A6N0AD88	Citrate synthase	0,05121	1,64349
44	A0A6N0BCF9	D-galactose-binding periplasmic protein	0,00096	1,43916
45	A0A6N0AGH9	Aminopeptidase N	0,42192	0,88079
46	A0A6N0ANU7	Trigger factor	0,00048	2,05102
47	A0A6N0BI17	Enolase	0,73327	0,90678
48	A0A6N0AGF6	Vitamin B12-binding protein	0,00041	0,52924
49	A0A6N0AN74	Phosphoserine aminotransferase	0,00009	1,86363
50	A0A6N0A9V6	Glutathione-binding protein GsiB	0,00179	1,43942
51	A0A6N0BB30	Outer membrane protein TolC	0,85655	1,01253
52	A0A6N0BMC3	S-adenosylmethionine synthase	0,98246	1,00125
53	A0A6N0AJT5	Uncharacterized protein	0,00001	0,03310
54	A0A6N0AXP2	Transaldolase B	0,93931	0,99655
55	A0A6N0AKK0	Glutamine synthetase	0,00158	0,86072
56	A0A6N0ADU3	Aspartate aminotransferase	0,00012	0,68783
57	A0A6N0B307	Glucose-6-phosphate 1-dehydrogenase	0,00012	2,93070
58	A0A6N0B6V8	Outer membrane protein F	0,00504	0,58453
59	A0A6N0BNN1	Leu/Ile/Val-binding protein	0,00704	0,57790
60	A0A6N0AX80	Phosphate-binding protein PstS	0,00020	2,02199
61	A0A6N0AIK9	Chaperone protein ClpB	0,02107	0,56235
62	A0A6N0BMR8	Phosphoenolpyruvate carboxykinase [ATP]	0,02504	1,16195
63	A0A6N0ATR5	Sulfite reductase [NADPH] hemoprotein beta-component	0,00336	1,42255
64	A0A6N0AZ67	Elongation factor Ts	0,62407	1,04424
65	A0A6N0AME6	Chaperone protein HtpG	0,01480	1,56737
66	A0A6N0ADG0	Glucans biosynthesis protein G	0,00004	1,74159
67	A0A6N0B8C1	Chaperone SurA	0,00030	1,72947
68	A0A6N0AAL1	6-phosphogluconate dehydrogenase, decarboxylating	0,00160	1,29617
69	A0A6N0B4D0	Periplasmic oligopeptide-binding protein	0,03677	1,08402
70	A0A6N0ACH9	Iron uptake system component EfeO	0,00010	0,37418
71	A0A6N0AY33	Uronate isomerase	0,99509	1,00284
72	A0A6N0AVI0	Cytosol aminopeptidase	0,00007	3,22370
73	A0A6N0ASH5	Aconitate hydratase 2	0,17617	1,37243
74	A0A6N0AI32	2',3'-cyclic-nucleotide 2'-phosphodiesterase/3'-nucleotidase	0,00102	1,28016
75	A0A6N0BC61	Phosphoenolpyruvate-protein phosphotransferase	0,00054	1,93401
76	A0A6N0BDZ0	Pyruvate kinase I	0,00009	1,66240
77	A0A6N0BIA0	Serine hydroxymethyltransferase	0,18334	1,41042
78	A0A6N0A8R2	Penicillin-binding protein activator LpoA	0,01343	1,18827
79	A0A6N0BFC9	ABC transporter substrate-binding protein YesO	0,07658	0,92720
80	A0A2K8VVV2	Phosphate acetyltransferase	0,00005	1,92334
81	A0A6N0B6A4	Uncharacterized protein	0,00001	1,94663
82	A0A6N0B776	Dihydrolipoyl dehydrogenase	0,14617	0,78455
83	A0A6N0B698	Pectinesterase A	0,00000	0,15439
84	A0A6N0AF95	Periplasmic serine endoprotease DegP	0,00013	3,85071
85	A0A6N0AQD6	Glutamate/aspartate periplasmic-binding protein	0,00061	0,79791

86	A0A6N0ARJ8	Sulfate-binding protein	0,00030	2,18138
87	A0A6N0A824	Argininosuccinate synthase	0,06678	0,65185
88	A0A6N0BEK9	Fe(3+)-binding periplasmic protein	0,09655	0,93353
89	A0A6N0ABE3	Uncharacterized protein	0,54392	1,02042
90	A0A6N0BAU3	Cysteine synthase A	0,00012	1,51117
91	A0A6N0BBU2	Periplasmic dipeptide transport protein	0,11524	0,84749
92	A0A6N0BCA4	Uncharacterized protein	0,02670	1,26481
93	A0A6N0BHM4	Lysine-arginine-ornithine-binding periplasmic protein	0,34383	1,11343
94	A0A6N0BAF6	Mannan endo-1,4-beta-mannosidase	0,00000	0,14331
95	A0A6N0BBY6	Putrescine-binding periplasmic protein	0,08121	0,86991
96	A0A6N0BEU4	Long-chain fatty acid transport protein	0,01157	0,72152
97	A0A6N0B5T1	Uncharacterized protein	0,00000	0,01166
98	A0A6N0AEA4	Pectate lyase A	0,00000	0,01184
99	A0A6N0AW53	Uncharacterized protein	0,00013	1,49417
100	A0A6N0B8Q9	Bifunctional purine biosynthesis protein PurH	0,00083	3,03472
101	A0A6N0ANC2	Pectate lyase L	0,00000	0,02552
102	A0A6N0AE52	Adenylosuccinate lyase	0,87382	1,01124
103	A0A6N0AL83	DNA gyrase subunit A	0,00181	2,89424
104	A0A6N0BJ90	Pectate lyase C	0,00001	0,02266
105	A0A6N0BEF2	Endoglucanase Z	0,00000	0,02551
106	A0A6N0AJK1	Aspartate ammonia-lyase	0,03633	0,74860
107	A0A6N0AAE7	Phosphoglucomutase	0,00005	1,64377
108	A0A2K8VTD8	Arginine--tRNA ligase	0,00007	3,05139
109	A0A6N0B672	Outer membrane protein assembly factor BamB	0,00009	1,77151
110	A0A6N0AJK7	Aspartate-semialdehyde dehydrogenase	0,15512	0,82089
111	A0A6N0ARF7	Pectate lyase E	0,00000	0,01809
112	A0A6N0AQA3	Outer membrane protein assembly factor BamC	0,00073	1,66593
113	A0A6N0ALM2	Uncharacterized protein	0,03820	1,19949
114	A0A6N0ACI6	Ribose-phosphate pyrophosphokinase	0,00245	2,31775
115	A0A6N0B1L8	Putative periplasmic iron-binding protein	0,16663	0,93383
116	A0A6N0AL81	Uncharacterized protein	0,11071	1,69113
117	A0A6N0B712	DNA-directed RNA polymerase subunit alpha	0,00004	2,04901
118	A0A6N0APB6	Succinyl-CoA ligase [ADP-forming] subunit beta	0,00597	1,88985
119	A0A6N0BGT3	Uncharacterized protein	0,00000	0,04035
120	A0A6N0BES5	2,3-bisphosphoglycerate-dependent phosphoglycerate mutase	0,32501	1,02335
121	A0A6N0AIL4	Putative amino-acid ABC transporter-binding protein	0,03925	1,68009
122	A0A6N0B818	Fructose-bisphosphate aldolase class 2	0,00001	0,46782
123	A0A6N0BBN5	Trifunctional nucleotide phosphoesterase protein YfkN	0,07358	1,11001
124	A0A6N0B8G8	D-ribose-binding periplasmic protein	0,01083	1,25106
125	A0A6N0ARJ9	L-arabinose-binding periplasmic protein	0,00023	2,13653
126	A0A6N0A6E0	Superoxide dismutase [Mn]	0,01780	0,85279
127	A0A6N0AVH0	Threonine synthase	0,00019	1,85779
128	A0A6N0AD51	Leucine--tRNA ligase	0,85259	1,04075
129	A0A6N0AYX3	Malate dehydrogenase	0,03871	0,60672
130	A0A6N0B1Z5	Major phosphate-irrepressible acid phosphatase	0,00474	0,63899
131	A0A6N0B8P1	Triosephosphate isomerase	0,00070	1,37997

132	A0A6N0A8Q4	Glutamate synthase [NADPH] small chain	0,49606	0,69635
133	A0A6N0AL00	3-carboxy-cis,cis-muconate cycloisomerase	0,67415	0,78621
134	A0A6N0AH90	Uncharacterized protein	0,16046	0,47176
135	A0A6N0AE83	Uncharacterized protein	0,00005	0,08753
136	A0A6N0AT47	Glucose-6-phosphate isomerase	0,02271	1,16105
137	A0A6N0AT12	Peptidase B OS=Dickeya solani	0,00467	1,65743
138	A0A6N0AHF5	50S ribosomal protein L5	0,23747	1,49892
139	A0A6N0A9I2	Ferrichrome-iron receptor	0,29532	0,95220
140	A0A6N0AYD1	Uncharacterized protein	0,02037	2,05184
141	A0A6N0ADN0	Glutamine-binding periplasmic protein	0,00157	0,73643
142	A0A6N0AY64	Spheroplast protein Y	0,00000	11,35961
143	A0A6N0BFC7	Vitamin B12-binding protein	0,00002	0,38407
144	A0A6N0AIJ2	3-isopropylmalate dehydrogenase	0,01753	0,79548
145	A0A6N0B591	3-oxoacyl-[acyl-carrier-protein] synthase 1	0,00591	1,09492
146	A0A6N0B031	50S ribosomal protein L1	0,00873	1,29226
147	A0A6N0BLK7	2-hydroxy-3-oxopropionate reductase	0,23747	1,13073
148	A0A6N0AIU7	Carbamoyl-phosphate synthase small chain	0,00282	1,44365
149	A0A6N0AI34	Inorganic pyrophosphatase	0,00186	1,35488
150	A0A6N0AN25	Valine--tRNA ligase	0,00072	3,20859
151	A0A6N0ASY5	Adenylosuccinate synthetase	0,00791	1,35481
152	A0A6N0BGV2	Periplasmic beta-glucosidase	0,02725	1,96385
153	A0A6N0AFA2	Uncharacterized protein	0,00011	2,71298
154	A0A6N0AVV3	Uncharacterized protein	0,07958	1,83497
155	A0A6N0AG29	Phenylalanine--tRNA ligase beta subunit	0,01168	1,98555
156	A0A6N0B9F4	General L-amino acid-binding periplasmic protein AapJ	0,00225	0,73859
157	A0A6N0BIQ5	50S ribosomal protein L2	0,01108	0,77196
158	A0A6N0A8I6	NADP-dependent malic enzyme	0,85246	0,95246
159	A0A6N0ADS1	Gamma-glutamyltranspeptidase	0,00005	0,15474
160	A0A6N0ACU7	Mannose-6-phosphate isomerase	0,00183	4,53431
161	A0A6N0AFV0	Uncharacterized protein	0,00690	0,01895
162	A0A2K8VTS7	Putative cellulase	0,00002	0,04015
163	A0A6N0B7G9	Xaa-Pro dipeptidase	0,00035	1,79778
164	A0A6N0BIQ6	Phosphomannomutase/phosphoglucomutase	0,00014	2,49935
165	A0A6N0BCR8	Diaminobutyrate--2-oxoglutarate aminotransferase	0,05166	0,58813
166	A0A6N0AX16	Glutathione-binding protein GsiB	0,02530	1,30074
167	A0A6N0ADK6	Flagellar hook-associated protein 3	0,00000	0,04578
168	A0A6N0AV63	Argininosuccinate lyase	0,01191	0,72950
169	A0A6N0B974	6-phosphofructokinase isozyme 1	0,00097	2,11043
170	A0A6N0BBW4	Pectin lyase	0,00001	0,04080
171	A0A6N0AGQ9	FKBP-type peptidyl-prolyl cis-trans isomerase FkpA	0,05775	1,14254
172	A0A6N0AGA6	Uncharacterized protein	0,20103	0,71541
173	A0A6N0A6Y4	Uncharacterized protein	0,00821	1,33347
174	A0A6N0ARC9	Uncharacterized protein	0,00001	0,17854
175	A0A6N0API8	2-hydroxypropyl-CoM lyase	0,25363	0,67544
176	A0A6N0AJ09	Cystine-binding periplasmic protein	0,00128	2,33367
177	A0A6N0B1V3	NADPH dehydrogenase	0,00044	6,09196
178	A0A6N0AFN4	Putative oxidoreductase YdgJ	0,00044	3,41152

179	A0A6N0B4U8	Flagellar hook-length control protein	0,00130	0,02556
180	A0A6N0AIR5	Osmotically-inducible protein Y	0,00000	2,44137
181	A0A6N0ADT1	Lysine-arginine-ornithine-binding periplasmic protein	0,00102	0,88497
182	A0A6N0B2P8	Uncharacterized protein	0,00014	1,76840
183	A0A6N0AGC2	Negative regulator of flagellin synthesis	0,00000	0,02378
184	A0A6N0AGR8	30S ribosomal protein S4	0,00009	1,63237
185	A0A6N0BBS5	Outer membrane protein X	0,11278	1,11250
186	A0A6N0AIZ0	50S ribosomal protein L7/L12	0,00001	0,34736
187	A0A6N0AC21	Asparagine synthetase B [glutamine-hydrolyzing]	0,43601	0,30249
188	A0A6N0ADW9	Histidinol-phosphate aminotransferase	0,00797	2,28204
189	A0A6N0BJB5	GMP synthase [glutamine-hydrolyzing]	0,21094	1,35247
190	A0A6N0ABD6	ABC transporter arginine-binding protein 2	0,00009	2,12469
191	A0A6N0AKS4	Putative peroxiredoxin	0,00028	1,58675
192	A0A6N0AZN5	Cystine-binding periplasmic protein	0,00010	0,49364
193	A0A6N0AW17	Uncharacterized protein	0,03653	0,67517
194	A0A6N0AKD8	Thiol:disulfide interchange protein DsbA	0,00002	0,50583
195	A0A6N0AUB8	Enoyl-[acyl-carrier-protein] reductase [NADH] FabI	0,45218	0,91920
196	A0A6N0BGD8	Oligogalacturonate-specific porin KdgM	0,15384	1,14770
197	A0A6N0B831	50S ribosomal protein L4	0,57136	1,28709
198	A0A6N0B651	Exo-poly-alpha-D-galacturonosidase	0,00027	0,12796
199	A0A6N0AIX9	ATP synthase gamma chain	0,69215	1,06854
200	A0A6N0BBN8	Translation initiation factor IF-2	0,47932	0,83419
201	A0A6N0AG11	Tail-specific protease	0,00097	1,62489
202	A0A6N0AYC1	Peptidoglycan-associated lipoprotein	0,00031	1,50498
203	A0A6N0BBU3	4-hydroxy-tetrahydrodipicolinate synthase	0,00515	1,32859
204	A0A6N0AJ33	Proline--tRNA ligase	0,00880	1,97441
205	A0A6N0AKT8	2,3,4,5-tetrahydropyridine-2,6-dicarboxylate N-succinyltransferase	0,00760	0,76426
206	A0A6N0AMJ7	Phosphopentomutase	0,04791	1,52171
207	A0A6N0BIX2	Branched-chain-amino-acid aminotransferase	0,00126	1,85915
208	A0A6N0ARS9	Protein RecA	0,00056	2,38122
209	A0A6N0ALN3	Histidine-binding periplasmic protein	0,00264	1,43639
210	A0A6N0BAS1	Ferrichrome-iron receptor	0,17972	0,77808
211	A0A6N0BI02	50S ribosomal protein L9	0,78104	1,02108
212	A0A6N0AJ23	Glycine betaine-binding periplasmic protein	0,00833	1,29512
213	A0A6N0ARE9	NAD(P)H nitroreductase YdjA	0,01451	5,20197
214	A0A6N0A805	Purine nucleoside phosphorylase DeoD-type	0,34515	1,11907
215	A0A6N0AU66	Glutamate--tRNA ligase	0,76076	1,16056
216	A0A6N0B8L0	GDP-mannose 4,6-dehydratase	0,04102	0,68840
217	A0A6N0AJL6	Glucokinase	0,01234	1,37244
218	A0A6N0AJG1	D-ribose-binding periplasmic protein	0,00552	1,48139
219	A0A6N0AWP5	Cystathionine gamma-synthase	0,00391	1,12524
220	A0A6N0AFN3	Phage shock protein A	0,05404	1,26535
221	A0A6N0BHZ7	D-3-phosphoglycerate dehydrogenase	0,00004	0,12933
222	A0A6N0B632	General stress protein 69	0,00015	0,26867
223	A0A6N0BEU3	Uncharacterized protein	0,00051	0,05840
224	A0A6N0A9X9	Uncharacterized protein	0,29179	1,60722
225	A0A6N0BIW1	Murein hydrolase activator NlpD	0,05750	1,84012

226	A0A6N0A9Y2	Alkyl hydroperoxide reductase subunit C	0,00001	8,47398
227	A0A6N0ART2	Asparagine--tRNA ligase	0,09226	0,67784
228	A0A6N0BMB3	30S ribosomal protein S5	0,00008	1,58921
229	A0A6N0BB99	Putative L,D-transpeptidase YbiS	0,00027	3,88835
230	A0A6N0AIN6	Uncharacterized protein OS=Dickeya solani	0,00000	0,07834
231	A0A6N0AVE9	50S ribosomal protein L6	0,36193	0,94621
232	A0A6N0A9J6	LPS-assembly lipoprotein LptE	0,00208	2,05331
233	A0A6N0ACV5	Uncharacterized protein	0,00039	1,79183
234	A0A6N0AXS3	Uncharacterized protein	0,30887	1,05255
235	A0A6N0AZU4	D-methionine-binding lipoprotein MetQ	0,00282	1,43592
236	A0A6N0ASJ1	Thioredoxin reductase	0,00065	3,23443
237	A0A6N0AWY5	Putative 2-keto-3-deoxy-galactonate aldolase YagE	0,00028	4,61865
238	A0A6N0AYR4	Catalase-peroxidase	0,00639	1,91664
239	A0A6N0BA21	Glutamine--tRNA ligase	0,10664	1,23689
240	A0A6N0ACS4	Alcohol dehydrogenase 2	0,00195	1,51279
241	A0A6N0AUY9	Putative amino-acid-binding protein YxeM	0,87164	1,07489
242	A0A6N0ACS3	Pectin degradation repressor protein KdgR	0,00040	0,52169
243	A0A6N0B8V3	Acetyl-coenzyme A synthetase	0,94653	0,96267
244	A0A6N0A8J4	Uncharacterized protein	0,03091	0,87009
245	A0A6N0B8V4	Multiple sugar-binding protein	0,03779	1,23375
246	A0A6N0AXW9	50S ribosomal protein L10	0,00011	0,52239
247	A0A6N0AQQ9	Aspartate--tRNA ligase	0,01125	2,36257
248	A0A6N0ARF6	DNA-binding protein H-NS	0,27952	1,15427
249	A0A6N0AGF3	Isoleucine--tRNA ligase	0,00031	2,93941
250	A0A6N0AXG5	30S ribosomal protein S2	0,00210	1,23463
251	A0A6N0BHE7	2-oxoglutaramate amidase	0,00212	1,61565
252	A0A6N0AHH6	Outer membrane protein assembly factor BamD	0,02623	1,45964
253	A0A6N0AFL4	Tryptophan biosynthesis protein TrpCF	0,02756	3,67574
254	A0A6N0AKW2	LOG family protein YgdH	0,01004	0,70201
255	A0A6N0AM92	Microbial collagenase	0,00000	0,08182
256	A0A6N0ADP3	Fumarate hydratase class II	0,04544	1,67209
257	A0A6N0BG99	Nucleoid-associated protein YejK	0,00069	5,57445
258	A0A6N0AF63	1-(5-phosphoribosyl)-5-[(5-phosphoribosylamino)methylideneamino] imidazole-4-carboxamide isomerase	0,01303	0,52842
259	A0A6N0AS90	4-hydroxy-tetrahydrodipicolinate synthase	0,00047	2,16165
260	A0A6N0B2V4	3-deoxy-manno-octulosonate cytidylyltransferase	0,00010	3,14378
261	A0A6N0ATA7	30S ribosomal protein S8	0,00083	0,49875
262	A0A6N0B9Z7	Osmotically-inducible protein Y	0,00006	3,95698
263	A0A6N0AU95	Glucose-1-phosphatase	0,48577	0,75441
264	A0A6N0BH82	Alanine--tRNA ligase	0,26088	0,76362
265	A0A6N0BHP8	Ferritin-1	0,21912	2,33647
266	A0A6N0A9R0	Molybdate-binding periplasmic protein	0,86961	1,01215
267	A0A6N0AZK5	Adenylate kinase	0,00423	1,45919
268	A0A6N0ASE7	Soluble aldose sugar dehydrogenase YliI	0,00362	2,51872
269	A0A6N0BJ85	50S ribosomal protein L3	0,34639	1,09421
270	A0A6N0AST7	Putative quorum-quenching lactonase YtnP	0,01057	1,58881
271	A0A6N0A9K2	Succinyl-CoA ligase [ADP-forming] subunit alpha	0,16098	1,11586

272	A0A6N0A8E0	D-ribose-binding periplasmic protein	0,00032	0,23966
273	A0A6N0AJL8	Tryptophan--tRNA ligase	0,04972	1,41421
274	A0A6N0ARP5	Glutamate--cysteine ligase	0,25790	1,28752
275	A0A6N0B1N9	Outer-membrane lipoprotein carrier protein	0,00014	2,82181
276	A0A6N0AVX9	30S ribosomal protein S3	0,00025	2,18359
277	A0A6N0BK42	Uncharacterized protein	0,00001	0,14930
278	A0A6N0BCK0	Transaldolase	0,17139	0,66760
279	A0A6N0B2V1	Flagellar hook protein FlgE	0,00004	0,12960
280	A0A6N0AC94	Glucuronoxylanase XynC	0,00032	0,14457
281	A0A6N0B5U1	4-hydroxy-tetrahydrodipicolinate reductase	0,15925	0,72037
282	A0A6N0AJ59	30S ribosomal protein S9	0,00180	1,46560
283	A0A6N0BIG4	30S ribosomal protein S7	0,90568	0,99303
284	A0A6N0AYQ7	Thiol:disulfide interchange protein DsbC	0,45668	1,13887
285	A0A6N0BJ22	50S ribosomal protein L15	0,74415	1,06645
286	A0A6N0AN19	Hemin-binding periplasmic protein HmuT	0,00222	0,44134
287	A0A6N0ATI6	Pectate lyase C	0,00000	0,04709
288	A0A6N0B030	Ribosome-recycling factor	0,11378	0,67776
289	A0A6N0AXM0	Aspartate carbamoyltransferase	0,49273	1,08862
290	A0A6N0AI45	Putative hydrolase YxeP	0,95314	1,00463
291	A0A6N0AWX1	Uncharacterized protein	0,00015	0,05906
292	A0A6N0AAD4	Putative phospholipid-binding protein MlaC	0,00007	0,48576
293	A0A6N0B5N4	30S ribosomal protein S10	0,00025	1,43126
294	A0A6N0ABF6	Serine--tRNA ligase	0,67328	1,27347
295	A0A6N0B0F6	Aldehyde-alcohol dehydrogenase	0,15771	1,50719
296	A0A6N0AU55	Uncharacterized protein	0,05098	1,67331
297	A0A6N0AU11	Multiple stress resistance protein BhsA	0,00000	4,17922
298	A0A6N0AKW3	Glutathione reductase	0,00160	1,18079
299	A0A6N0AKM9	ATP synthase subunit b	0,21257	0,55201
300	A0A6N0ARC4	Carbohydrate acetyl esterase/feruloyl esterase	0,19285	0,69622
301	A0A6N0ANV8	Phosphoribosylformylglycinamidine cyclo-ligase	0,09077	0,33377
302	A0A6N0BBM6	50S ribosomal protein L13	0,00043	1,53829
303	A0A6N0B4F8	Lipoprotein E	0,00000	2,56440
304	A0A6N0B2Q1	KHG/KDPG aldolase	0,00574	1,62848
305	A0A6N0AIQ5	Stringent starvation protein A	0,87145	1,01769
306	A0A6N0A915	N-acetyl-gamma-glutamyl-phosphate reductase	0,96880	0,98817
307	A0A6N0AZR2	Glucose-specific phosphotransferase enzyme IIA component	0,00457	1,29751
308	A0A6N0AC10	MltA-interacting protein	0,37283	1,31909
309	A0A6N0A7R6	50S ribosomal protein L11	0,03381	0,85318
310	A0A6N0APT0	Thiol peroxidase	0,00541	1,49688
311	A0A6N0AVZ9	Exopolyphosphatase	0,07318	1,25306
312	A0A6N0BEP3	Hydrogen peroxide-inducible genes activator	0,00044	3,73220
313	A0A6N0AA54	Uncharacterized protein	0,11284	1,22583
314	A0A6N0A8C2	Carboxymethylenebutenolidase	0,00001	3,48282
315	A0A6N0BGU0	50S ribosomal protein L19	0,00006	1,68418
316	A0A6N0AJ86	D-xylose-binding periplasmic protein	0,03712	1,37127
317	A0A6N0ADL9	Flagellar basal body rod protein FlgB	0,00056	0,09725
318	A0A6N0BE41	Uncharacterized protein	0,00026	1,40471

319	A0A6N0B0S2	Tryptophan synthase alpha chain	0,00037	0,51136
320	A0A6N0BJY9	50S ribosomal protein L24	0,10281	0,87814
321	A0A6N0AZJ6	ATP-dependent Clp protease proteolytic subunit	0,04426	1,45588
322	A0A6N0AEA5	3-hydroxydecanoyl-[acyl-carrier-protein] dehydratase	0,04682	1,25582
323	A0A6N0BIF8	DNA polymerase I	0,00519	2,49629
324	A0A6N0AKZ8	Uridine phosphorylase	0,09096	1,46605
325	A0A6N0B7F9	Delta-aminolevulinic acid dehydratase	0,88976	0,92742
326	A0A6N0AT52	Soluble cytochrome b562	0,00000	2,88676
327	A0A2K8VSY6	GTP cyclohydrolase 1	0,00002	2,03340
328	A0A6N0A814	Peptide chain release factor 2	0,04575	2,92790
329	A0A6N0AXE2	Uncharacterized protein	0,36434	1,26360
330	A0A6N0ARZ3	Osmotically-inducible lipoprotein E	0,00002	3,56791
331	A0A6N0AAR5	Uncharacterized protein	0,00014	0,19364
332	A0A6N0BAC9	Ornithine carbamoyltransferase chain I	0,00480	0,67850
333	A0A6N0B5D6	Flagellar hook-basal body complex protein FliE	0,00000	0,04203
334	A0A6N0AG85	Pectate lyase L	0,00168	0,01997
335	A0A6N0BLC9	Dihydrolipoyllysine-residue acetyltransferase component of pyruvate dehydrogenase complex	0,06196	0,75263
336	A0A6N0BK46	D-methionine-binding lipoprotein MetQ	0,23921	0,56588
337	A0A6N0AH46	Chemotaxis protein CheW	0,01684	0,42669
338	A0A6N0AD72	Flavodoxin	0,00817	0,79984
339	A0A6N0B675	FKBP-type 22 kDa peptidyl-prolyl cis-trans isomerase	0,00013	3,40269
340	A0A6N0B8Y8	Ribulokinase	0,03339	2,95298
341	A0A6N0B547	Flagellar basal-body rod protein FlgG	0,00056	0,06641
342	A0A6N0AQX1	Integration host factor subunit alpha	0,14134	1,34086
343	A0A6N0AT13	Carboxylesterase NlhH	0,00303	1,39112
344	A0A6N0AUL3	Putative periplasmic iron-binding protein	0,00013	5,85462
345	A0A6N0AT35	DNA topoisomerase 1	0,00158	6,59047
346	A0A6N0AGS8	S-ribosylhomocysteine lyase	0,00124	0,68193
347	A0A6N0AY12	10 kDa chaperonin	0,00825	0,72067
348	A0A6N0AE66	Putative phospholipid-binding lipoprotein MlaA	0,00000	2,11773
349	A0A6N0AKD4	Carboxymethylenebutenolidase	0,00010	2,50028
350	A0A6N0BKC3	30S ribosomal protein S6	0,00007	0,22263
351	A0A6N0BHD6	50S ribosomal protein L22	0,00386	1,33068
352	A0A6N0A9J2	Uracil phosphoribosyltransferase	0,32614	2,06481
353	A0A6N0ASX1	Biosynthetic arginine decarboxylase	0,90696	0,93812
354	A0A6N0AYE1	Uncharacterized protein	0,00000	0,06999
355	A0A6N0AQ29	Putative transcriptional regulatory protein YebC	0,57939	1,22554
356	A0A6N0AM80	50S ribosomal protein L21	0,09407	1,29016
357	A0A6N0BEI8	Chaperone protein Skp	0,10432	5,84120
358	A0A6N0A9A8	Nucleoid-associated protein YbaB	0,91516	0,95361
359	A0A6N0AJ43	Enamine/imine deaminase	0,06139	0,62947
360	A0A6N0AFS9	50S ribosomal protein L20	0,45766	0,95950
361	A0A6N0AW18	Periplasmic protein CpxP	0,00001	7,11139
362	A0A6N0ANH5	Uncharacterized protein	0,00129	2,02707
363	A0A6N0ALX3	Membrane-bound lytic murein transglycosylase C	0,00083	2,98744
364	A0A6N0AKI9	DNA-directed RNA polymerase subunit omega	0,00644	0,33822

365	A0A6N0AXL0	Multidrug efflux pump subunit AcrA	0,76665	1,48922
366	A0A6N0BHR3	Uncharacterized protein	0,00000	0,02435
367	A0A6N0AEH1	Uncharacterized protein	0,00009	2,37153
368	A0A6N0B2K0	Peptidyl-prolyl cis-trans isomerase D	0,70751	0,89072
369	A0A6N0BFR0	3-oxoacyl-[acyl-carrier-protein] reductase FabG	0,89640	1,06225
370	A0A6N0BHF7	6-phospho-beta-glucosidase GmuD	0,20028	0,89214
371	A0A6N0ABA8	Dihydrolipoyllysine-residue succinyltransferase component of 2-oxoglutarate dehydrogenase complex	0,20662	1,69361
372	A0A6N0ATH2	Acetylornithine/succinyldiaminopimelate aminotransferase	0,03989	0,78571
373	A0A6N0BGK3	Nitrogen regulatory protein P-II	0,00078	1,57466
374	A0A6N0ADF0	Uncharacterized protein	0,04179	0,38197
375	A0A6N0AEB7	Arabinogalactan endo-1,4-beta-galactosidase	0,00001	0,36077
376	A0A6N0AH49	Uncharacterized protein	0,36872	1,74291
377	A0A6N0ADA6	Outer membrane protein assembly factor BamD	0,09290	0,72585
378	A0A6N0BD87	Rare lipoprotein A	0,96251	1,02018
379	A0A6N0A8K8	Biotin carboxyl carrier protein of acetyl-CoA carboxylase	0,00075	1,37792
380	A0A6N0AJG4	Single-stranded DNA-binding protein	0,00001	1,69434
381	A0A6N0AAG6	2-oxoglutarate dehydrogenase E1 component	0,01183	1,52343
382	A0A6N0B282	Uncharacterized protein	0,02556	2,46922
383	A0A6N0BBV4	Cold shock-like protein CspE	0,00009	1,42579
384	A0A6N0AXN1	Homoserine O-acetyltransferase	0,17169	0,69841
385	A0A6N0B7Z8	Bifunctional aspartokinase/homoserine dehydrogenase 1	0,07715	1,59683
386	A0A6N0B5T5	Peptidyl-prolyl cis-trans isomerase A	0,00000	2,92175
387	A0A6N0BH40	RNA-binding protein Hfq	0,26042	0,58007
388	A0A6N0B2A0	Uridylate kinase	0,00113	1,14465
389	A0A6N0AVB9	30S ribosomal protein S13	0,00018	0,56456
390	A0A6N0AK09	Ribosome-binding factor A	0,01581	0,46461
391	A0A6N0BFR1	Flagellar basal-body rod protein FlgF	0,00002	0,12407
392	A0A6N0B1Y5	Imidazole glycerol phosphate synthase subunit HisF	0,33560	1,40108
393	A0A6N0BCD2	Uncharacterized protein	0,00151	2,99785
394	A0A6N0ANX1	Phosphoribosylaminoimidazole-succinocarboxamide synthase	0,08262	1,25701
395	A0A6N0AUY7	Homoserine kinase	0,00002	3,39501
396	A0A6N0A9L8	Cation efflux system protein CusB	0,40915	0,62992
397	A0A6N0AT23	Malonyl CoA-acyl carrier protein transacylase	0,30297	1,79475
398	A0A6N0B5T6	Gamma-glutamyl phosphate reductase	0,22182	1,21666
399	A0A6N0AHP1	Nucleoside diphosphate kinase	0,03918	1,56899
400	A0A6N0AXR2	N utilization substance protein B	0,00186	2,16978
401	A0A6N0ANQ1	Uncharacterized protein	0,00011	3,12322
402	A0A6N0ABY0	Tryptophan synthase beta chain	0,37033	1,04298
403	A0A6N0ADK4	Outer membrane protein W	0,00021	0,57689
404	A0A6N0AU32	Methylglyoxal synthase	0,71549	0,83193
405	A0A6N0BG77	Oligopeptide-binding protein AppA	0,10420	1,94230
406	A0A6N0B5V4	Disulfide-bond oxidoreductase YfcG	0,03705	1,81033

407	A0A6N0ASW1	Ribose-5-phosphate isomerase A	0,00673	0,75016
408	A0A6N0BHB7	50S ribosomal protein L17	0,00326	1,50742
409	A0A6N0B3F6	Superoxide dismutase [Cu-Zn] 1	0,02573	1,10999
410	A0A2K8VYT1	30S ribosomal protein S11	0,92745	0,97647
411	A0A6N0AWD5	30S ribosomal protein S21	0,00006	1,28532
412	A0A6N0AY10	2,5-diketo-D-gluconic acid reductase A	0,15076	1,27694
413	A0A6N0B762	Flagellar basal-body rod protein FlgC	0,00002	0,28024
414	A0A6N0AG71	2-C-methyl-D-erythritol 2,4-cyclodiphosphate synthase	0,08142	1,38156
415	A0A6N0BH43	Carbonic anhydrase 2	0,49943	1,17565
416	A0A6N0ABU4	Glucose-6-phosphate 1-epimerase	0,05059	1,77089
417	A0A6N0ADB9	Cation efflux system protein CusF	0,01174	1,39430
418	A0A6N0AH26	Shikimate kinase 1	0,00069	1,39990
419	A0A6N0B8S8	Biotin carboxylase	0,29982	0,59034
420	A0A6N0AG15	Cold shock-like protein CspC	0,10809	0,47867
421	A0A6N0ASG6	4-hydroxythreonine-4-phosphate dehydrogenase 2	0,04148	1,61519
422	A0A6N0AF60	Oligogalacturonate-specific porin KdgM	0,22385	0,72943
423	A0A2K8VTW3	Uncharacterized protein	0,00038	0,53703
424	A0A6N0BEV9	Lon protease	0,12757	0,29072
425	A0A6N0BBK2	Uncharacterized protein	0,00114	0,58423
426	A0A6N0B2L1	Serralysin A	0,03637	0,36631
427	A0A6N0AYV6	Agmatine deiminase	0,03397	1,91425
428	A0A6N0B6W9	Aerobic respiration control protein ArcA	0,14255	1,39761
429	A0A6N0AV71	Uncharacterized protein	0,01590	0,57975
430	A0A6N0AA80	DNA-binding protein HU-alpha	0,01157	0,35061
431	A0A6N0BG78	Acyl carrier protein	0,49659	1,44139
432	A0A6N0BMC4	50S ribosomal protein L16	0,00091	1,62097
433	A0A6N0BJQ1	Uncharacterized protein	0,00000	0,00792
434	A0A6N0AL97	N-carbamoyl-D-amino acid hydrolase	0,51642	1,22753
435	A0A6N0AW60	Inosine-5'-monophosphate dehydrogenase	0,81791	1,11612
436	A0A6N0B6D3	L-aspartate oxidase	0,08508	2,58766
437	A0A6N0AQ96	Ribonuclease E	0,38562	0,51969
438	A0A6N0AHQ5	Quorum-sensing regulator protein G	0,00014	3,21575
439	A0A6N0BCX3	DNA-binding protein HU-beta	0,00000	0,25156
440	A0A6N0B438	Uncharacterized protein	0,00035	0,11505
441	A0A6N0BG84	Monooxygenase YcnE	0,00088	1,75465
442	A0A6N0ADH1	Hydroperoxy fatty acid reductase gpx1	0,00444	2,06854
443	A0A6N0A8L6	Protein GrpE	0,00002	1,78255
444	A0A6N0BHP5	Outer membrane lipoprotein SlyB	0,00029	1,42034
445	A0A6N0AHQ6	Vibriobactin utilization protein ViuB	0,00729	0,38270
446	A0A6N0BJ75	50S ribosomal protein L14	0,03135	1,25313
447	A0A6N0ARB5	Phosphoribosylglycinamide formyltransferase 2	0,00010	3,48609
448	A0A6N0ACI7	Translation initiation factor IF-3	0,00015	3,78036
449	A0A6N0AII9	Thiamine-binding periplasmic protein	0,11160	1,60107
450	A0A6N0AFY6	Uncharacterized protein	0,13680	1,22117
451	A0A6N0AR82	GDP-mannose pyrophosphatase NudK	0,00000	4,81772
452	A0A6N0BF10	Quinone oxidoreductase 2	0,22364	1,15368
453	A0A6N0AWZ6	Uncharacterized protein	0,00282	0,58491

454	A0A6N0BJQ5	Spermidine synthase	0,33829	1,14891
455	A0A6N0BAN9	30S ribosomal protein S12	0,44111	0,51722
456	A0A6N0B916	Bacterioferritin	0,00351	1,22251
457	A0A6N0AFG9	30S ribosomal protein S18	0,00073	1,63537
458	A0A6N0BCL0	Uncharacterized protein	0,01495	2,55171
459	A0A6N0AL50	6,7-dimethyl-8-ribityllumazine synthase	0,44676	1,09247
460	A0A6N0AF66	Phenylalanine--tRNA ligase alpha subunit	0,11553	1,72334
461	A0A6N0ANT6	Uncharacterized protein	0,59520	0,75807
462	A0A6N0BDC0	50S ribosomal protein L25	0,12630	0,77624
463	A0A6N0BGU2	Protease PrtS	0,01138	0,27953
464	A0A6N0A6N3	Protein-export protein SecB	0,03960	0,65486
465	A0A6N0ASG4	Acetate kinase	0,01474	1,70223
466	A0A6N0AEN3	Lactoylglutathione lyase	0,10508	2,60070
467	A0A6N0B5X8	26 kDa periplasmic immunogenic protein	0,00005	2,23064
468	A0A6N0AVB8	Elongation factor P	0,45862	0,92847
469	A0A6N0AFQ9	Major outer membrane lipoprotein Lpp	0,24878	1,11283
470	A0A6N0B3P2	Ureidoglycolate lyase	0,00177	2,67918
471	A0A6N0ACB8	Uncharacterized protein	0,00003	0,12345
472	A0A6N0BKZ5	Uncharacterized protein	0,65151	0,83307
473	A0A6N0AZ21	Transcription elongation factor GreA	0,02975	1,99623
474	A0A6N0AMQ7	DNA-binding protein H-NS	0,02451	1,42436
475	A0A6N0AFU0	2-dehydro-3-deoxyphosphooctonate aldolase	0,65805	0,76295
476	A0A6N0AVX4	Dihydroxy-acid dehydratase OS=Dickeya solani	0,08981	0,26090
477	A0A6N0ANV9	Putative acyl-CoA thioester hydrolase	0,02796	3,56192
478	A0A6N0A729	Lipopolysaccharide export system protein LptA	0,19256	1,23000
479	A0A6N0A900	ADP-L-glycero-D-manno-heptose-6-epimerase	0,95236	1,00919
480	A0A6N0AWC1	Autonomous glycyl radical cofactor	0,27686	0,23583
481	A0A6N0B7S0	Ferredoxin--NADP reductase	0,30308	1,32502
482	A0A6N0AZE7	Protein translocase subunit SecD	0,00712	0,33367
483	A0A6N0BLV3	Uncharacterized protein	0,00011	0,80705
484	A0A6N0ABR0	Uncharacterized protein	0,00008	5,82192
485	A0A6N0ATR4	RNA polymerase-binding transcription factor DksA	0,00675	2,49667
486	A0A6N0B7C5	Carnitine operon protein CaiE	0,08813	1,76780
487	A0A6N0AHB1	Uncharacterized protein	0,23091	1,12132
488	A0A6N0AQ18	Molybdenum cofactor biosynthesis protein B	0,00033	2,12509
489	A0A6N0BLT4	Multiple stress resistance protein BhsA	0,00160	2,86440
490	A0A6N0AN68	NADPH-dependent 7-cyano-7-deazaguanine reductase	0,34992	1,59106
491	A0A6N0ASP1	Cysteine--tRNA ligase	0,35827	1,16102
492	A0A6N0AHE2	Quinone oxidoreductase 1	0,93388	1,04710
493	A0A6N0BAW1	Disulfide-bond oxidoreductase YghU	0,00007	4,34219
494	A0A6N0BHZ8	Uncharacterized protein	0,03704	1,58117
495	A0A2K8W3A7	Uncharacterized protein	0,00003	0,06389
496	A0A6N0AIT6	Thioredoxin-1	0,42059	1,12774
497	A0A6N0AG39	3-oxoacyl-[acyl-carrier-protein] synthase 3	0,00144	3,22220
498	A0A6N0ACT8	Extracellular phospholipase C	0,00005	0,08742
499	A0A6N0BGH3	Methyl-accepting chemotaxis protein I	0,75529	1,24544
500	A0A6N0B689	30S ribosomal protein S16	0,00010	0,56012

501	A0A6N0ADS2	Basal-body rod modification protein FlgD	0,00105	0,14239
502	A0A6N0AZ75	3-hydroxyacyl-[acyl-carrier-protein] dehydratase FabZ	0,98182	1,01670
503	A0A6N0AYU5	Regulator of ribonuclease activity A	0,17264	1,06285
504	A0A2K8VYS8	50S ribosomal protein L18	0,00916	3,14989
505	A0A2K8W5P0	Lipoprotein, putative	0,01443	1,67623
506	A0A6N0BI00	Transcriptional regulatory protein YycF	0,09709	1,29444
507	A0A6N0AQ75	Penicillin-binding protein activator LpoB	0,16405	0,86397
508	A0A6N0B2N5	Copper-exporting P-type ATPase A	0,79912	1,11945
509	A0A6N0BG21	Septum site-determining protein MinD	0,05576	3,32676
510	A0A6N0B0H6	Putative phosphatase YcdX	0,00002	3,17872
511	A0A6N0AWQ4	Cell division protein ZapB	0,02089	0,64664
512	A0A6N0BJP3	Aspartate 1-decarboxylase	0,07920	1,12601
513	A0A6N0BIP4	30S ribosomal protein S20	0,00226	2,13921
514	A0A6N0BIF9	30S ribosomal protein S19	0,01024	1,73163
515	A0A6N0AVD9	50S ribosomal protein L23	0,00950	1,68357
516	A0A6N0AMJ6	Arsenate reductase	0,00798	3,47615
517	A0A6N0BEW5	Cysteine desulfurase	0,66112	1,23324
518	A0A6N0BEQ1	Uncharacterized protein	0,63729	1,02055
519	A0A6N0B2C2	Uncharacterized protein	0,00000	0,02201
520	A0A6N0B0E4	Uncharacterized protein	0,57690	1,16908
521	A0A6N0AZN9	Murein DD-endopeptidase MepS/Murein LD-carboxypeptidase	0,00018	6,37213
522	A0A6N0AXE4	Regulator of nucleoside diphosphate kinase	0,00843	2,06528
523	A0A6N0BDL9	Exodeoxyribonuclease III	0,00056	6,34737
524	A0A6N0AVZ4	N5-carboxyaminoimidazole ribonucleotide mutase	0,22359	1,11255
525	A0A6N0AGY4	Xanthine phosphoribosyltransferase	0,90132	0,97358
526	A0A6N0AJY7	50S ribosomal protein L27	0,00600	0,29101
527	A0A6N0BBK3	Phosphocarrier protein HPr	0,25191	0,87589
528	A0A6N0BHM3	Exoribonuclease 2	0,05291	2,01878
529	A0A6N0BAZ3	Glycerol-3-phosphate transporter	0,44903	0,61204
530	A0A6N0B685	Glutathione S-transferase GstB	0,45289	0,78433
531	A0A6N0AGM0	Organic hydroperoxide resistance protein OhrB	0,03196	1,62597
532	A0A6N0BE36	Elongation factor P-like protein	0,00352	2,48128
533	A0A6N0BAH4	Peptide deformylase	0,00887	1,23498
534	A0A6N0BHM5	Ribosome-associated inhibitor A	0,01153	0,33316
535	A0A6N0ANJ7	3-phosphoshikimate 1-carboxyvinyltransferase	0,36378	1,57108
536	A0A6N0A9T3	Glyoxylate/hydroxypyruvate reductase B	0,00042	3,56936
537	A0A6N0ALR6	UTP--glucose-1-phosphate uridylyltransferase	0,30089	0,14543
538	A0A6N0AZ35	Thymidylate synthase	0,01187	4,46157
539	A0A6N0B906	50S ribosomal protein L29	0,92923	1,07191
540	A0A6N0B8Z5	50S ribosomal protein L30	0,20877	0,79562
541	A0A6N0B3Z1	Galactose-6-phosphate isomerase subunit LacB	0,00168	1,43518
542	A0A6N0BHV4	CTP synthase OS=Dickeya solani	0,05479	6,79570
543	A0A6N0BDR4	Integration host factor subunit beta	0,00038	0,11765
544	A0A6N0AXC0	Protease 3 OS=Dickeya solani	0,80467	1,13753
545	A0A6N0B929	5-keto-4-deoxy-D-glucarate aldolase	0,36903	0,76178
546	A0A6N0B520	Uncharacterized protein	0,00031	0,21246

547	A0A6N0AGU6	ABC transporter periplasmic-binding protein YtfQ	0,83241	1,05241
548	A0A6N0AGL3	Sugar phosphatase YfbT OS=Dickeya solani	0,11766	1,19936
549	A0A6N0BFL7	Tautomerase PptA	0,74154	0,85944
550	A0A6N0B972	ATP-dependent protease subunit HslV	0,00000	2,38437
551	A0A6N0B1X7	Ribosomal protein S12 methylthiotransferase accessory factor YcaO	0,00013	5,88344
552	A0A6N0AT22	Molecular chaperone Hsp31 and glyoxalase 3	0,15870	2,58930
553	A0A6N0AN16	DNA protection during starvation protein	0,00036	2,92396
554	A0A6N0AGW8	30S ribosomal protein S17	0,00002	0,29969
555	A0A6N0BKV8	Iron-sulfur cluster insertion protein ErpA	0,00508	1,59480
556	A0A6N0BH92	Thioredoxin reductase	0,00004	1,95124
557	A0A6N0B8Y3	Putative amino-acid import ATP-binding protein YxeO	0,99204	1,00295
558	A0A6N0AKA3	Uncharacterized protein	0,00739	1,56577
559	A0A6N0AC34	Hydroxylamine reductase	0,00313	5,84100
560	A0A2K8VWB4	Bifunctional protein FolD	0,00011	1,65128
561	A0A6N0AN39	Putative lipoprotein YgdR	0,00011	4,94368
562	A0A6N0AF74	Chemotaxis protein CheY	0,00118	0,06831
563	A0A6N0ACV6	Putative methyltransferase YcgJ	0,49883	1,99146
564	A0A6N0ABD4	Multiphosphoryl transfer protein	0,93187	0,95534
565	A0A6N0BJ39	D-lactate dehydrogenase	0,34003	1,36839
566	A0A6N0AYR8	Alkyl hydroperoxide reductase subunit F	0,00095	4,48836
567	A0A6N0AQ45	Copper resistance protein C	0,01450	1,39445
568	A0A6N0AGK2	NADH-quinone oxidoreductase subunit G	0,81886	1,11079
569	A0A6N0AVL3	Der GTPase-activating protein YihI	0,25195	1,07733
570	A0A6N0AV94	Enhancing lycopene biosynthesis protein 2	0,33925	0,11434
571	A0A6N0B5W5	Cyclodextrin-binding protein	0,01395	2,80562
572	A0A6N0B0C8	Uncharacterized protein	0,00021	2,48930
573	A0A6N0B3D2	Uncharacterized protein	0,00016	10,77175
